# Supplementary material for: Biperiden for prevention of post-traumatic epilepsy: A protocol of a double-blinded placebo-controlled randomized clinical trial (BIPERIDEN trial)
Source: PLoS One. 2022 Sep 9;17(9):e0273584. doi: 10.1371/journal.pone.0273584 (PMC9462738; doi:10.1371/journal.pone.0273584)
Supplement: S1 Checklist — (DOCX) [file pone.0273584.s001.docx]

SPIRIT 2013 Checklist: Biperiden Trial for Epilepsy Prevention (BIPERIDEN)

| Section/item | Item  No | Description |
| --- | --- | --- |
| **Administrative information** | | |
| Title | 1 | Biperiden for prevention of post-traumatic epilepsy: a protocol of a double-blinded placebo-controlled randomized clinical trial (BIPERIDEN trial) |
| Trial registration | 2a | ClinicalTrials.gov: NCT04945213 |
|  | 2b | All items from the World Health Organization Trial Registration Data Set   \| **Data category** \| **Information** \| \| --- \| --- \| \| Primary registry and trial identifying number \| ClinicalTrials.gov: NCT04945213 \| \| Date of registration in primary registry \| June 30, 2021 \| \| Secondary identifying numbers \| Not applicable \| \| Source(s) of monetary or material support \| Programa de Apoio ao Desenvolvimento Institucional do Sistema Único de Saúde, PROADI-SUS, Brazil (NUP 25000.014325/2021-33) \| \| Primary sponsor \| Programa de Apoio ao Desenvolvimento Institucional do Sistema Único de Saúde, PROADI-SUS, Brazil (NUP 25000.014325/2021-33) \| \| Secondary sponsor(s) \| LEM has a grant from FAPESP (18/24561-5) and from CNPq (311619/2019-3) \| \| Contact for public queries \| Carla CG Pinheiro, +55 (11) 3394-4177, carla.cgpinheiro@hsl.org.br; Maira L Foresti, +55 11 958653803, mairalforesti@gmail.com \| \| Contact for scientific queries \| Eliana Garzon, MD, PhD +55 (11) 98206-2308, eliana.garzon@hsl.org.br \| \| Public title \| Biperiden Trial for Epilepsy Prevention (BIPERIDEN) \| \| Scientific title \| Biperiden for Prevention of Epilepsy in Patients With Traumatic Brain Injury \| \| Countries of recruitment \| Brazil \| \| Health condition(s) or problem(s) studied \| Post-traumatic epilepsy \| \| Intervention(s) \| Active comparator: Biperiden (5mg of biperiden diluted in 100 ml of 0.9% saline - every 6 hours for 10 consecutive days, total of 40 doses) Placebo comparator: sterile vehicle (matching vials containing no active ingredients) \| \| Key inclusion and exclusion criteria \| Ages eligible for study: 18 to 75 years Sexes eligible for study: both Accepts healthy volunteers: no Inclusion criteria: Given informed consent within 48h of TBI, acute moderate or severe TBI, Glasgow Coma Scale (GCS) between 6 and 12 at the accident scene and/or between 3 (if sedated) and 12 at hospital admission; computerized tomography (CT) scan with acute intraparenchymal hemorrhage and/or contusion Exclusion criteria: current use of biperiden or antiseizure drugs; history of epilepsy, other conditions that increase risk factors for epilepsy (perinatal injuries, meningitis, encephalitis, neoplasia, acute stroke, neurodegenerative diseases, etc); pregnancy, glaucoma, benign prostatic hyperplasia, atrioventricular block or cardiac arrhythmias; megacolon or mechanical obstruction of the gastrointestinal tract; inability to perform follow-up visits (e.g. homeless patients); current enrolment in other clinical trial \| \| Study type \| Interventional Allocation: randomized Intervention model: parallel assignment Masking: double blind (subject, caregiver, investigator, outcomes assessor) Primary purpose: prevention Phase III \| \| Date of first enrolment \| Not yet recruiting \| \| Target sample size \| 312 \| \| Recruitment status \| Not yet recruiting \| \| Primary outcome(s) \| Cumulative incidence of unprovoked seizures counted starting 7 days after TBI (time frame: 2 years). Cumulative incidence of serious adverse events (time frame: 2 years). \| \| Key secondary outcomes \| Cumulative incidence of unprovoked seizures, epileptiform discharges and electrographic seizures (time frame: 1, 3, 6, 9, 12, 18 and 24 months). Categorization according to total score of the mRS scale (time frame: 1, 3, 6, 9, 12, 18 and 24 months), to the specific percentile rank defined for neuropsychological standard tests (time frame: 6 and 24 months), to specific and total scores of quality of life scales (time frame: 3, 6, 12, 24). Other outcomes include the mortality incidence and occurrence of non-serious adverse events during the two year protocol \| |
| Protocol version | 3 | Version:4, Date: November 8, 2021; Identifier: 5.084.793 |
| Funding | 4 | Programa de Apoio ao Desenvolvimento Institucional do Sistema Único de Saúde, PROADI-SUS, Brazil (NUP 25000.014325/2021-33) |
| Roles and responsibilities | 5a | Principal Investigator: Eliana Garzon, MD, PhD; Sociedade Beneficente de Senhoras Hospital Sírio-Libanês, São Paulo, SP, Brazil.  Principal Investigator: Luiz E Mello, MD, PhD; Universidade Federal de São Paulo  Sub-Investigator: Carla CG Pinheiro; Sociedade Beneficente de Senhoras Hospital Sírio-Libanês, São Paulo, SP, Brazil.  Sub-Investigator: Maira L Foresti, PhD; Universidade Federal de São Paulo  Sub-Investigator: Rachel Riera, PhD; Sociedade Beneficente de Senhoras Hospital Sírio-Libanês, São Paulo, SP, Brazil. |
|  | 5b | Trial sponsor: Luiz Fernando Lima Reis, PhD; Sociedade Beneficente de Senhoras Hospital Sírio-Libanês, São Paulo, SP, Brazil; luiz.reis@hsl.org.br |
|  | 5c | Study sponsor approved the study design; collection, management and submission of the report for publication |
|  | 5d | Coordinating Centre (principal investigators, sub-investigators, research assistants): responsible for the study design; collection, management, analysis, and interpretation of data; writing and submission of the report; have ultimate authority over any of these activities.  Steering Committee (three experienced independent clinic-researchers), Data Management Team (research assistant and statistician from coordinating center), external Data Monitoring Committee (three experienced independent clinic-researchers) are groups overseeing the trial. |
| Introduction |  |  |
| Background and rationale | 6a | One of the most important neurological consequences following Traumatic Brain Injury (TBI) is the development of post traumatic epilepsy (PTE). Nevertheless, there is still no effective therapeutic intervention to reduce the occurrence of PTE. In previous studies with animals’ models of epilepsy, the biperiden decreased the incidence and intensity of spontaneous epileptic seizures besides delaying their appearance (Benassi et al., 2021; Bittencourt et al., 2017). Now we want to evaluate the safety and efficacy of biperiden in preventing the development of PTE in adult patients, who have suffered moderate or severe TBI, in a large multicenter prospective study. Biperiden is an already known drug, safe, affordable and widely used to treat another neurological pathology. |
|  | 6b | There is no effective therapeutic intervention to reduce the occurrence of post traumatic epilepsy |
| Objectives | 7 | To evaluate the efficacy of biperiden as an antiepileptogenic agent in patients that suffered TBI. |
| Trial design | 8 | Interventional; type of trial: parallel group; allocation ratio 1:1; framework: ou exploratory |
| Methods: Participants, interventions, and outcomes | | |
| Study setting | 9 | Emergency care units (recruitment) and neurology centres (follow up) of public hospitals (including academic hospitals) located at different regions from Brazil. |
| Eligibility criteria | 10 | Inclusion criteria: Adults (18 to 75 years) of both sex; given informed consent within 48h of TBI, acute moderate or severe TBI, Glasgow Coma Scale (GCS) between 6 and 12 at the accident scene and/or between 3 (if sedated) and 12 at hospital admission; computerized tomography (CT) scan with acute intraparenchymal hemorrhage and/or contusion. Exclusion criteria: current use of biperiden or antiseizure drugs; history of epilepsy, other conditions that increase risk factors for epilepsy (perinatal injuries, meningitis, encephalitis, neoplasia, acute stroke, neurodegenerative diseases, etc); pregnancy, glaucoma, benign prostatic hyperplasia, atrioventricular block or cardiac arrhythmias; megacolon or mechanical obstruction of the gastrointestinal tract; inability to perform follow-up visits (e.g. homeless patients); current enrolment in other clinical trial. Those criteria will be checked by neurosurgeons at emergency care units. |
| Interventions | 11a | Within 12 h after TBI, participants will receive intravenous (i.v.) infusions of 5 mg of biperiden (1 mL; Cinetol, Cristália, Brazil) diluted in 100 mL of 0.9% saline (treatment group) or 1 mL of sterile vehicle (sodium lactate, lactic acid, sodium hydroxide and water for injections) diluted in 100 mL of 0,9% saline (placebo group), every 6 hours for 10 days after TBI, until completing 40 total doses. |
|  | 11b | Criteria for discontinuing or modifying allocated interventions for a given trial participant (eg, drug dose change in response to harms, participant request, or improving/worsening disease) |
|  | 11c | Patients can be discontinued from the protocol if they remove the consent to participate; in any health conditions that health care providers, with sponsor agreement, believe that it is to the advantage of the patient not to comply with the procedures specified in this protocol; in case of screening error when including patients with history of epilepsy predicted in the study exclusion criteria (when this finding cannot occur at the time of recruitment). |
|  | 11d | Patients will receive all standard of care treatment indicated for their clinical condition according to the hospital protocol and will not be deprived of any required treatment, including all types of anti-seizure drugs, because their participations in this study, regardless the randomization group (placebo or biperiden) or the protocol period (intervention or follow up). |
| Outcomes | 12 | Primary outcomes: Cumulative incidence of unprovoked seizures counted starting 7 days after TBI (time frame: 2 years). Cumulative incidence of serious adverse events (time frame: 2 years). Secondary outcomes: Cumulative incidence of unprovoked seizures, epileptiform discharges and electrographic seizures (time frame: 1, 3, 6, 9, 12, 18 and 24 months). Categorization according to total score of the mRS scale (time frame: 1, 3, 6, 9, 12, 18 and 24 months), to the specific percentile rank defined for neuropsychological standard tests (time frame: 6 and 24 months), to specific and total scores of quality of life scales (time frame: 3, 6, 12, 24). Other outcomes: mortality incidence and occurrence of non-serious adverse events during the two year protocol. |
| Participant timeline | 13 | Patients will be enrolled within 12 h after TBI. Intervention will occur during 10 consecutive days. Follow-up will occur at specific time windows up to 2 years (1, 3, 6, 9, 12, 18 and 24 months after TBI). |
| Sample size | 14 | Sample size calculation was performed based on previous studies using the Pocock formula. Assuming a difference in reduction of PTE of 15% between the placebo and biperiden groups at 24 months, type 1 error (alpha) of 0,05 and type 2 error (beta) of 0.10 and 20% dropout rate, 156 patients per group would be required to provide 90% power with 5% level of significance. |
| Recruitment | 15 | We intend to recruit participants with acute TBI from at least ten hospitals located at different regions from Brazil. According to epidemiological data provided by a pilot study, we estimated that the inclusion pace could be 3-5 patients/center/month leading to study completion within 42 months. We can include additional centers for achieving adequate participant enrolment to reach target sample size. |
| **Methods: Assignment of interventions (for controlled trials)** | | |
| Allocation: |  |  |
| Sequence generation | 16a | Patients will be randomly assigned to receive placebo or biperiden using a random table generated by STATA® v17 software, in a 1:1 rate, in random permuted blocks and considering the recruiting centers as a criterion for stratification. |
| Allocation concealment mechanism | 16b | The allocation concealment will be assured using the REDCap (Research Electronic Data Capture), a web-based software platform. |
| Implementation | 16c | The random sequence will be generated by a dedicated person in the coordinating center and will feed the RedCap plataform, the neurosurgeons of the participating centers will register the participants, and through the REDCap platform (neurosurgeons or pharmacists, depending on the routine of the participating center) will randomly assign the participant to an allocation number. |
| Blinding (masking) | 17a | The intervention and the placebo will be identical in appearance (color, volume and packaging). Participants, personnel and outcome assessor will be blinded to participant assignments. |
|  | 17b | Unblinding may occur only in health conditions that health care providers, with sponsor agreement, believe that it is to the advantage of the patient´s treatment to know the allocated intervention. |
| **Methods: Data collection, management, and analysis** | | |
| Data collection methods | 18a | Data will be collected in an electronic case report form (eCRF) using free web-based software platform REDCap. Data entry will be monitored by an independent researcher according to a predefined monitoring plan. Validated questionaries of quality of life will be assessed by using the Portuguese version of EuroQol three-level version (EQ-5D-3L) and EuroQol visual analogue scale (EQ VAS); standard neuropsychologic tests, including Portuguese versions of test batteries (Vocabulary, Block design, Digit Symbol-Coding, Symbol Search and Digit Span) of the Wechsler Intelligence Scale III (WAIS III); Rey–Osterrieth complex figure; Rey Auditory Verbal Learning Test (RAVLT) and Five Digit Test (FDT) also will be used. Laboratory analyses will be performed by a central laboratory (DASA, São Paulo, Brazil), which is accredited by the College of American Pathologists (CAP, Northfield, IL, USA). |
|  | 18b | Due to the COVID-19 pandemic, we anticipate performing some evaluations through telephone calls. Despite the limited clinical assessment promoted by this resource (e.g. loss of physical evaluation), it allows investigation over seizure occurrence (primary outcome). Different contact options, such as relatives phone numbers and e-mail addresses will be collected and only after many frustrated attempts of contact for different clinical assessments, patients will be considered lost in the follow up. |
| Data management | 19 | Data entry will be monitored by an independent researcher according to a predefined monitoring plan. Patient confidentiality will be ensured by using identification numbers. The frozen trial database file will be kept on file for 5 years. Most of the eCRF is formed by objective options, diminishing double data entry, and will be followed by automatic user traceability. A query plan was designed to account for double data entry and range checks for opened data values to ensure data quality. |
| Statistical methods | 20a | Primary outcome will be analysed using logistic regression, adjusting for age and sex at baseline. The result of this regression will be an odds ratio representing the relative effect of the intervention compared to the placebo. Secondary outcomes will be analysed using appropriate generalized linear models, including logistic and linear models. |
|  | 20b | Planned subgroups include type of epileptic seizures and the presence of the polymorphic ε4 allele of the apolipoprotein E gene. |
|  | 20c | All patients who receive at least one dose of randomized study drug will be included into the safety analysis and all patients who receive at least one dose of a randomized study drug and who has an evaluable follow-up data will be included in intention-to-treat analysis. Missing data will be handled by chained-equation multiple imputation methods considering relevant baseline and outcome variables. A complete case analysis will be presented as a sensitivity analysis. |
| **Methods: Monitoring** | | |
| Data monitoring | 21a | An external Data Monitoring Committee (DMC) formed by three experienced clinic-researchers will provide, independent from the sponsor and competing interests, assessment of the continuity of the study considering the safety, validity, ethical and scientific merit of the trial. |
|  | 21b | An interim analysis will be performed at 50% recruitment considering the primary outcomes. At the interim enrollment point, the DMC will consider as criteria for early interruption of the study p<0.001 for safety and p<0.0001 for efficacy analysis. The Pocock stopping boundaries, will be used as a reference rather than as a rigid rule. In addition, the DMC will also consider the effect on other secondary outcomes, in the occurrence of unpredicted adverse events and external new evidence available during trial conduction. |
| Harms | 22 | Suspected and diagnosed severe adverse events will be reviewed and adjudicated by the Ethics Committee and the external Data Monitoring Committee. |
| Auditing | 23 | Coordinator center will perform clinical trial centralized monitory every three months. |
| Ethics and dissemination | | |
| Research ethics approval | 24 | This study follows the principles of the Good Clinical Practice (GCP – ICH6 R2) in Randomized Controlled Trial (RCT). The whole protocol has been reviewed and approved by the Research Ethics Committee of Sociedade Beneficente de Senhoras, Hospital Sírio-Libanês (CAAE No. 39005920.8.1001.5461). All participating centers also must have research ethics approval by their own institutional review board. |
| Protocol amendments | 25 | Protocol amendments must be approved by all Research Ethics Committees and will be communicated to relevant parties thought documented messages (e-mails). Amendments to the protocol will be tracked and dated upon updating versions of the standard operating procedures files. |
| Consent or assent | 26a | Participants, or their relatives in the event of patient´s unconsciousness, will have to provide written informed consent within 48 hours from the beginning of the protocol. |
|  | 26b | Additional consent provisions for collection and use of participant data and biological specimens in ancillary studies - not applicable |
| Confidentiality | 27 | Patient confidentiality will be ensured by using identification numbers in an electronic case report form (eCRF) using free web-based software platform REDCap. |
| Declaration of interests | 28 | Principal investigators declare that they have no financial and other competing interests. |
| Access to data | 29 | Only the coordinator center will have access to the final trial dataset. Each principal investigator of participant centers will have access to their specific trial dataset. |
| Ancillary and post-trial care | 30 | Transport and food expenses will be reimbursed for patient´s attendance on follow up visits. Insurance will be hired to cover all participants in this research and is approved in the project budget. |
| Dissemination policy | 31a | Trial results will be communicated trough publication on scientific journals. |
|  | 31b | Authorship will follow eligibility guidelines of scientific journals. |
|  | 31c | The data that support the findings of this study are available from the  study sponsor upon reasonable request after result publication. |
| Appendices |  |  |
| Informed consent materials | 32 | Available from the study sponsor upon reasonable request |
| Biological specimens | 33 | Blood samples will be used to investigate the presence of the polymorphic ε4 allele of the apolipoprotein E gene and will be discarded after use. There are no plans for collection, laboratory evaluation, and storage of biological specimens for genetic or molecular analysis for future use in ancillary studies. |

The SPIRIT checklist is copyrighted by the SPIRIT Group under the Creative Commons “[Attribution-NonCommercial-NoDerivs 3.0 Unported](http://www.creativecommons.org/licenses/by-nc-nd/3.0/)” license.
