# Supplementary material for: Biperiden for prevention of post-traumatic epilepsy: A protocol of a double-blinded placebo-controlled randomized clinical trial (BIPERIDEN trial)
Source: PLoS One. 2022 Sep 9;17(9):e0273584. doi: 10.1371/journal.pone.0273584 (PMC9462738; doi:10.1371/journal.pone.0273584)
Supplement: S1 File — (DOCX) [file pone.0273584.s002.docx]

**Sírio – Libanês Hospital**

Institute of Teaching and Research

Social Responsibility Office - PROADI SUS

**Biperiden for the prevention of epilepsy in patients with traumatic brain injury.**

**Principal Investigators:** Eliana Garzon e Luiz Eugênio Mello

**Research Team:** Renata Mattos, Rachel Rieira, Maíra Foresti, Carla Pinheiro, Ana Cecília Alcantara, Débora Patricio e Mariane Luz

**São Paulo, 2021**

**Change control**

| **Date of Changes** | **Summary of Changes highlighted in bold** |
| --- | --- |
| **October/2021** | **Research Team:** Renata Mattos, Rachel Rieira, Maíra Foresti, Carla Pinheiro, Ana Cecília Alcantara, ***Débora Patricio*** ***e Mariane Luz*** |
| Justification: update of team members. | |
| **October/2021** | ***Exclusion criteria: inclusion of items***  ***● Vulnerable participants, without documentation and without a fixed address and/or contact with family members;***  ***● Patients without documents and of questionable age;*** |
| Justification: Added to the exclusion criteria of the research participant patients with the vulnerability profile and without documents with doubtful age, because according to the profile of the research participant of this protocol, there are several situations in which the patient enters the urgent and emergency hospital. Thus, to mitigate losses of participants in the study that provides clinical follow-up for 24 months, we added this patient profile in the exclusion criterion to facilitate the understanding of the patients' profile for the research protocol.  Another point of exclusion refers to patients without documents and with dubious adulthood, to mitigate possible deviations from the protocol, we suggest the exclusion of the participant who is without documents at the entrance of the hospital and there are doubts about his age. | |
| **October/2021** | Exclusion Criteria: Item Exclusion  ● Family history of epilepsy in first-degree relatives; |
| Justification: removed from the research participant exclusion criteria patients with a family history of epilepsy in first-degree relatives, as this criterion is not an impediment for the individual to participate in the study. It is even relevant to identify a possible genetic influence on the development of post-traumatic epilepsy in subjects with a history of close family members with epilepsy. | |
| **October/2021** | **Exclusion of the use of Medication Log to control protocol adherence the center must log prescribed and administered medication/placebo** |
| Justification: We will not use a manual control medication log, as the coordinating center is developing an electronic form (REDCap) to which the research team will have access with all the dates scheduled for the nursing team to administer the medication and an electronic form ( REDCap) for the registration of the real administered date and time and this will be the official project data. | |
| **October/2021** | Within the 12-hour period after TBI, participants in the placebo group will receive 1mL of **the same excipient used in the production of the active drug,** which will be diluted in 10 mL of 0.9% saline and slowly applied intravenously. |
| Justification: Update on the composition of the placebo that will be provided by the company Cristália, in the initial protocol the placebo composed of 1 mL of 0.9% saline would be provided by another company. However, when we get a partnership with the pharmaceutical industry cristália, we will continue with the standard produced by them. | |
| **May/2021** | **Reasearch Team: *Renata Mattos,*** Rachel Rieira, ~~Flávia Regina Bueno~~, Maíra Foresti, ***Carla Pinheiro, Ana Cecília Alcantara*** |
| Rationale: adding team members. | |
| **May/2021** | Upon inclusion, the following information will be collected in **a standardized form on a widely used electronic platform called RedCap** |
| Justification: addition of the use of an electronic platform for better monitoring of data, traceability and security. | |
| **May/2021** | Thus, 156 participants would be needed in each group, and the inclusion of 312 participants is planned. |
| Justification: New sample calculation for the study, addition of research participants for robustness of results. | |
| **May/2021** | In case of hospital discharge within a period of less than 10 days, **the participant may be included in the study analysis (by intention to treat) if he/she has used 30 doses or more of Biperiden. If the participant has not reached these 30 minimum doses, a protocol deviation notification will be made.** |
| Justification: removing the possibility of continuing treatment at home with pills, the intervention will only be carried out in a hospital environment via intravenous route. | |
| **May/2021** | In the proposed clinical trial, the dose administered to the 'biperiden group' will be one ampoule (5 mg of biperiden lactate), diluted in saline and administered for 30 to 60 minutes, every 6 hours, for 10 days, completing 40 doses. administered. The total daily dose will then be 20mg of biperiden lactate, within the limit shown on the package insert. |
| Justification: removing the possibility of continuing treatment at home with pills, the intervention will only be carried out in a hospital environment via intravenous route. | |
| **May/2021** | In case of hospital discharge within a period of less than 10 days, making it impossible to complete the protocol in the hospital environment, **the participant may be included in the study analysis (by intention to treat) if they have used 30 doses or more of Biperiden. If the participant has not reached these 30 minimum doses, a protocol deviation notification will be made.** |
| Justification: removing the possibility of continuing treatment at home with pills, the intervention will only be carried out in a hospital environment via intravenous route. | |
| **May/2021** | The participation of nine centers is estimated and it is expected that **around 3 to 5 participants per center/month will be included, which implies 14 to 18 months for the inclusion of the 312 participants**. The total estimated time for the duration of the study (10-day intervention phase followed by a 24-month follow-up phase) is 38 to 42 months, that is, advancing to the triennium following the triennium in which the project began. |
| Justification: New sample calculation for the study, addition of research participants for robustness of results. | |
| **May/2021** | The participant will be excluded from the study in cases of pregnancy, manifestation of a desire to discontinue participation, occurrence of serious adverse events that put the life of the participant at risk (at the discretion of the investigator in discussion with the monitoring committee); **if there was a screening error when including patients with a history of epilepsy/glaucoma or any other health condition prior to TBI and predicted in the study exclusion criteria (when this finding cannot occur at the time of recruitment); any other condition that, in the opinion of the investigator, would be to the advantage of the patient not following the procedures specified in this protocol.** |
| Justification: addition of patient flow information in cases of study complications such as screening failure and protocol deviation. | |
| **May/2021** | **The randomization center will make each random number available via a digital platform to which the participating center will have available access**. As soon as a participant meets the eligibility criteria and is included in the study, **a member of the team from the delegated coordinating center will access the digital platform and will have access to the bottle number of the drug to be administered.** The numerical sequence must be strictly followed and this process will be verified in the monitoring visits. **The digital platform should only be accessed immediately before the patient receives the first dose of the intervention.** The delegated coordinating center staff member will communicate with the local pharmacy to tell them the vial number to be dispensed. The local pharmacy will deliver the ampoules identified with the random numbers from the digital platform to the nurse. Both will not know the contents of the vial. |
| Justification: update of the randomization process to improve and guarantee the quality of the research protocol. | |
| **May/2021** | In view of the above, the Society forwards, below, **the complete updated schedule of the project for execution in the current triennium (2020-2023).**  All efforts will be focused on completing the study in 2023, however, if we are not successful in the patient inclusion stage, with the number of patients included below the expected for the period, an additional period may be requested in the subsequent triennium, to complete the study. |
| Justification: update of the schedule to improve and guarantee the quality of the research protocol. | |

**Study Protocol: Biperiden for the prevention of epilepsy in patients with traumatic brain injury.**

1. **JUSTIFICATION**

*Traumatic brain injury*

Definido como alteração da função cerebral ou de emergência de patologia cerebral 2% a responsável por uma força externa, de pessoas ao mundo [TCE todas as 50 mortes em milhões de pessoas no mundo que corresponde a 50 mortes em milhões de pessoas1], o que corresponde a 50 mortes em milhões de pessoas no mundo todo o mundo todo ano, sendo a principal causa de mortalidade e comorbidade em adultos em todos os países, inclusive no Brasil [Maas 2017; Ministério da Saúde 2015; Pereira 2006].

Os dados do DATASUS mostram que cerca de 125.000 pessoas/ano atendimento hospitalar devido ao TCE, o que corresponde a uma frequência de 65,7 casos/100.000 habitantes [Magalhães 2017]. Como questões adicionais, a taxa de mortalidade hospitalar para estes casos chega a 7,7%, ou seja, 5,1 mortes/100.000 casos/ano [Magalhã2017]. Os acidentes de trânsito representam a principal causa de TCE, totalizando 50% dos casos, seguidos por quedas e violência urbana [de Almeida 2016].

Estudos que mostram fortes, apesar do tamanho do impacto do TCE, ainda são estudos embasar diretrizes e avaliações de eficácia, com ensaios clínicos mostrando a eficácia do tratamento, apesar dos resultados promissores [Maas 2017].

Com relação à morbidade associada ao CE, como complicações neurológicas incluindo problemas primários primários (iniciadas após o momento do trauma, derivadas das primárias) [Faul 2015; Masel 2010]. Sobreviventes ao TCE, muitas vezes experenciam traumas físicos, psicológicos, psicológicos e cognitivos, o que causa uma redução importante na qualidade de vida e funcionalidade do indíviduo e traz um alto impacto socioeconômico [Maas 2017].

*Post-traumatic epilepsy*

Traumatic epilepsy (PTE) is a 2% patient-adjusted surgical treatment interval, with risk according to TBI severity, intervention c, after the percentage of time since it rises to 53% in the most severe cases with solutions penetrants [Anegers1998; Asikainen 1999; England 2003; Hauser 1991; Kim 2018; Raymont 2010; Salazar 2015; Temkin 1990].

After a TBI, seizures of an epileptic nature, early (within one week post-TBI) but which do not characterize EPT, may appear. The current consensus is that the crises that characterize EPT are those that appear after the first week. Seizures can be altered by acute effects of trauma, such as cerebral hemorrhage (and do not necessarily characterize epilepsy), while late seizures may depend on synaptic reorganization mechanisms [Payan ; Yablon 1993]

Most people who develop epilepsy secondary to head trauma manifest as epileptic seizures within the first two years after the trauma or injury [da Silva 1990]. As with other types of epilepsy, the causes that link TBI to EPT have not yet been fully elucidated and it is still not possible to avoid the process of epileptogenesis. We were able to find any evidence of specific treatment to prevent or reverse seizures after TBI [2019]

*TBI treatment and EPT prevention*

The therapeutic approach indicated for TBI may involve several drugs and/or surgical procedures, to care for the primary and secondary damages and essentially depends on the extent of the lesion and which areas were affected. Therapeutic advances involve drugs targeting secondary damage mechanisms, including calcium channel blockers, corticosteroids, excitatory amino acid inhibitors, N-methyl D-aspartate (NMDA) receptor antagonists, free radical scavengers, magnesium sulfate , growth factors [Salazar 1985]. Clinical trials under development using several approaches have great therapeutic potential for TBI such as erythropoietin, statins, bone marrow cells, progesterone.

Recent clinical trials have focused on neuroprotective strategies in order to prevent and/or reduce brain damage secondary to TBI [Temkin 1990]. However, none of these interventions appear to influence the occurrence of EPT [French 2013; Klein 2017; Piccena 2017; Pitkänen 2010; Temkin 2001; Temkin 2003]. Anticonvulsant substances are indicated for the control of epileptic seizures that eventually occur in an acute form, but their administration does not prevent the evolution to epilepsy [D'Ambrosio 2004].

In this context, there are no neuroprotective drugs indicated to prevent epileptogenic processes from establishing themselves after brain damage, whether traumatic, ischemic, or otherwise [Brady 2019; Temkin 2001]. Seeking to address this gap, studies with animal models have identified that drugs that alter neuronal plasticity processes, if administered under certain conditions, have the potential to modify the natural course of EPT [Bittencourt 2017].

Specifically regarding the conditions mentioned in the study by Bittencourt, et al., [2017], there is: a) the therapeutic window, that is, the interval and time between the injurious event (TBI) and the administration of the first dose of biperiden (indicated in our project as having to be equal to or less than 12 h); b) the duration of the modulation of the neuronal plasticity process, ie the duration of treatment with biperiden (indicated in our protocol as 10 days); c) the dose of biperiden, 5 mg every 6 hours. Evidence from preclinical phase studies has indicated that these three conditions above must be met for biperiden to demonstrate effectiveness as an agent capable of demonstrating an action to modify the natural course of the disease [Bittencourt 2017].

In experimental studies using models of epilepsy, biperiden, an anticholinergic used clinically for Parkinson's, was shown to have an action on neuronal plasticity, reducing the incidence and intensity of spontaneous epileptic seizures and delaying their onset [Bittencourt 2017; Gorgati 2009].

So far, a pilot study with a small sample size has been carried out to estimate the safety and effects of biperiden in this scenario. This study has now been completed, and its results have been published recently (Benassi et al., 2021). In addition, a clinical trial (NCT01048138) has been conducted, both by the same investigator (Dr. Luiz Mello).

A pilot safety study was carried out with 8 patients at Hospital São Paulo. This test, carried out between 2005 and 2007, was approved by the CEP of Hospital São Paulo (560-05) and consisted of the administration of 5 mg of biperiden (or placebo) every 6 hours and for 10 days. This study (Benassi et al., 2021) indicated the safety of biperiden under the conditions of the study being proposed by PROADI-SUS. Adverse reactions including nausea, dizziness and transient memory loss were all transient (only during medication administration).

These 8 patients received treatment or placebo control and were followed up chronically. In patients who received placebo, one to two seizures, or even death, occurred, whereas in those treated with biperiden, none died, none developed overt epileptic seizures, and only one developed abnormalities in the EEG recordings. This first study was not, however, intended to assess efficacy, but rather the safety of biperiden at the dose we injected and in the condition after TBI. We observed only a few moderate adverse effects that could be associated with the use of biperiden during the experimental protocol. Vomiting and vertigo occurred separately in two patients, but this does not differ from the expected effects already related to this drug. One patient experienced mild, transient memory loss during treatment, which could be due to head trauma or suggest a consequence of biperiden.

Similarly, another patient, who had vertigo, also had a maze concussion on imaging. Interestingly, no side effects that could potentially be associated with biperiden use were observed in one of the patients. Therefore, the results of this first study confirm that intravenous injection of biperiden can also be used safely after TBI.

The clinical trial (NCT01048138) focuses on mechanisms of action and molecular markers and has as primary outcomes the frequency of seizures and the presence of epileptic discharge on electroencephalogram during the first 10 days after TBI and the subsequent 1, 3, 6, 9, 12, 18 and 24 months. This study is carried out in a single center (Hospital das Clínicas da Faculdade de Medicina da USP) and there are no definitive or published results yet, since the study is in the final phase of execution and the data remain closed in relation to the experimental groups (placebo x treatment, double-blind study), whose stage was compromised by the new coronavirus pandemic.

However, as preliminary results until April 2020, the study had a total of 122 patients included and randomized. age and both sexes). Of this total, 23 patients have been “excluded” from the sample so far, who had some reason that met the exclusion criteria, did not confirm the inclusion criteria or were discontinued at the option of the medical team and/or non-adherence to the protocol. Another 12 patients are in “doubt” status regarding their stay in follow-up in the study for different reasons, such as: no telephone contact, transferred from hospital, changed state, among others. In these cases, the team is working to get these patients to resume follow-up, but this has not yet been done.

In addition, another 22 patients died and 65 patients (53% of the sample) remain in follow-up. Of the patients in follow-up, 13 completed 24 months of study, and of these, 5 returned for the final evaluation. As already mentioned, the new coronavirus pandemic compromised the progress of the study and as soon as authorized, patient evaluation returns will resume, even with delays in the previously stipulated times. Regarding the presence of epileptic seizures, which indicate the development of post-traumatic epilepsy, clinical seizures were confirmed in 4 patients. Two cases present reports suggestive of seizures but still need confirmation. Another 5 cases were highlighted for presenting episodes that could also be related to the crisis, but for now are less evident.

The study NCT01048138 in question has not yet had the results published, however, its methodology will serve as a basis for the methodology of the present project proposed to MS by PROADI-SUS. It is possible that we will have more information from this study in the second half of 2020.

It should be noted that there will be no double funding and that patients recruited with funds from PROADI-SUS will not be part of the other ongoing study.

The present proposed project focuses on clinical efficacy and safety and has as primary endpoints the proportion of participants who develop TPE and/or serious adverse events in the period between 7 days and 24 months after TBI, in addition to being a multicenter study, of have a larger sample size and to consider a cost-effectiveness study at the end. More information about the outcomes of this proposed project can be found under the item “outcomes”.

Additionally, Traumatic Brain Injury (TBI) is an event with serious repercussions for the individual and for society. The incidence of post-traumatic epilepsy (PTE), which can affect almost 50% of individuals after a TBI, is one of the clearest of these repercussions. Unfortunately, there is no treatment in the world that has been shown to be effective in reducing EPT. Our project has the potential to open a window that would represent an opportunity not only for the health system in Brazil, but for the world. It is an approach with great potential for innovation developed by Brazilian scientists with international leadership and recognition. The allocation of resources by PROADI-SUS will focus exclusively on clinical effectiveness and cost effectiveness will allow the inclusion of a sufficient number of patients and thus should be more conclusive.

Considering the morbidity associated with EPT, its impact on the quality of life of patients and their families, the consequences for society and the health system, as well as the costs associated with its treatment, it is essential to evaluate, through a trial, well-designed and well-conducted randomized clinical trial, the benefits and risks of an intervention to prevent it. Additionally, if the effectiveness and safety of the intervention are proven, the assessment of its cost-effectiveness becomes fundamental for its implementation in clinical practice and for its incorporation into the Unified Health System.

1. **Aims**
2. **Aim**

To evaluate the effects (benefits and risks) and cost-effectiveness of using biperiden for the prevention of post-traumatic epilepsy (PTS) in patients with traumatic brain injury (TBI) and confirmed intraparenchymal hemorrhage and/or contusion.

1. **Specidic aims**

1. To ssess the effectiveness of biperiden for preventing EPT.

2. To assess the safety of biperiden for EPT prevention.

3. To assess the cost-effectiveness of biperiden for preventing EPT.

1. **Methods**

Considering the specific objectives established, the activities described below are foreseen, detailed in relation to methodological and operational aspects.

| **Objectives 1 and 2. To assess the effectiveness and safety of biperiden after traumatic brain injury** |
| --- |

**Context**

Randomized controlled trials are considered the most appropriate and reliable primary epidemiological studies to evaluate health treatment or prevention interventions [Howick 2011]. This confidence is mainly due to the characteristics of these studies that aim to minimize the risk of bias. Thus, this was the study design chosen to answer the question of interest to the project and which can be structured, using the acronym PICO, as follows:

P (population) = subjects with moderate to severe acute TBI, with confirmed intraparenchymal hemorrhage.

I (intervention) = biperiden

C (comparator) = placebo

O (outcomes) = effectiveness and safety outcomes (detailed below)

**Design**

Multicenter, double-blind, placebo-controlled, randomized clinical trial, classified as a phase 3 clinical trial.

Study design will follow the recommendations of the Consolidated Standards of Reporting Trials (CONSORT Statement) [Schulz 2010], which despite being developed to guide reporting or publication, has also been widely used to guide the design of a randomized clinical trial. .

The study will be planned and developed with due rigor in order to minimize the occurrence of the biases cataloged in the literature so far [de Vito 2019].

Reporting of study results will follow the Consolidated Standards of Reporting Trials (CONSORT Statement) [Schulz 2010].

**Local**

The study will be planned at Hospital Sírio-Libanês and carried out in several hospitals, according to the local availability of resources necessary for the implementation of the research protocol and interest in participation. Participating centers must have access to the following structures and resources:

● TBI patient care unit;

● Diagnostic imaging unit with availability of tomography or resonance 24 hours a day;

● Electroencephalogram service

● Ability to perform neuropsychological assessment.

During the course of the study, if there is a need to recruit more patients, new centers may be included. To ensure that participating sites can carry out the study, a questionnaire to assess their ability to conduct the study (feasibility questionnaire) must be completed by the research team at each site.

**Ethical and registration aspects**

Protocol planning and study execution will follow the following recommendations and guidelines for health intervention studies:

● Declaration of Helsinki [World Medical Association 2008];

● Good Clinical Practices [Imperial College 2007]

● International Conference for Harmonization Of Technical Requirements for Registration of Pharmaceuticals for Human Use (ICH 6) [EMA 2002]

● Resolution 466/12 of the National Research Ethics Commission (CONEP) [CONEP 2012].

The study protocol was registered in the ClinicalTrials.gov clinical trial registry database (NCT04945213), after approval by the Research Ethics Committee (CEP) of Hospital Sírio-Libanês and corroboration of approval by the CEP of each of the participating hospitals.

Considering the nature of the brain injury and the study inclusion criteria for the presence of lowered consciousness, there is a possibility that patients will not be able to provide consent to participate in the study and therefore written informed consent will be obtained. of legally authorized representatives.

Considering also that the need for consent for the administration of the first doses would imply a delay in the initial doses and the impossibility of including the participant, the local CEP of Hospital Sírio-Libanês (coordinating center) was consulted and approved this protocol, where unconscious and unaccompanied patients of a guardian can still receive doses of the drug within the first 48 hours, before consent is given by their guardian. In these cases, subsequent doses should only be applied after consent from the patient, if he/she is considered fit, or from his/her guardian.

If the possibility described above is not feasible for the local CEPs of each participating center, individuals who meet the eligibility criteria, but for whom it is not possible to obtain consent to participate (by the authorized legal guardian, if the level of awareness), will not be included in the study.

It should be noted that biperiden is widely used for other indications, being considered a safe treatment, with a low frequency of adverse events associated with its use.

**Participants**

*Selection methods*

Participants will all be those seen in the emergency department of participating centers for treatment and who meet the eligibility criteria.

*Inclusion criteria*

*● Consent to participate provided by the person responsible, who must sign and date the TCLE after being guided about the study by the principal investigator or responsible, reading and agreeing to the information letter;*

*● Age between 18 and 75 years of age;*

*● Both sexes;*

*● Diagnosis of moderate to severe acute TBI;*

*● Glasgow Coma Scale (GCS) greater than 6 to 12 at hospital admission;*

*● Presence of acute intraparenchymal hemorrhage and/or contusion confirmed by magnetic resonance imaging (MRI) and/or computed tomography (CT).*

*Exclusion criteria*

• Vulnerable participants, living on the streets, without documentation and without a fixed address and/or contact with family members;

● Undocumented patients of questionable age (18-75 years) at hospital admission;

● Being on biperiden during the period of TBI occurrence;

● History of epilepsy (confirmed by medical records and/or by use of specific medication and/or referred by the legal guardian);

● History of seizures or use of antiepileptic medication;

● History of perinatal injuries, meningitis and/or encephalitis (or other proven or probable risk factor for epilepsy);

● History of neoplasia, neurodegenerative diseases; hx of cerebrovascular accident (CVA), cognitive dysfunction, benign prostatic hyperplasia, atrioventricular block or any other cardiac arrhythmia, or glaucoma;

● Gestation;

● Current participation in another clinical trial;

● Carriers of cardiac arrhythmias or glaucoma, due to the increased risk of developing adverse reactions.

To ensure compliance with the eligibility criteria, before the first dose of the drug, magnetic resonance imaging and/or computed tomography will be performed to confirm the condition of acute intraparenchymal hemorrhage. An electrocardiogram will be requested to assess the presence of arrhythmia.

After inclusion, the following information will be collected in a standardized form on a widely used electronic platform called RedCap and stored later with maintenance of anonymity:

• demographic and clinical variables (age, comorbidity profile, inventory of previous medication and habits, especially alcoholism).

● trauma-related data (mechanism, severity, types of medication).

● clinical evolution data (symptomatic, need for hospital complications and complications related to care).

*Sample size calculation*

For the sample calculation, the Pocock formula [Pocock 1993] was used for dichotomous outcomes and considering the following premises:

● Outcome: proportion of participants presenting EPT after two years.

● Expected proportion of primary outcome in control group: 25% [Anegers 1998; Asikainen 1999; Englander 2003; Hauser 1991; Raymont 2010; Salazar 2015; Temkin 1990].

● Expected proportion of primary outcome in the intervention group: 10%

● Type 1 error (alpha): 0.05

● Type 2 error (beta): 0.10

● Expected proportion of loss: 20%

Thus, 156 participants would be needed in each group, and the inclusion of 312 participants is planned.

**Intervention**

*Biperiden group*

Within 12 hours after TBI, participants in the biperiden group will receive a dose of 5 mg (1 ml of total volume) of biperiden lactate (Cinetol, Cristália, Brazil), diluted in 10 ml of 0.9 saline solution. % and applied intravenously slowly. The treatment will be repeated every 6 hours for 10 consecutive days.

In case of hospital discharge within a period of less than 10 days, the participant may be included in the study analysis (by intention to treat) if he/she has used 30 doses or more of Biperiden. If the participant has not reached these 30 minimum doses, a protocol deviation notification will be made.

In the proposed clinical trial, the dose administered to the 'biperiden group' will be one ampoule (5 mg of biperiden lactate), diluted in saline and administered for 30 to 60 minutes, every 6 hours, for 10 days, completing 40 doses. administered. The total daily dose will then be 20mg of biperiden lactate, within the limit shown on the package insert.

As additional safety information, the 20 mg/day dose is being used in the ongoing phase 3 clinical trial (approved by the CEP of Hospital das Clínicas da Faculdade de Medicina, Universidade de São Paulo), which has already included 93 participants. Partial analysis of results (not yet open) identified 22 deaths in total (risk of death=23%). Literature data estimate a mortality of up to 55% in patients with moderate to severe traumatic brain injury, depending on intracranial pressure [Teggiari 2007].

*Placebo group*

Within 12 hours after TBI, participants in the placebo group will receive 1 mL of the same excipient used in the production of the active drug, which will be diluted in 10 mL of 0.9% saline and slowly applied intravenously. The treatment will be repeated every 6 hours for 10 consecutive days. In case of hospital discharge within a period of less than 10 days, making it impossible to complete the protocol in the hospital environment, the participant may be included in the study analysis (by intention to treat) if they have used 30 doses or more of Biperiden. If the participant has not reached these 30 minimum doses, a protocol deviation notification will be made.

*Co-interventions*

Conventional supportive treatment for TBI management received by participants will follow the protocol of the participating hospital, however, it will be recommended by the coordinating center that hospitals follow the Ministry of Health guidelines for the treatment of TBI [MS 2015]. It will also be asked in the feasibility questionnaire if the hospital makes use of these guidelines and the material published by the MS will be forwarded to the research teams of the participating centers. As the generation of the allocation sequence (described in detail below) will be central and stratified by participating center, by the precepts of this type of randomization, it is expected that the differences in the co-interventions will be distributed in a similar way between the two groups, allowing that any effect observed whether due to the use or not of biperiden.

**Methods to ensure masking**

The study will be double-blind, which implies the use of appropriate methods to keep everyone involved in the study (participants, team responsible for administering the intervention, outcome assessors and statisticians) unaware of each participant's allocation group.

To this end, biperiden and placebo must have the same physical appearance (color, odor, shape) and will be packaged in identical vials, as will the saline vials used for dilution.

**Allocation sequence generation method**

Method: Participants will be randomized by a randomization center, which will be the coordinating center (HSL), to receive one of two interventions: biperiden or placebo. Randomization will be performed through an online randomization program (www.random.org or www.randomizer.org), with generation of a table of random numbers. Each number in this randomization table will correspond to one of the two interventions.

Type: stratified randomization considering TBI severity (moderate or severe) and participating center.

Ratio: The assumed randomization ratio will be 1:1.

Unit: the assumed randomization unit will be the participant.

**Method for maintaining allocation secrecy**

The randomization table will be kept completely confidential by the randomization center. The record of this medication will only be accessed by the methods team of the research coordinating center (HSL), which will not have access to study participants.

The randomization center will make each random number available via a digital platform to which the participating center will have available access.

Once a participant fulfills the eligibility criteria and is included in the study, a member of the team from the delegated coordinating center will access the digital platform and will have access to the bottle number of the drug to be administered. The numerical sequence must be strictly followed and this process will be verified in the monitoring visits. The digital platform should only be accessed immediately before the patient receives the first dose of the intervention.

The delegated coordinating center staff member will communicate with the local pharmacy to tell them the vial number to be dispensed. The local pharmacy will deliver the ampoules identified with the random numbers from the digital platform to the nurse. Both will not know the contents of the vial.

The nurse will administer the intervention to the patient as detailed in the protocol.

**Outcomes**

Primary outcomes

*Efficacy:*

● Post-traumatic epilepsy: assessed by the proportion of participants who developed PTS in the period between seven days and 24 months after TBI. The presence of TPE must be confirmed clinically. EPT will be defined as the occurrence of at least two unprovoked seizures occurring more than seven days post TBI [Verellen 2010].

*Safety:*

● Serious adverse events: proportion of participants who experienced at least one serious adverse event during 24 months after enrollment in the study. Events considered serious are defined as those that result in death, threaten life, require hospitalization or prolongation of existing hospitalization, result in disability (persistent or significant disability), or congenital anomalies (birth defects), suspected transmission of the agent. infectious through medication [Anvisa 2016]. The information regarding the occurrence of this outcome will be compiled in the individual standardized form of each participant, which will be filled in by the research team throughout the project, based on direct active interrogation of the participants and also from their spontaneous reports.

Secondary

*Efficacy:*

● Post-traumatic epilepsy: assessed by the proportion of participants who developed PTSD at the following time points: 1, 3, 6, 9, 12, and 18 months after study enrollment.

● Quality of life: assessed by the EuroQoL 5D (EQ-5D) tool [EuroQoL Group 1990] at the following assessment times: 3, 6, 12, and 24 months after enrollment in the study. The EQ-5D is a validated questionnaire, with a validated version in Portuguese, covering five health-related quality of life domains (mobility, usual care, usual activities, pain/discomfort, anxiety/depression).

● Neuropsychological function: tests will be applied that indicate the general measure of intelligence, attention, auditory-verbal and visual memory, working memory, visual-motor dexterity and cognitive flexibility. The following instruments will be applied 6 and 24 months after inclusion in the study:

● Items from the Wechsler Intelligence Scale Battery - IV [Wechlsler 2008]:

○ Digits: The subject must orally repeat a series of numerical sequences presented in direct and reverse order. Evaluates immediate memory and working memory.

○ Vocabulary: The meaning of each of the 33 words presented orally to the subject must be verbally explained. Assesses language development, word knowledge and long-term memory.

○ Cubes: Reproduce, with two-dimensional and two-color cubes, the models presented as patterns. Assesses visual perception of abstract stimuli, spatial organization, planning, visual-motor coordination, analysis and synthesis.

○ Digits and Symbols: the subject must fill in, under time pressure, a sheet containing symbols, associating them with digits according to a model presented. The application lasts approximately 60 minutes, which for the patient who has just left the hospital can be time consuming, but during the recovery period they become viable. A minimum interval of 6 months is required between applications so that there is no learning effect.

● Rey-Osterrieth complex figure: the subject is asked to copy a complex figure and, after 5 minutes and without the model, redraw it from memory. It allows assessing visuospatial organization skills, planning and strategy development, as well as memory [Oliveira 1999].

● Rey's Auditory-Verbal Learning Test. The subject is asked to repeat a list of 15 words read aloud by the examiner. The procedure is repeated 5 times. After that, a second list (distractor stimulus) is introduced. Then the subject is asked to say the original list from memory. After 15 minutes, a new recall is requested [Rey 1941].

● Five-Digit Test: investigates cognitive flexibility and inhibitory control. The subject is asked, alternately, to: (a) count the number of stimuli presented and (b) say the printed digits [Sedo 2005].

● Electroencephalographic (EEG) pattern: assessed by tracings taken shortly after TBI at 1, 3, 6, 9, 12, 18 and 24 months.

● Apolipoprotein E (ApoE) investigation, considered an important prognostic factor for the development of post-traumatic neurological sequelae. The biological material for analysis will be collected by peripheral blood, at the time of hospital admission.

*Safety:*

● Overall mortality: assessed by the proportion of participants who died after the end of the two-year follow-up period for all participants.

● Non-serious adverse events: assessed by the proportion of participants who experienced at least one non-serious adverse event at the following time points: 1, 3, 6, 9, 12, 18, and 24 months after study enrollment. The information regarding the occurrence of this outcome will be compiled in the individual standardized form of each participant, which will be filled in by the research team throughout the project, based on direct active interrogation of the participants and also from their spontaneous reports.

Insurance will be taken out to cover all participants recruited to participate in this research and is provided for in the project budget.

**Follow-up period**

Participants will be followed up for a period of 24 months from study enrollment through medical consultations 1, 3, 6, 9, 12, 18, and 24 months after study enrollment. Thus, the final data of the studies will be available after the period of this triennium.

At hospital discharge, each patient will receive a standardized epileptic seizure diary and will be instructed on how to complete it, considering the occurrence of seizures, and must present it at each follow-up appointment.

Patients will be able to undergo examinations (CT, MRI, EEG) at specific times during the hospitalization period and for 24 months after enrollment in the study. In this way, the follow-up period will advance to the three-year period following the start of the project. The evaluation moments will be detailed in the sections 'Outcomes' and 'Schedule and Activities' (item VI).

**Statistical planning and analysis**

The following analyzes will be carried out:

• Intra-group analysis: to assess the behavior of the variable over time within a single intervention arm.

• Inter-group analysis: to assess the outcomes of interest, considering the occurrence of events or average scores between the two intervention arms at all planned timepoints). These will be the analyzes of greatest interest, because through them it is possible to compare the effects of two or more interventions in an RCT.

To evaluate the characteristics of the data distribution (Gaussian or not), the Kolmogorov-Smirnov and Shapiro-Wilk tests will be applied. For the tests, their respective tables of critical values ​​will be used according to the sample and the level of significance (assumed value: 0.05). For the Kolmogorv-Smirnov test, if the value calculated by the test is greater than the critical value, the hypothesis of normality of the data will be rejected. For the Shapiro-Wilk test, the hypothesis of normality of the data will be rejected if the calculated value is less than the critical value.

To compare the average results obtained between the groups (called group effect) and also, at the same time, between two moments (1, 3, 6, 9, 12, 18 and 24 months), analysis of variance (ANOVA ) with repeated measurements, considering that the measurements over time are related to the same patient. If the data distribution is not Gaussian, the Friedman test will be used, with the Wilcoxon test for post-hoc analysis.

For dichotomous variables, the chi-square test or Fisher's exact test (less than five events in a cell of the contingency table) will be used to compare the frequency of events between the two intervention groups.

For all tests, a significance level of 5% will be considered and data will be analyzed by intention to treat (ITT) and per protocol. If there is a difference in the results found with the two analyses, the results of the ITT analysis will be considered as the main ones. In case of loss of data or participants, ITT analyzes will be carried out using appropriate methods for imputing missing data (last observation carried forward - LOCF, average of remaining participants or mixed approaches).

For dichotomous variables, the chi-square test or Fisher's exact test will be used to compare the frequency of events between the two intervention groups.

**Study duration forecast**

The participation of nine centers is estimated and it is expected that around 3 to 5 participants per center/month will be included, which implies 14 to 18 months for the inclusion of the 312 participants. The total estimated time for the duration of the study (10-day intervention phase followed by a 24-month follow-up phase) is 38 to 42 months, that is, advancing to the triennium following the triennium in which the project began.

**Interim analysis**

An interim analysis will be performed after the first 156 participants have completed 12 months of study. This will likely occur 26 to 31 months after the first participant is enrolled. Thus, the interim analysis will be conducted after the period comprising the three-year period in which the project was initiated.

**Criteria for stopping the study**

In the interim analysis, the study can be terminated due to futility if the results found indicate that even with the continuity of the study (considering the most favorable scenario for biperiden) the final results would show no benefit or, at best, a benefit clinically not relevant.

**Criteria for excluding a participant after the start of the study**

The participant will be excluded from the study in cases of pregnancy during intervention, expression of desire to discontinue participation, occurrence of serious adverse events that put the participant's life at risk (at the discretion of the investigator in discussion with the monitoring committee); if there was a screening error when including patients with a history of epilepsy/glaucoma or any other health condition prior to TBI and predicted in the study exclusion criteria (when this finding cannot occur at the time of recruitment); any other condition that, in the opinion of the investigator, would be to the advantage of the patient not following the procedures specified in this protocol.

Participants who did not complete the minimum necessary doses (30 doses), due to hospital discharge, death, or any other intercurrence that makes it impossible to complete the administration of the drug will be considered protocol deviations.

**Monitoring**

Before the start of the study, an ad hoc committee will be constituted, composed of two external researchers and an internal researcher, with the central objective of:

● Oversee the progress of the clinical trial and ensure that it is conducted, recorded, and reported in accordance with the protocol, Standard Operating Procedures (SOPs), and Good Clinical Practice [Imperial College 2007] and applicable regulatory requirements.

● Develop and update a document that describes strategies, methods, responsibilities and requirements for monitoring the clinical trial.

The monitoring committee's activities include:

- Monitor compliance between the execution of the study and the protocol, through periodic and random activities to:

○ observe the adequacy to the eligibility criteria

○ observe the adequacy to the schedule

○ observe patient/staff adherence to the use of the intervention

○ observe compliance with randomization methods and allocation secrecy

○ ensure that study data are reported accurately, completely and verifiable from source documents.

● Monitor security aspects, through periodic and random activities to:

○ ensure the rights and well-being of participants

○ occurrence of serious adverse events

○ evaluate criteria for study interruption

○ ensure adherence to research ethics and good clinical practice [ICH-6] recommendations and guidelines.

About the committee members:

● must be properly trained and must have the necessary scientific and/or clinical knowledge to properly monitor the trial.

● your qualifications must be documented.

● must be familiar with the investigated intervention, protocol, informed consent and any other written information to be provided to participants, standardized procedures, and good clinical practice guidelines.

It is also up to the committee to define the monitoring method: in loco, centralized or a combination of both, if justified. The logic of the chosen monitoring strategy must be documented.

| **Objective 3. Evaluate the cost-effectiveness of biperiden** |
| --- |

**Context**

Cost-effectiveness analysis is a type of economic evaluation that compares two or more treatment alternatives, in terms of associated costs and consequences. The cost-effectiveness analysis is performed only in situations in which the alternatives have already been evaluated, and the aspects of effectiveness and safety have been verified.

Thus, considering the above premise, the cost-effectiveness study will only be conducted if the results of the clinical trial have proven the effectiveness and safety of biperiden, in terms of statistical significance and clinical relevance. Thus, if carried out, the cost-effectiveness study will be designed and conducted after the period comprising the three-year period in which the project was initiated.

**Effectiveness data**

For the economic evaluation, the effectiveness data obtained from the randomized clinical trial (bottom up approach) will be used [Drummond 2006; Glick 2015].

**Cost data**

The measurement of costs will occur in three stages, specifically

(i) identification of the type of resource used by each participant (eg, daily stay in the ICU, complementary exams, medication).

(ii) quantification of resources used (resource consumption)

(iii) allocation of financial values ​​for each resource used.

Considering that the execution of this study depends on the confirmation of the effectiveness and safety of biperiden determined by the clinical trial, the cost data referring to items (ii) and (iii) will not be collected during the clinical trial, and may be collected later from the centers themselves participants or may originate from other sources, such as the average amounts paid by SUS. All sources used will be explicitly presented.

**Modeling**

The modeling will be carried out with a decision tree model, using TreeAge, R or Excel software.

**Planned main outcome**

The cost-effectiveness analysis will be based on the incremental cost-effectiveness ratio between the two interventions compared in the clinical trial.

**Time horizon**

The time horizon to be evaluated will be two years, considering the follow-up time of the clinical trial participants.

**Discount rate**

A discount rate of 5% will be used, both for the costs incurred and for the clinical effects observed in the second year of follow-up.

**Perspective**

The perspective of the cost-effectiveness analysis will be that of the SUS. This perspective will guide the resources to be considered in the analysis. Expenses incurred by other parties, such as expenses borne by the research participant, will therefore be excluded from the analysis.

The inclusion of indirect costs in the analysis, such as administrative costs, will depend on the availability of this data.

**Sensitivity analyzes**

Deterministic and probabilistic sensitivity analyzes will be performed to reduce the uncertainty associated with the model parameters.

Deterministic sensitivity analyzes will be performed with variation of discount rates and values ​​of resources obtained from different sources:

● Regarding the discount rate: the values ​​of 3% and 7% will be applied, for costs and clinical effects, in the second year of treatment;

● Regarding the assumed costs: variations of 10% will be applied upwards and downwards from the initial values ​​determined.

Probabilistic sensitivity analyzes will be performed by Monte Carlo simulations, with 1000 iterations, using the gamma distribution for costs and the normal distribution for clinical effects.

1. **DELIVERY SCHEDULE, ACTIVITIES AND MILESTONES**

In view of the above, below, the updated complete schedule of the project for execution in the current triennium (2021-2023).

All efforts will be focused on completing the study in 2023, however, if we are not successful in the patient inclusion stage, with the number of patients included below the expected for the period, an additional period may be requested in the subsequent triennium, to complete the study.

**DELIVERY SCHEDULE**

| **Timeline** | **2021** | | | | **2022** | | | | **2023** | | | |
| --- | --- | --- | --- | --- | --- | --- | --- | --- | --- | --- | --- | --- |
| **Deliveries/Activities** | **1º Sem.** | | **2º Sem.** | | **1º Sem.** | | **2º Sem.** | | **1º Sem.** | | **2º Sem.** | |
|  | **P** | **E** | **P** | **E** | **P** | **E** | **P** | **E** | **P** | **E** | **P** | **E** |
| **Delivery 1 - Planning** |  |  |  |  |  |  |  |  |  |  |  |  |
| Activity 1.1 - Selection of centers, feasibility, invitation and acceptance of participation | X |  | X |  |  |  |  |  |  |  |  |  |
| **Delivery 2 - Design of Project Flows and regulatory stage** |  |  |  |  |  |  |  |  |  |  |  |  |
| Activity 2.1 - Submission to the CEPS of the participating centers | X |  | X |  | X |  |  |  |  |  |  |  |
| Activity 2.2 - Development of the CRF / Database | X |  | X |  |  |  |  |  |  |  |  |  |
| Activity 2.3 - Term of agreement signed with the participating centers | X |  | X |  | X |  |  |  |  |  |  |  |
| **Delivery 3 - Resources for the project** |  |  |  |  |  |  |  |  |  |  |  |  |
| Activity 3.1 - Insurance contracting | X |  | X |  |  |  |  |  |  |  |  |  |
| Activity 3.2 - Acquisition of medicines | X |  | X |  | X |  |  |  |  |  |  |  |
| Activity 3.3 - Acquisition of neuropsychological tests |  |  | X |  |  |  |  |  | X |  |  |  |
| Activity 3.4 - Collection and Analysis of Laboratory Tests |  |  |  |  | X |  | X |  |  |  |  |  |
| Activity 3.5 - Storage and Transport of Medicines |  |  |  |  | X |  | X |  |  |  |  |  |
| Activity 3.6 - Hiring neuropsychological services |  |  | X |  |  |  |  |  |  |  | X |  |
| **Delivery 4 - Intervention and follow-up** |  |  |  |  |  |  |  |  |  |  |  |  |
| Activity 4.1 - Inclusion of participants |  |  |  |  | X |  | X |  |  |  |  |  |
| Activity 4.2 - Clinical follow-up |  |  |  |  | X |  | X |  | X |  | X |  |
| **Delivery 5 - Monitoring** |  |  |  |  |  |  |  |  |  |  |  |  |
| Activity 5.1 - Development of monitoring plan and materials | X |  | X |  |  |  |  |  |  |  |  |  |
| Activity 5.2 - Monitoring visits (data and documents) |  |  |  |  | X |  | X |  | X |  | X |  |
| Activity 5.3 - Meeting Investigators |  |  | X |  |  |  | X |  |  |  |  |  |
| Activity 5.4 - Monitoring of project indicators |  |  |  |  | X |  | X |  | X |  | X |  |
| **Delivery 6 - Publication of results** |  |  |  |  |  |  |  |  |  |  |  |  |
| Activity 6.1 - Sending the database to the MS and approval for publication |  |  |  |  |  |  |  |  |  |  | X |  |
| Activity 6.2 -Meeting final investigators |  |  |  |  |  |  |  |  |  |  | X |  |
| Activity 6.3 - Preparation of articles and publications |  |  |  |  | X |  |  |  |  |  | X |  |
| Activity 6.4 - Presentation at a congress |  |  |  |  |  |  |  |  |  |  | X |  |

**/**

**SCHEDULE OF EVALUATION OF THE BIPERIDENE STUDY**

| Treatment | 5 mg (1 mL total volume) of biperiden lactate. |
| --- | --- |
| Placebo | 1 mL lactate diluted in sterile water for injection. |
| Via | Intravenous route 6/6 hours |
| Treatment Period | **(rand.) D1, D2, D3, D4, D5, D6, D7, D8, D9, D10. D1 is the day of randomization on which the patient MUST receive the drug/placebo.** |
| Follow-up period 1 | **Essential follow-ups from D1: 3, 12 and 24 months**  **Follow-up visits should be scheduled at patient discharge. For these follow-up points, a variation of ± 5 days can be tolerated (due to holidays or unavailability), however, the centers will be instructed to avoid missing the correct date.** |
| Follow-up period 2 | **Patient follow-up to assess clinical evolution: 1, 6, 9 and 18 months from D1.**  **Follow-up visits should be scheduled at patient discharge. For these follow-up points, a variation of ± 15 days can be tolerated (due to holidays or unavailability), however, the centers will be instructed to avoid missing the correct date.** |

| **Deliveries** | | | |
| --- | --- | --- | --- |
|  | **Detailing** | | |
| **Delivery 1** | **Project Planning and Coordination** |  |  |
| **Delivery 2** | **Design of project flows and regulatory stage** |  |  |

| **Delivery 3** | **Resources needed for the project** |  |  |
| --- | --- | --- | --- |
| **Delivery 4** | **Inclusion of Patients** |  |  |
| **Delivery 5** | **Study monitoring** |  |  |
| **Delivery 6** | **Cost-effectiveness study** |  |  |
| **Delivery 7** | **Data analysis** |  |  |
| **Delivery 8** | **Publication of results** |  |  |

| **Identification of Landmarks** |
| --- |

| **Marco** |  |
| --- | --- |
| M.1 | Agreement with all hospitals – Delivery related 2. Contract drafts validated and signed by all participating centers and HSL |
| M.2 | Ethics Committee Approval – Delivery Related 2. Receipt of approval from the local ethics committee of all participating centers. Completion of this delivery is essential for compliance with Clinical Research legislation and initiating recruitment. |
| M.3 | Enrolled Patients – Delivery Related 5. This milestone refers to patients enrolled in the study. A statistical calculation was performed that demonstrates the sample size necessary for the results of this study to have statistical power and can be extrapolated to the population. This milestone will be reached when this sample size is reached. The sample size and its calculation are described in detail in the methodology. |
| M.4 | Final data analyzed and published – Delivery related 6. The scientific publication of the study and its last milestone, closing the last activity and delivery agreed in the present plan. Scientific publication is the most effective way of communicating the results of a research project. |

1. **ANNUAL RESULTS EVALUATION EVENTS**

Working meeting with the Ministry of Health, in December 2020, to monitor the project and evaluate what was carried out within the triennium, considering that the research results will be possible to be presented after two years of patient follow-up.

1. **SAW RISK MANAGEMENT PLAN**

Table 1 presents the mapping and description of project risks, intervention proposals to minimize them and the expected response to these interventions. Also consider the following possible risk mitigation points:

1. If the patient is still hospitalized 1 month after D1 – visit the hospital.

2. If the patient is discharged up to ± 10 before completing 1 month of D1 – perform the “1 month” assessment before the patient is discharged (to avoid missing this first month of follow-up)Contato do paciente: para evitar perda no seguimento o centro deverá coletar ao menos 3 telefones do paciente ou alguém relacionado a ele e preencher na ficha de coleta de dados eletrônico

3. For each patient, a sign must be placed on their bed (during hospitalization) indicating their participation in the study. This should also be included in the medical evolution as soon as the patient is randomized and intervention prescribed – on D1. This evolution document will be monitored

4. Team change: The center has 48 hours to inform the sponsor (HSL) about any and all team changes, so that recruitment is not affected and that new professionals are properly trained and delegated.

1. **DISCLOSURE OF PROJECT RESULTS**

It is important to emphasize that the disclosures provided herein will only be viable when the analysis of the data in the subsequent three-year period is completed. For the dissemination of project results, the following activities are planned:

● Preparation of two manuscripts:

○ A manuscript with the report of the final results of the randomized clinical trial, prepared following the CONSORT [Schulz 2010], to be submitted in a scientific journal indexed preferably in ISI-Clarivate Analytics and

○ A manuscript with the report of the cost-effectiveness study, prepared following the CHEERS [Husereau 2013], to be submitted to a scientific journal (preferably indexed in ISI-Clarivate Analytics).

● Two posters or oral presentations to be submitted to international congresses in the clinical area of ​​interest and/or methodology/pharmacoeconomics area.

1. **ADDITIONAL INFORMATION AND ATTACHMENTS**

**About scientific production and publication of results**

The delineation of the scope of scientific productions that constitute the delivery of the project agreed with the Ministry of Health, as well as the definition of the collaborators involved, must be defined by the project leadership. The leadership figure is usually exercised by the project manager, the research project coordinator or the product owner (P.O).

The scientific productions that make up deliveries agreed in the work plan must be prioritized, in relation to other types of production within the scope of the PROADI-SUS project. This understanding is important so that the supplementary productions eventually developed do not incur the situation of scientific duplication, that is, the double publication of the same results of a study. Therefore, it is recommended that the scope of planned scientific production be defined early, during the course of the project, in order to identify the windows of opportunity for supplementary scientific production.

The definition of authors' roles should be carried out in the planning phase of the study, whenever possible2. The early definition of authors' roles increases transparency in relation to the attributions of the research team, aligning individual expectations with the order of authorship of the final product. This definition is especially important for authors with a prominent role, such as first author, last author, and the corresponding author.

The selection of authors must be guided by the fulfillment of the four criteria for authorship recognition proposed by the ICMJE.

**ICMJE Authorship Criteria (2):**

1 – Substantial contributions to the conception or design of the work; or the acquisition, analysis or interpretation of data for work; AND

2 – Elaboration of the work or critical review of it regarding its important intellectual content; AND

3 – Final approval of the version to be published; AND

4 – Responsibility for all aspects of the study ensuring that issues related to the accuracy or completeness of any part of the study are properly investigated and resolved.

It must be ensured that all authors involved in scientific production have a fair opportunity to make their contributions, so that they can legitimize their authorship. Likewise, it must be ensured that everyone who has contributed to the four ICMJE criteria is included among the authors.

Regarding the meeting with the participating researchers and the presentation of results at an international congress. It is proposed in this work plan a virtual meeting with all the researchers, SCITE and ANVISA to present the progress of the study, discuss possible obstacles and scientific training of the researchers of the research centers. A meeting of researchers is also proposed to be held at the end of the triennium in December 2023 to present preliminary results and discuss studies related to the platform. The principal investigators are also expected to visit the Sírio-Libanês Hospital to present the preliminary results of the studies.

1. **REFERENCES**

Antoniuk, SA & Schwind, MR. Crisis no epilépticas en la infancia y adolescencia. MEDICINA (Buenos Aires),2013; 73.

Annegers JF, Hauser WA, Coan SP, Rocca WA. ‘A Population-Based Study of Seizures after Traumatic Brain Injuries. N England J of Med. 1998;338(1):20-4.

Aronstam, RS & Patil, P. Muscarinic Receptors: Autonomic Neurons, Encyclopedia of Neuroscience, Academic Press,2009; 1141-1149, ISBN 9780080450469, https://doi.org/10.1016/B978-008045046-9.00692-6.

Asikainen I Kaste, M., Sarna, S. Early and late posttraumatic seizures in traumatic brain injury rehabilitation patients: brain injury factors causing late seizures and influence of seizures on long-term outcome. Epilepsia. 1999;40(5):584-9.

Anvisa. MANUAL PARA NOTIFICAÇÃO DE EVENTOS ADVERSOS E MONITORAMENTO DE SEGURANÇA EM ENSAIOS CLÍNICOS - 1a. edição. 2016. Disponível em: <http://portal.anvisa.gov.br/documents/33836/2492465/Manual+para+Notifica%C3%A7%C3%A3o+de+Eventos+Adversos+e+Monitoramento+de+Seguran%C3%A7a+em+Ensaios+Cl%C3%ADnicos+-+1%C2%AA+Edi%C3%A7%C3%A3o/04a68574-8aac-43c9-b0b2-7b7cd80831c4>. Acessado em 5 de novembro de 2019.

Brady, RD., Casillas-Espinosa, PM., Agoston, DV., Bertram, EH., Kamnaksh, A., Semple, BD., & Shultz, SR. Modelling traumatic brain injury and posttraumatic epilepsy in rodents. Neurobiology of disease, 2019; *123*, 8-19.

Benassi SK, Alves JGSM, Guidoreni CG, Massant CG, Queiroz CM, Garrido-Sanabria E, Loduca RDS, Susemihl MA, Paiva WS, de Andrade AF, Teixeira MJ, Andrade JQ, Garzon E, Foresti ML, Mello LE. Two decades of research towards a potential first anti-epileptic drug. Seizure. 2021 Mar 3:S1059-1311(21)00065-0.

Bittencourt S, Ferrazoli E, Valente MF, et al. Modification of the natural progression of epileptogenesis by means of biperiden in the pilocarpine model of epilepsy. Epilepsy Research. 2017; 138:88-97.

Comissão Nacional de Ética em Pesquisa (CONEP). Resolução no. 466, de 12 de dezembro de 2012. Disponível em: <https://conselho.saude.gov.br/resolucoes/2012/Reso466.pdf>. Acessado em 25 de outubro de 2019.

D’Ambrosio R, Perucca E. Epilepsy after head injury. Current Opinion in Neurology. 2004;17(6):731-5.

da Silva AM, Vaz AR, Ribeiro I, Melo AR, Nune B, Correia M. Controversies in posttraumatic epilepsy'. Acta Neurochir Suppl (Wien).1990; 50:48-51.

de Almeida CE, de Sousa Filho JL, Dourado JC, Gontijo PA, Dellaretti MA, Costa BS. Traumatic Brain Injury Epidemiology in Brazil. World Neurosurg. 2016; 87:540-7.

DeVito NJ, Goldacre B. Catalogue of bias: publication bias. BMJ Evidence-Based Medicine. 2019; 24:53-5.

[Drummond](https://www.amazon.com.br/s/ref=dp_byline_sr_book_1?ie=UTF8&field-author=Michael+F.+Drummond&search-alias=books) MF, [Sculpher](https://www.amazon.com.br/s/ref=dp_byline_sr_book_2?ie=UTF8&field-author=Mark+J.+Sculpher&search-alias=books) MJ, [Torrance](https://www.amazon.com.br/s/ref=dp_byline_sr_book_3?ie=UTF8&field-author=George+W.+Torrance&search-alias=books) GW. Methods for the Economic Evaluation of Health Care Programmes. Oxford University Press, USA; 3a. Edição: 2006.

Englander J, Bushnik T, Duong T. Analyzing risk factors for late posttraumatic seizures: a prospective multicenter investigation. Arch Phys Med Rehabil.2003;84:365-73.

European Medicines Agency (EMA). ICH Harmonised Tripartite Guideline E6: Note for Guidance on Good Clinical Practice (PMP/ICH/135/95) London: European Medicines Agency; 2002. Disponível em: <https://www.ema.europa.eu/en/ich-e6-r2-good-clinical-practice>. Acessado em 24 de outubro de 2019.

EuroQoL Group 1990. EuroQol--a new facility for the measurement of health-related quality of life Health Policy[.](https://www.ncbi.nlm.nih.gov/pubmed/10109801) 1990;16(3):199-208.

Faul M, Coronado V. Epidemiology of traumatic brain injury. Handb Clin Neurol. 2015; 127:313.

French JA, White HS, Klitgaard H, *et al*. Development of new treatment approaches for epilepsy: Unmet needs and opportunities. Epilepsia. 2013;54(Suppl4):3-12.

Gentile JKA, Himuro HS, Rojas SSA, *et al.* Condutas no paciente com trauma cranioencefálico. Rev Bras Clin Med. São Paulo. 2011;9(1):74-82.

Glick HA, Doshi JA, Sonnad SS, Polsky D. Economic Evaluation in Clinical Trials. Oxford University Press, UK; 2a. Edição: 2015.

Gorgati C, Mello LEAM. Caracterização dos efeitos antiepileptogênicos do biperideno em ratos no modelo da pilocarpina PhD thesis, Neurology Graduate Program. Universidade Federal de São Paulo. 2009.

Hauser WA, et al Prevalence of epilepsy in Rochester, Minnesota: 1940-1980'. Epilepsia. 1991; 32:429-45.

Howick J, Chalmers I, Glasziou P, et al for the OCEBM Levels of Evidence Working Group*. “The Oxford Levels of Evidence 2”. 2011.Oxford Centre for Evidence-Based Medicine. Disponível em: <https://www.cebm.net/index.aspx?o=5653>. Acessado em 23 de outubro de 2019.

[Husereau D](https://www.ncbi.nlm.nih.gov/pubmed/?term=Husereau%20D%5BAuthor%5D&cauthor=true&cauthor_uid=23526140), [Drummond M](https://www.ncbi.nlm.nih.gov/pubmed/?term=Drummond%20M%5BAuthor%5D&cauthor=true&cauthor_uid=23526140), [Petrou S](https://www.ncbi.nlm.nih.gov/pubmed/?term=Petrou%20S%5BAuthor%5D&cauthor=true&cauthor_uid=23526140), et al. Consolidated Health Economic Evaluation Reporting Standards (CHEERS) statement. [Eur J Health Econ.](https://www.ncbi.nlm.nih.gov/pubmed/23526140) 201314(3):367-72. doi: 10.1007/s10198-013-0471-6.

Imperial College Clinical Research Governance Office. Good Clinical Practice [Web Page] 2007. Disponível em: <http://www.imperial.ac.uk/clinicalresearchoffice>.Acessado em 24 de outubro de 2019.

Kim, J.A., Boyle, EJ., Wu, AC., Cole, AJ., Staley, KJ., Zafar, S., ... & Westover, MB. Epileptiform activity in traumatic brain injury predicts post‐traumatic epilepsy. Annals of neurology, 2018; *83*(4), 858-862.

Klein P, Tyrlikova I. Prevention of epilepsy: Should we be avoiding clinical trials? Epilepsy Behav.2017;72:188-94.

Maas, AI., Menon, DK., Adelson, PD., Andelic, N., Bell, MJ., Belli, A., … & Citerio, G. Traumatic brain injury: integrated approaches to improve prevention, clinical care, and research. The Lancet Neurology, 2017; *16*(12), 987-1048.

Magalhães ALG, Souza LC, Faleiro RM , Teixeira AL, Miranda AL. Epidemiologia do traumatismo cranioencefálico no Brasil. Revista Brasileira de Neurologia 2017;53(2):15-22.

Masel BE, DeWitt DS. Traumatic brain injury: a disease process, not an event. J Neurotrauma. 2010;27(8):1529-40.

Ministério da Saúde. Secretaria de Atenção à Saúde.Diretrizes de atenção à reabilitação da pessoa com traumatismo cranioencefálico. Brasília: Ministério da Saúde, 2015.

Moran SP, Maksymetz J, Conn PJ. Targeting Muscarinic Acetylcholine Receptors for the Treatment of Psychiatric and Neurological Disorders. Trends in pharmacological sciences. 2019 Nov 8.

NCT01048138.Use of Biperiden for the Prevention of Post-traumatic Epilepsy. Disponível em: <https://clinicaltrials.gov/ct2/show/NCT01048138?term=biperideno&rank=1>. Acessado em 18 de outubro de 2019.

Okamoto OK, Janjoppi L, Bonone FM, *et al*. Whole transcriptome analysis of the hippocampus: toward a molecular portrait of epileptogenesis. BMC genomics. 2010; 11:230.

Oliveira M da S. Figuras Complexas de Rey: teste de cópia e de reprodução de memória de figuras geométricas complexas. Manual André Rey. Revisão técnica Teresinha Rey, Lucia C. F. Franco. Tradução Teresinha Rey, Lucia C. F. Franco. São Paulo: Casa do Psicólogo; 1999

Payan H, Toga M, Bérard-Badier M. The pathology of post-traumatic epilepsies. Epilepsia. 1970;11(1):81-94.

Pereira HAA, Benassi SK, Mello LE. Plastic Changes and Disease-modifying Effects of Scopolamine in the Pilocarpine Model of Epilepsy in Rats. Epilepsia.2005;46(s5):118-124.

Piccenna L, Shears G, O'Brien TJ. Management of post‐traumatic epilepsy: An evidence review over the last 5 years and future directions. Epilepsia. 2017;2(2):123-44.

Pitkänen A. Therapeutic approaches to epileptogenesis - Hope on the horizon’. Epilepsia. 2010; 51(Suppl 3):2-17.

# Pocock SJ. Clinical Trials - a practical approach. John Wiley & Sons, Chichester - New York - Brisbane - Toronto -Singapore 1983, 265 S.

Radu, BM, Osculati, AMM., Suku, E. …Fabene, PF. All muscarinic acetylcholine receptors (M1-M5) are expressed in murine brain microvascular endothelium. Sci Rep 7, 2017; 5083 (7) doi: 10.1038/s41598-017-05384-z

Rawlings DB, Crewe NM. Test-retest practice effects and test score changes of the WAIS-R in recovering traumatically brain-injured survivors. Clinical Neuropsychologist.1992;6:415-30. doi:10.1080/13854049208401868.

Raymont V, Salazar AM, Lipsky R, *et al*. Correlates of posttraumatic epilepsy 35 years following combat brain injury'. Neurology. 2010; 75:224-9.

Rey A. L’examen psychologique dans les cas d’encéphalopathie traumatic. 1941. Arch Psychol 28:286–340.

Rey A. L'examen Clinique En Psychology. Paris: Press Universitaire de France, 1964.

Salazar AM, Grafman J. Epilepsy after penetrating head injury. I. Clinical correlates: a report of the Vietnam Head Injury Study. Neurology.1985;35(10):1406-14.

Salazar AM, Grafman J. Post-traumatic epilepsy: clinical clues to pathogenesis and paths to prevention. Handb Clin Neurol. 2015; 128:525-38.

Schulz KF, Altman DG, Moher D for the CONSORT Group. CONSORT 2010 Statement: updated guidelines for reporting parallel group randomised trials. BMJ. 2010; 340:c332. Disponível em: <https://www.bmj.com/content/340/bmj.c332>. Acessado em 18 de outubro de 2019. doi: <https://doi.org/10.1136/bmj.c332>.

Santos, MF, dos Santos Silva, TDC, de Carvalho, FR, Barbosa, RL, dos Santos, LH, & de Matos Junior, EM. TCE em UTI: Epidemiologia, tratamento e mortalidade no maranhão, brasil. Revista Brasileira de Neurologia e Psiquiatria. 2019: *23*(1).

Sedo MA. Test de los cinco dígitos [Five digit test]. Madrid: TEA Ediciones; 2005

Stroop, J.R. (1935). Studies of interference in serial verbal reaction. Journal of Experimental Psychology, 18, 643-662.

Temkin NR, Dikmen SS, Wilensky AJ, Keihm J, Chabal S, Winn HR. A Randomized, Double-Blind Study of Phenytoin for the Prevention of Post-Traumatic Seizures. N Engl J Med. 1990;323(8):497-502.

Temkin NR. Antiepileptogenesis and Seizure Prevention Trials with Antiepileptic Drugs: Meta-Analysis of Controlled Trials. Epilepsia. 2001;42(4):515-24.

Temkin NR. Risk Factors for Posttraumatic Seizures in Adults Epilepsia. 2003;44(Suppl10):18-20.

Verellen RM, Cavazos JE. Post-traumatic epilepsy: an overview. Therapy. 2010;7(5):527–31. doi:10.2217/THY.10.57

# [von Steinbüchel N](https://www.ncbi.nlm.nih.gov/pubmed/?term=von%20Steinb%C3%BCchel%20N%5BAuthor%5D&cauthor=true&cauthor_uid=20210602), [Wilson L](https://www.ncbi.nlm.nih.gov/pubmed/?term=Wilson%20L%5BAuthor%5D&cauthor=true&cauthor_uid=20210602), [Gibbons H](https://www.ncbi.nlm.nih.gov/pubmed/?term=Gibbons%20H%5BAuthor%5D&cauthor=true&cauthor_uid=20210602), *et al* for [QOLIBRI Task Force](https://www.ncbi.nlm.nih.gov/pubmed/?term=QOLIBRI%20Task%20Force%5BCorporate%20Author%5D).Quality of Life after Brain Injury (QOLIBRI): scale validity and correlates of quality of life.[J Neurotrauma.](https://www.ncbi.nlm.nih.gov/pubmed/20210602) 2010;27(7):1157-65. doi: 10.1089/neu.2009.1077.

Wechsler D. A standardized memory scale for clinical use. Journal of Psychology. 1945; 19:87-95. doi:10.1080/ 00223980.1945.9917223.

Wechsler D. (2008). Wechsler adult intelligence scale–Fourth Edition (WAIS–IV). San Antonio, TX: Pearson

World Medical Association. Declaration of Helsinki. Ethical principles for medical research involving human subjects. [Internet]. 59th WMA General Assembly, Seoul, oct. 2008. Disponível em: <http://www.wma.net/en/30publications/10policies/b3/17c.pdf>. Acessado em 23 de outubro de 2019.

Yablon SA. Posttraumatic seizures. Archives of physical medicine and rehabilitation.1993; 74(9):983-1001.
