# Supplementary material for: Biperiden for prevention of post-traumatic epilepsy: A protocol of a double-blinded placebo-controlled randomized clinical trial (BIPERIDEN trial)
Source: PLoS One. 2022 Sep 9;17(9):e0273584. doi: 10.1371/journal.pone.0273584 (PMC9462738; doi:10.1371/journal.pone.0273584)
Supplement: S2 File — (DOCX) [file pone.0273584.s003.docx]

**Hospital Sírio – Libanês**

Instituto de Ensino e Pesquisa

Escritório de Responsabilidade Social – PROADI SUS

**Biperideno para prevenção de epilepsia em pacientes com traumatismo cranioencefálico.**

**Pesquisadores Responsáveis:** Eliana Garzon e Luiz Eugênio Mello

**Equipe de Pesquisa:** Renata Mattos, Rachel Rieira, Maíra Foresti, Carla Pinheiro, Ana Cecília Alcantara, Débora Patricio e Mariane Luz

**São Paulo, 2021**

**Controle de alterações**

| **Data de Alterações** | **Resumo das Alterações em destaque negrito e italico** |
| --- | --- |
| **Outubro/2021** | **Equipe de Pesquisa:** Renata Mattos, Rachel Rieira, Maíra Foresti, Carla Pinheiro, Ana Cecília Alcantara, ***Débora Patricio*** ***e Mariane Luz*** |
| Justificativa: atualização dos membros da equipe. | |
| **Outubro/2021** | ***Critérios de exclusão: inclusão de itens***   - ***Participantes vulneráveis, sem documentação e sem endereço fixo e/ou contato de familiares;*** - ***Pacientes sem documento e com idade duvidosa;*** |
| Justificativa: adicionado nos critérios de exclusão do participante de pesquisa pacientes com o perfil de vulnerabilidade e sem documento com idade duvidadosa, pois pelo perfil do participante de pesquisa deste protocolo há diversas situações de entrada do paciente no hospital de urgência e emergência. Assim para mitigar perdas de participantes do estudo que prevê seguimento clínico por 24 meses, adicionamos este perfil de pacientes no critério de exclusão para facilitar o entendimento de perfil dos pacientes para o protocolo de pesquisa.  Outro ponto de exclusão é referente aos pacientes sem documentos e com idade adulta duvidosa, para mitigar possíveis desvios de protocolo sugerimos a exclusão do participante no qual esta sem documento na entrada do hopsital e há duvidas sobre a idade do mesmo. | |
| **Outubro/2021** | ***Critérios de exclusão: exclusão de item***   - ***História familiar de epilepsia em parentes de primeiro grau;*** |
| Justificativa: retirado dos critérios de exclusão do participante de pesquisa pacientes com o histórico familiar de epilepsia em parentes de primeiro grau, pois este critério não é impeditivo para participação do indivíduo no estudo. É inclusive relevante para identificar possível influência genética no desenvolvimento de epilepsia pós-traumática em sujeitos com histórico de familiares próximos com epilepsia. | |
| **Outubro/2021** | ***Exclusão do uso do Log de medicação para controlar a adesão ao protocolo o centro deverá fazer um log de medicação/placebo prescrita e administrada*** |
| Justificativa: Não vamos utilizar log de medicação de controle manual, pois o centro coordenador esta desenvolvendo uma ficha eletrônica (REDCap) no qual a equipe de pesquisa terá acesso com todas as datas previstas para a equipe de enfermagem administrar o medicamento e uma ficha eletrônica (REDCap) para o cadastro da real data e hora administrada e este será o dado oficial do projeto. | |
| **Outubro/2021** | Dentro do período de 12 horas após o TCE, os participantes do grupo placebo receberão 1mL ***do mesmo excipiente utilizado na produção do medicamento ativo***, onde será diluído em 10 mL de soro fisiológico 0,9% e aplicado por via intravenosa lentamente. |
| Justificativa: Atualização da composição do placebo que será fornecido pela empresa Cristália, no protocolo inicial o placebo composto por **1 mL do soro fisiológico 0,9%,** seriam fornecidos por outra empresa. Contudo ao conseguir parceria com a indústria farmacêutica cristália seguiremos com o padrão produzido por eles. | |
| **Maio/2021** | **Equipe de Pesquisa: *Renata Mattos,*** Rachel Rieira, ~~Flávia Regina Bueno~~, Maíra Foresti, ***Carla Pinheiro, Ana Cecília Alcantara*** |
| Justificativa: adição membros da equipe. | |
| **Maio/2021** | Após a inclusão, as seguintes informações serão coletadas em ***formulário padronizado em plataforma eletrônica amplamente utilizada chamada RedCap*** |
| Justificativa: adição do uso de plataforma eletrônica para melhor acompanhamento dos dados, rastreabilidade e segurança. | |
| **Maio/2021** | Assim, seriam necessários ***156 participantes em cada grupo, sendo planejada a inclusão de 312 participantes.*** |
| Justificativa: Novo calculo amostral para o estudo, adição de participantes da pesquisa para robustez dos resultados. | |
| **Maio/2021** | Em caso de alta hospitalar no período inferior a 10 dias, ***o participante poderá ser incluído na análise do estudo (por intenção de tratar) caso ele tenha utilizado 30 doses ou mais do Biperideno. Caso o participante não tenha atingido essas 30 doses mínimas será realizado uma notificação de desvio de protocolo.*** |
| Justificativa: retirado a possibilidade de continuar tratamento em casa com comprimidos, será realizado a intervenção somente em ambiente hospitalar via endovenosa. | |
| **Maio/2021** | No ensaio clínico proposto, a dose administrada para o ‘grupo biperideno’ será de uma ampola (5 mg de lactato de biperideno), diluída em soro fisiológico e administrada durante 30 a 60 minutos, a cada 6 horas, ***durante 10 dias completando 40 doses administradas.*** A dose total diária será então de 20mg de lactato de biperideno, dentro do limite apresentado em bula. |
| Justificativa: retirado a possibilidade de continuar tratamento em casa com comprimidos, será realizado a intervenção somente em ambiente hospitalar via endovenosa. | |
| **Maio/2021** | Em caso de alta hospitalar no período inferior a 10 dias, impossibilitando a conclusão do protocolo no ambiente hospitalar, ***o participante poderá ser incluído na análise do estudo (por intenção de tratar) caso ele tenha utilizado 30 doses ou mais do Biperideno. Caso o participante não tenha atingido essas 30 doses mínimas será realizado uma notificação de desvio de protocolo.*** |
| Justificativa: retirado a possibilidade de continuar tratamento em casa com comprimidos, será realizado a intervenção somente em ambiente hospitalar via endovenosa. | |
| **Maio/2021** | Estima-se a participação de ***nove centros e é esperado que sejam incluídos em torno de 3 a 5 participantes por centro/mês, o que implica em 14 a 18 meses para a inclusão dos 312 participantes.*** O tempo total estimado para a duração do estudo (fase de intervenção de 10 dias seguida de fase de acompanhamento de 24 meses) é de 38 a 42 meses, ou seja, avançando para o triênio seguinte ao triênio de início do projeto. |
| Justificativa: Novo calculo amostral para o estudo, adição de participantes da pesquisa para robustez dos resultados. | |
| **Maio/2021** | O participante será excluído do estudo em casos de gestação, manifestação de desejo de descontinuar sua participação, ocorrência de eventos adversos graves e que coloquem em risco a vida do participante (a critério do investigador em discussão com o comitê de monitoramento); ***caso tenha havido erro de screening ao incluir pacientes com histório de epilepsia/glaucoma ou qualquer outra condição de saúde prévia ao TCE e prevista nos critérios de exclusão do estudo (quando este achado não puder ocorrer no momento do recrutamento); qualquer outra condição que, na opinião do investigador, seja vantajoso para o paciente o não cumprimento dos procedimentos especificados neste protocolo.***  ***Os participantes que não completaram as doses mínimas necessárias (30 doses), por motivo de alta hospitalar, óbito, ou qualquer outra intercorrência no qual impossibilite a finalização das administrações do medicamento serão considerados desvios de protocolo.*** |
| Justificativa: adição de informações do fluxo do paciente em casos de intercorrências do estudo como de falha de screening e desvio de protocolo. | |
| **Maio/2021** | ***A central de randomização disponibilizará cada número randômico via plataforma digital no qual o centro participante terá acesso disponível.*** Assim que um participante preencher os critérios de elegibilidade e for incluído no estudo, ***um membro da equipe do centro coordenador delegado acessará a plataforma digital e terá acesso ao numero do frasco do medicamento a ser administrado.*** A sequência numérica deverá ser seguida rigorosamente e este processo será verificado nas visitas de monitoria. ***A plataforma digital deverá ser acessada apenas imediatamente antes de o paciente receber a primeira dose da intervenção. O membro da equipe do centro coordenador delegado se comunicará com a farmácia local para dizer-lhe o número do frasco-ampola que deverá ser dispensado.*** ***A farmácia local entregará à enfermeira as ampolas identificadas com os números randômicos da plataforma digital.*** Ambos não saberão qual o conteúdo do frasco. |
| Justificativa: atualização do processo de randomização para melhoria e garantia da qualidade do protocolo de pesquisa. | |
| **Maio/2021** | Diante do ora exposto, a Sociedade encaminha, ***abaixo, o cronograma completo atualizado do projeto para execução no triênio vigente (2020-2023).***  Todos os esforços serão concentrados para finalização do estudo em 2023, entretanto se não obtivermos sucesso na etapa de inclusão de pacientes, com número de pacientes incluídos abaixo do esperado para o período, poderá ser solicitado período adicional no triênio subsequente, para finalização do estudo. |
| Justificativa: atualização do cronograma para melhoria e garantia da qualidade do protocolo de pesquisa. | |

**Protocolo do Estudo: Biperideno para prevenção de epilepsia em pacientes com traumatismo cranioencefálico.**

1. **JUSTIFICATIVA**

*Traumatismo cranioencefálico*

Definido como alteração da função cerebral ou evidência de patologia cerebral causada por uma força externa, o traumatismo cranioencefálico (TCE) é responsável por 1% de todas as mortes em adultos no mundo [Gentile 2011], o que corresponde a 50 milhões de pessoas ao redor do mundo todo ano, sendo a principal causa de mortalidade e comorbidade em adultos em todos os países, inclúsive no Brasil [Maas 2017; Ministério da Saúde 2015; Pereira 2006].

Dados do DATASUS mostram que cerca de 125.000 pessoas/ano recebem atendimento hospitalar devido ao TCE, o que corresponde a uma frequência de 65,7 casos / 100.000 habitantes [Magalhães 2017]. Como preocupação adicional, a taxa de mortalidade hospitalar para estes casos chega a 7,7%, ou seja, 5,1 mortes /100.000 casos / ano [Magalhães 2017]. Os acidentes de trânsito representam a principal causa de TCE, totalizando 50% dos casos, seguidos por quedas e violência urbana [de Almeida 2016].

Estudos mostram que apesar do tamanho do impacto do TCE, ainda são escassas evidências científicas fortes para embasar diretrizes e recomendações de tratamento, com ensaios clínicos falhando em mostrar real eficácia de tratamento, apesar de resultados promissores [Maas 2017].

Com relação à morbidade associada ao TCE, as complicações neurológicas incluem lesões cerebrais primárias (resultado direto do trauma) e secundárias (iniciadas após o momento do trauma, derivada dos danos primários) [Faul 2015; Masel 2010]. Sobreviventes ao TCE, frequentemente experenciam traumas físicos, pscicológicos, emocionais e cognitivos, o que pode causar uma redução importante na qualidade de vida e funcionalidade do indíviduo e traz consigo um alto impacto socioeconômico [Maas 2017].

*Epilepsia Pós-traumática*

A epilepsia pós-traumática *(*EPT) é uma complicação neurológica que ocorre em até 20% dos pacientes, com o risco aumentando de acordo com a severidade do TCE, intervenção cirurgica, intervalo de tempo desde o TCE, sendo que esse percentual sobe para 53% nos casos mais graves com lesões penetrantes [Annegers 1998; Asikainen 1999; Englander 2003; Hauser 1991; Kim 2018; Raymont 2010; Salazar 2015; Temkin 1990].

Após um TCE podem surgir crises de natureza epiléptica, precoces (em até uma semana pós-TCE) mas que não caracterizam EPT. O consenso atual é que as crises que caracterizam a EPT são as que ocorrem após a primeira semana. As crises precoces podem ser causadas por efeitos agudos do trauma, como hemorragias e edema cerebral (e não necessariamente caracterizam epilepsia), enquanto as tardias parecem depender de mecanismos de reorganização sináptica [Payan 1970; Yablon 1993]

A maioria das pessoas que desenvolve epilepsia secundária ao trauma craniano manifesta as crises epilépticas nos dois primeiros anos após o trauma ou lesão [da Silva 1990]. Assim como ocorre em outros tipos de epilepsia, as causas que ligam o TCE à EPT ainda não foram totalmente elucidadas e ainda não é possível evitar o processo da epileptogênese. Fomos incapazes de encontrar qualquer evidência forte de um tratamento específico para previnir ou reverter as crises epilépticas após TCE [Brady 2019].

*Tratamento do TCE e prevenção da EPT*

A conduta terapêutica indicada para o TCE pode envolver vários medicamentos e/ou procedimentos cirúrgicos, para cuidados dos danos primários e secundários e depende em essência da extensão da lesão e de quais áreas foram acometidas. Avanços terapêuticos envolvem medicamentos com alvos nos mecanismos dos danos secundários, incluindo bloqueadores de canais de cálcio, corticoesteroides, inibidores de aminoácidos excitatórios, antagonistas de receptor de N-metil D-aspartato (NMDA), “*scavengers*” de radicais livres, sulfato de magnésio, fatores de crescimento [Salazar 1985]. Ensaios clínicos em desenvolvimento utilizando diversas abordagens apresentam grande potencial terapêutico para TCE como eritropoetina, estatinas, células de medula óssea, progesterona.

Ensaios clínicos recentes têm tido como foco estratégias neuroprotetoras, a fim de prevenir e/ou reduzir o dano cerebral secundário ao TCE [Temkin 1990]. No entanto, nenhuma dessas intervenções parece influenciar a ocorrência de EPT [French 2013; Klein 2017; Piccena 2017; Pitkänen 2010; Temkin 2001; Temkin 2003]. Substâncias anticonvulsivantes são indicadas para o controle das crises epilépticas que eventualmente ocorram de forma aguda, mas sua administração não evita a evolução para epilepsia [D’Ambrosio 2004].

Nesse contexto, não existem medicamentos neuroprotetores com indicação de evitar que os processos epileptogênicos se estabeleçam após um dano cerebral, seja ele traumático, isquêmico, ou de outra natureza [Brady 2019; Temkin 2001]. Buscando resolver esta lacuna, estudos com modelos animais identificaram que medicamentos que alteram os processos de plasticidade neuronal, se administrados sob certas condições, tem o potencial de modificar o curso natural da EPT [Bittencourt 2017].

Especificamente com relação as condições referidas no estudo de Bittencourt, et al., [2017], encontra-se: a) a janela terapêutica, isto é o intervalo e tempo entre o evento lesivo (TCE) e a administração da primeira dose de biperideno (indicada em nosso projeto como devendo ser igual ou menor as 12 h); b) a duração da modulação do processo de plasticidade neuronal, isto é a duração do tratamento com biperideno (indicada em nosso protocolo como devendo ser de 10 dias); c) a dose de biperideno, de 5 mg a cada 6 horas. Evidências em estudos de fase pré-clínica indicaram que estas três condições acima devem ser atendidas para que o biperideno demonstre uma efetividade como agente capaz de demonstrar uma ação de modificação do curso natural da doença [Bittencourt 2017].

Em estudos experimentais utilizando modelos de epilepsia, o biperideno, um anticolinérgico de uso clínico para Parkinson, mostrou ter ação na plasticidade neuronal, reduzindo a incidência e a intensidade de crises epilépticas espontâneas e retardando o aparecimento das mesmas [Bittencourt 2017; Gorgati 2009].

Até o momento, foi realizado um estudo piloto, com pequeno tamanho amostral, para estimar a segurança e os efeitos do biperideno neste cenário. Este estudo já foi encerrado, e seus resultados foram publicados recentemente (Benassi et al., 2021). Além disso, vem sendo conduzido um ensaio clínico (NCT01048138), ambos do mesmo investigador (Dr. Luiz Mello).

Foi realizado o estudo piloto de segurança com 8 pacientes no Hospital São Paulo. Esse teste realizado entre 2005 e 2007 foi aprovado pelo CEP do Hospital São Paulo (560-05) e consistiu na administração de 5 mg de biperideno (ou placebo) a cada 6 horas e por 10 dias. Esse estudo (Benassi et al., 2021) indicou a segurança do biperideno nas condições do estudo sendo proposto pelo PROADI-SUS. As reações adversas que incluíram náuseas, vertigem e perda transitória de memória, foram todas transitórias (apenas na vigência da administração da medicação).

Esses 8 pacientes receberam tratamento ou controle placebo e foram acompanhados cronicamente. Nos pacientes que receberam placebo ocorreram de uma a duas crises, ou até mesmo óbito, enquanto que nos tratados com biperideno, nenhum foi a óbito, nenhum desenvolveu crises epilépticas francas e apenas um desenvolveu anormalidades nos registros de EEG. Este primeiro estudo não era, no entanto, destinado a avaliar eficácia, mas sim a segurança do biperideno na dose que injetamos e na condição após TCE. Observamos apenas alguns efeitos adversos moderados que poderiam estar associados ao uso de biperideno durante o protocolo experimental. Vômitos e vertigem ocorreram separadamente em dois pacientes, porém isso não difere dos efeitos esperados já relacionados a este medicamento. Um paciente apresentou perda de memória leve e transitória durante o tratamento, o que pode ser devido ao traumatismo craniano ou sugerir uma consequência do biperideno.

Da mesma forma outro paciente, que apresentou vertigem, também teve concussão no labirinto no exame de imagem. Curiosamente, nenhum efeito colateral que poderia potencialmente estar associado ao uso de biperideno foi observado em um dos pacientes. Portanto, os resultados deste primeiro estudo confirmam que a injeção intravenosa de biperideno também pode ser usada com segurança após o TCE.

O ensaio clínico (NCT01048138) tem foco nos mecanismos de ação e marcadores moleculares e tem como desfechos primários a frequência de convulsões e a presença de descarga epiléptica em eletroencefalograma durante os 10 primeiros dias após TCE e nos subsequentes 1, 3, 6, 9, 12, 18 e 24 meses. Este estudo é realizado em único centro (Hospital das Clínicas da Faculdade de Medicina da USP) e ainda não há resultados definitivos ou publicados, uma vez que o estudo está em fase final de execução e os dados permanecem fechados em relação aos grupos experimentais (placebo x tratamento, estudo duplo cego), cuja etapa foi comprometida pela pandemia do novo coronavírus.

Contudo, como resultados preliminares até o mês de abril de 2020 o estudo teve no total 122 pacientes incluídos e randomizados., que atendessem aos critérios de inclusão (em geral que tivessem quadro de TCE agudo, moderado a grave, idade entre 18 e 75 anos de idade e de ambos os sexos). Deste total, até o momento foram “excluídos” da amostra 23 pacientes, os quais apresentaram algum motivo que atendesse aos critérios de exclusão, não confirmaram os critérios de inclusão ou foram descontinuados por opção da equipe médica e/ou não aderência ao protocolo. Outros 12 pacientes encontram-se em status de “dúvida” em relação a permanência em seguimento no estudo por diferentes motivos, tais como: sem contato telefônico, transferido de hospital, mudou de estado, entre outros. Nestes casos a equipe está trabalhando para conseguir que esses pacientes retomem o seguimento mas ainda não foi concretizado.

Complementarmente, outros 22 pacientes foram a óbito e 65 pacientes (53% da amostra) permanecem em seguimento. Dos pacientes em seguimento, 13 completaram 24 meses de estudo, sendo que destes, 5 realizaram o retorno de avaliação final. Como já mencionado, a pandemia do novo coronavírus comprometeu o andamento do estudo e assim que autorizado, os retornos de avaliação dos pacientes serão retomados, mesmo com atrasos nos tempos previamente estipulados. Com relação a presença de crises epilépticas, que indicam o desenvolvimento de epilepsia pós-traumática, houve confirmação de crise clínica em 4 pacientes, Dois casos apresentam relatos sugestivos de crises mas ainda precisam de confirmação. Outros 5 casos foram destacados por apresentarem episódios que poderiam também estar relacionados a crise, mas por enquanto são menos evidentes.

O estudo NCT01048138 em questão ainda não teve os resultados publicados, contudo, sua metodologia servirá de base para a metodologia do presente projeto proposto ao MS pelo PROADI-SUS. É possível que tenhamos mais informações deste estudo no segundo semestre de 2020.

Cabe salientar que não haverá duplo financiamento e que os pacientes recrutados com recursos do PROADI-SUS não farão parte do outro estudo em andamento.

O presente projeto proposto tem foco na eficácia clínica e segurança e tem como defechos primários a proporção de participantes que vierem a desenvolver EPT e/ ou eventos adversos graves no período entre 7 dias e 24 meses após TCE, alem de ser um estudo multicêntrico, de possuir um tamanho amostral superior e de considerar um estudo de custo-efetividade ao final. Mais informações sobre os defechos deste projeto proposto poderão ser encontradas sob o item **“desfechos”.**

Adicionalmente, o Trauma cranioencefálico (TCE) é um evento com repercussões graves para o individuo e para a sociedade. A incidência de epilepsia pós-traumática (EPT), que pode chegar a acometer quase 50% dos indivíduos após um TCE, é uma das mais claras dessas repercussões. Infelizmente não há no mundo, qualquer tratamento que tenha se mostrado efetivo em reduzir EPT. Nosso projeto tem o potencial de abrir uma janela que representaria uma oportunidade não apenas para o sistema de saúde no Brasil, mas no mundo. Trata-se de uma abordagem com grande potencial de inovação desenvolvida por cientistas brasileiros com liderança e reconhecimento internacional. O aporte de recursos pelo PROADI-SUS vão focar exclusivamente em eficácia clínica e custo efetividade permitirá a inclusão de um número suficiente de pacientes e assim deve ser mais conclusivo.

Considerando a morbidade associada a EPT, seu impacto na qualidade de vida dos pacientes e de seus familiares, as consequências para a sociedade e para o sistema de saúde, bem como os custos associados ao seu tratamento, é fundamental avaliar, por meio de um ensaio clínico randomizado bem desenhado e bem conduzido, os benefícios e riscos de uma intervenção para preveni-la. Adicionalmente, caso sejam comprovadas a efetividade e a segurança da intervenção, a avaliação de sua custo-efetividade torna-se fundamental para sua implementação na prática clínica e para sua incorporação no âmbito do Sistema Único de Saúde.

1. **OBJETIVOS**
2. **Objetivo Geral**

Avaliar os efeitos (benefícios e riscos) e a custo-efetividade do uso de biperideno para prevenção de epilepsia pós-traumática (EPT) em pacientes com traumatismo cranioencefálico (TCE) e hemorragia intraparenquimatosa e/ou contusão confirmada.

1. **Objetivos Específicos**
   1. Avaliar a efetividade do biperideno para prevenção de EPT.
   2. Avaliar a segurança do biperideno para prevenção de EPT.
   3. Avaliar a custo-efetividade do biperideno para prevenção de EPT.
2. **MÉTODOS**

Considerando os objetivos específicos estabelecidos, estão previstas as atividades abaixo descritas, detalhadas em relação aos aspectos metodológicos e operacionais.

| **Objetivos 1 e 2. Avaliar a efetividade e a segurança do biperideno após traumatismo cranioencefálico** |
| --- |

**Contexto**

Ensaios clínicos randomizados são considerados os estudos epidemiológicos primários mais apropriados e confiáveis para avaliar intervenções de tratamento ou prevenção em saúde [Howick 2011]. Esta confiança se deve principalmente às características destes estudos que visam minimizar o risco de viés. Deste modo, este foi o desenho de estudo escolhido para responder a pergunta de interesse do projeto e que pode ser estruturada, utilizando o acrônimo PICO, da seguinte forma:

P (população) = indivíduos com TCE agudo moderado a grave, com hemorragia intraparenquimatosa confirmada.

I (intervenção) = biperideno

C (comparador) = placebo

O (*outcomes*, desfechos) = desfechos de efetividade e segurança (detalhados adiante)

**Desenho**

Ensaio clínico randomizado multicêntrico, duplo-cego, controlado por placebo, classificado como estudo clínico de fase 3.

O planejamento do estudo seguirá as recomendações do *Consolidated Standards of Reporting Trials* (CONSORT Statement) [Schulz 2010], que apesar de ter sido desenvolvido para orientar o relato ou a publicação, tem sido amplamente utilizado também para orientar o planejamento de um ensaio clínico randomizado.

O estudo será planejado e desenvolvido com o devido rigor a fim de minimizar a ocorrência dos viéses catalogados na literatura até o momento [de Vito 2019].

O relato dos resultados do estudo seguirá o *Consolidated Standards of Reporting Trials* (CONSORT Statement) [Schulz 2010].

**Local**

O estudo será planejado no Hospital Sírio-Libanês e desenvolvido em diversos hospitais, de acordo com disponibilidade local de recursos necessários para a implantação do protocolo de pesquisa e interesse na participação. Os centros participantes devem ter acesso às seguintes estruturas e recursos:

- Unidade de atendimento para paciente com TCE;
- Unidade de diagnóstico por imagem com disponibilidade de tomografia ou ressonância nas 24 horas do dia;
- Serviço de eletroencefalograma
- Capacidade de realizar avaliação neuropsicológica.

No decorrer do estudo, caso haja necessidade de recrutamento de mais pacientes, novos centros poderão ser incluídos. Para garantir que os centros participantes possam realizar o estudo, um questionário para avaliar sua capacidade de conduzi-lo (questionário de *feasibility*) deverá ser preenchido pela equipe de pesquisa de cada centro.

**Aspectos éticos e de registro**

O planejamento do protocolo e a execução do estudo seguirão as seguintes recomendações e diretrizes para estudos de intervenção em saúde:

- Declaração de Helsinki [World Medical Association 2008];
- *Good Clinical Practices* [Imperial College 2007]
- *International Conference for Harmonisation Of Technical Requirements for Registration of Pharmaceuticals for Human Use* (ICH 6) [EMA 2002]
- Resolução 466/12 da Comissão Nacional de Ética em Pesquisa (CONEP) [CONEP 2012].

O protocolo do estudo foi registrado na base de registros de ensaios clínicos ClinicalTrials.gov (NCT04945213), após a aprovação no Comitê de Ética em Pesquisa (CEP) do Hospital Sírio-Libanês e corroboração da aprovação do CEP de cada um dos hospitais participantes.

Considerando a natureza da lesão cerebral e os critérios de inclusão no estudo de presença de rebaixamento do nível de consciência, há a possibilidade que os pacientes não estejam aptos a fornecer o consentimento de participação no estudo e, portanto, o consentimento informado por escrito será obtido de representantes legalmente autorizados.

Considerando também que a necessidade do consentimento para administração das primeiras doses implicaria no atraso das doses iniciais e na inviabilidade de inclusão do participante, o CEP local do Hospital Sírio-Libanês (centro coordenador) foi consultado e aprovou este protocolo, onde pacientes inconscientes e desacompanhados de um responsável possam ainda receber as doses do medicamento nas primeiras 48horas, antes do fornecimento do consentimento por seu responsável. Nestes casos, as doses subsequentes somente devem ser aplicadas após o consentimento por parte do paciente, caso o mesmo seja considerado apto, ou de seu responsável.

Caso a possibilidade acima descrita não seja viável perante aos CEPs locais de cada centro participante, os indivíduos que preencherem os critérios de elegibilidade, mas para os quais não seja possível a obtenção do consentimento em participar (pelo responsável legal autorizado, se alteração do nível de consciência), não serão incluídos no estudo.

Deve-se observar que o biperideno é amplamente utilizado para outras indicações, sendo considerado um tratamento seguro, com baixa frequência de eventos adversos associados ao seu uso.

**Participantes**

*Métodos de seleção*

Os participantes todos serão aqueles atendidos no pronto-socorro dos centros participantes para tratamento e que preencherem os critérios de elegibilidade.

*Critérios inclusão*

- Consentimento em participar fornecido pelo responsável, que deverá assinar e datar o TCLE após ser orientado sobre o estudo pelo investigador principal ou responsável, ler e concordar com a carta de informação;
- Idade entre 18 e 75 anos de idade;
- Ambos os sexos;
- Diagnóstico de TCE agudo moderado a grave;
- Escala de Coma de Glasgow (GCS) maior que 6 a 12 na admissão hospitalar;
- Presença de hemorragia intraparenquimatosa aguda e/ou contusão confirmada por ressonância magnética (RM) e/ou tomografia computadorizada (TC).

*Critérios de exclusão*

- Participantes vulneráveis, em situação de rua, sem documentação e sem endereço fixo e/ou contato de familiares;
- Pacientes sem documento e com idade duvidosa (18-75 anos) na admissão hospitalar;
- Estar em uso de biperideno no período de ocorrência do TCE;
- Histórico de epilepsia (confirmado por prontuário e/ou por uso de medicamento específico e/ou referido pelo responsável legal);
- Histórico de convulsões ou uso de medicação antiepiléptica;
- História de lesões perinatais, meningite e/ou encefalite (ou outro fator de risco comprovado ou provável para epilepsia);
- História de neoplasia, doenças neurodegenerativas; história de acidente vascular encefálico (AVE), disfunção cognitiva, hiperplasia prostática benigna, bloqueio atrioventricular ou qualquer outra arritmia cardíaca, ou glaucoma;
- Gestação;
- Participação atual em outro ensaio clínico;
- Portadores de arritmias cardíacas ou glaucoma, pelo risco aumentado de desenvolver reações adversas.

Para garantir a adequação aos critérios de elegibilidade, antes da primeira dose do medicamento, serão realizados exames de Ressonancia Magnética e/ou Tomografia Computadorizada para confirmação do quadro de hemorragia intraparenquimatosa aguda. Será solicitado eletrocardiograma para avaliar a presença de arritmia.

Após a inclusão, as seguintes informações serão coletadas em formulário padronizado em plataforma eletrônica amplamente utilizada chamada RedCap e armazenadas posteriormente com manutenção do anonimato:

- variáveis demográficas e clínicas (idade, perfil de comorbidades, inventário de medicamentos de uso prévio e hábitos, principalmente alcoolismo).
- dados relacionados ao trauma (mecanismo, gravidade, tipos de lesões).
- dados de evolução clínica (crises sintomáticas, necessidade de intervenção cirúrgica e complicações relacionadas aos cuidados hospitalares).

*Cálculo do tamanho da amostra*

Para o cálculo amostral foi utilizada a fórmula de Pocock [Pocock 1993] para desfechos dicotômicos e considerando as seguintes premissas:

- Desfecho: proporção de participantes apresentando EPT após dois anos.
- Proporção esperada do desfecho primário no grupo controle: 25% [Annegers 1998; Asikainen 1999; Englander 2003; Hauser 1991; Raymont 2010; Salazar 2015; Temkin 1990].
- Proporção esperada do desfecho primário no grupo intervenção: 10%
- Erro tipo 1 (alfa): 0,05
- Erro tipo 2 (beta): 0,10
- Proporção esperada da perda: 20%

Assim, seriam necessários 156 participantes em cada grupo, sendo planejada a inclusão de 312 participantes.

**Intervenção**

*Grupo biperideno*

Dentro do período de 12 horas após o TCE, os participantes do grupo biperideno receberão a dose de 5 mg (1 ml de volume total) de lactato de biperideno (Cinetol, Cristália, Brasil), diluído em 10 mL de soro fisiológico 0,9% e aplicado por via intravenosa lentamente. O tratamento será repetido a cada 6 horas durante 10 dias consecutivos.

Em caso de alta hospitalar no período inferior a 10 dias, o participante poderá ser incluído na análise do estudo (por intenção de tratar) caso ele tenha utilizado 30 doses ou mais do Biperideno. Caso o participante não tenha atingido essas 30 doses mínimas será realizado uma notificação de desvio de protocolo.

No ensaio clínico proposto, a dose administrada para o ‘grupo biperideno’ será de uma ampola (5 mg de lactato de biperideno), diluída em soro fisiológico e administrada durante 30 a 60 minutos, a cada 6 horas, durante 10 dias completando 40 doses administradas. A dose total diária será então de 20mg de lactato de biperideno, dentro do limite apresentado em bula.

Como informação adicional sobre a segurança, a dose de 20 mg/dia está sendo utilizada no ensaio clinico de fase 3 em andamento (aprovado pelo CEP do Hospital das Clínicas da Faculdade de Medicina, Universidade de São Paulo), que já incluiu 93 participantes. Análise parcial dos resultados (ainda não abertos) identificou 22 mortes no total (risco de morte= 23%). Dados da literatura estimam uma mortalidade de até 55% em pacientes com trauma crânio encefálico moderado a grave, a depender da pressão intracraniana [Teggiari 2007].

*Grupo placebo*

Dentro do período de 12 horas após o TCE, os participantes do grupo placebo receberão 1 mL do mesmo excipiente utilizado na produção do medicamento ativo, onde será diluído em 10 mL de soro fisiológico 0,9% e aplicado por via intravenosa lentamente. O tratamento será repetido a cada 6 horas durante 10 dias consecutivos. Em caso de alta hospitalar no período inferior a 10 dias, impossibilitando a conclusão do protocolo no ambiente hospitalar, o participante poderá ser incluído na análise do estudo (por intenção de tratar) caso ele tenha utilizado 30 doses ou mais do Biperideno. Caso o participante não tenha atingido essas 30 doses mínimas será realizado uma notificação de desvio de protocolo.

*Co-intervenções*

O tratamento convencional de suporte para manejo do TCE recebido pelos participantes seguirá o protocolo do hospital participante, contudo, será recomendado pelo centro coordenador que os hospitais sigam as diretrizes do ministério da saúde para o tratamento de TCE [MS 2015]. Será ainda perguntado no questionário de *feasibility* se o hospital faz uso destas diretrizes e ainda o material publicado pelo MS será encaminhado as equipes de pesquisa do centros participantes. Como a geração da sequência de alocação (descrita em detalhes adiante) será central e estratificada por centro participante, pelos preceitos deste tipo de randomização, espera-se que as diferenças das co-intervenções sejam distribuídas de modo semelhante entre os dois grupos, permitindo que qualquer efeito observado seja devido ao uso ou não do biperideno.

**Métodos para garantir o mascaramento**

O estudo será duplo-cego, o que implica no uso de métodos apropriados para manter todos os envolvidos no estudo (participantes, equipe responsável pela administração da intervenção, avaliadores dos desfechos e estatísticos) sem conhecimento do grupo de alocação de cada participante.

Para tal, o biperideno e o placebo devem apresentar igual aspecto físico (cor, odor, forma) e serão embalados em frascos idênticos, assim como serão os frascos de soro fisiológico utilizados para diluição.

**Método de geração da sequência de alocação**

*Método:* Os participantes serão randomizados por uma central de randomização, que será o centro coordenador (HSL), para receber uma das duas intervenções: biperideno ou placebo. A randomização será realizada por meio de um programa *online* de randomização ([www.random.org](http://www.random.org) ou [www.randomizer.org](http://www.randomizer.org)), com geração de uma tabela de números randômicos. Cada número desta tabela de randomização corresponderá a uma das duas intervenções.

*Tipo:* randomização estratificada considerando a gravidade do TCE (moderado ou grave) e o centro participante.

*Taxa:* a taxa de randomização assumida será de 1:1.

*Unidade*: a unidade de randomização assumida será o participante.

**Método para manutenção do sigilo de alocação**

A tabela de randomização será mantida em sigilo completo pela central de randomização. O registro desta medicação será somente acessado pela equipe de métodos do centro coordenador da pesquisa (HSL), os quais não terão acesso aos participantes do estudo.

A central de randomização disponibilizará cada número randômico via plataforma digital no qual o centro participante terá acesso disponível.

Assim que um participante preencher os critérios de elegibilidade e for incluído no estudo, um membro da equipe do centro coordenador delegado acessará a plataforma digital e terá acesso ao numero do frasco do medicamento a ser administrado. A sequência numérica deverá ser seguida rigorosamente e este processo será verificado nas visitas de monitoria. A plataforma digital deverá ser acessada apenas imediatamente antes de o paciente receber a primeira dose da intervenção.

O membro da equipe do centro coordenador delegado se comunicará com a farmácia local para dizer-lhe o número do frasco-ampola que deverá ser dispensado. A farmácia local entregará à enfermeira as ampolas identificadas com os números randômicos da plataforma digital. Ambos não saberão qual o conteúdo do frasco.

A enfermeira administrará a intervenção ao paciente, conforme detalhado no protocolo.

**Desfechos**

Primários

*Eficácia:*

- Epilepsia pós-traumática: avaliada pela proporção de participantes que desenvolveram EPT no período compreendido entre sete dias e 24 meses após o TCE. A presença de EPT deverá ser confirmada clinicamente. EPT será definida como a ocorrência de pelo menos duas crises não provocadas, ocorrendo em um período superior a sete dias pós TCE [Verellen 2010].

*Segurança:*

- Eventos adversos graves: proporção de participantes que apresentaram pelo menos um evento adverso grave durante 24 meses após da inclusão no estudo. Os eventos considerados graves são definidos como aqueles que resultam em morte, ameaçam a vida, requerem internação hospitalar ou prolongamento da hospitalização existente, resultam em incapacidade (incapacidade persistente ou significativa), ou anomalias congênitas (defeitos de nascimento), suspeita de transmissão de agente infeccioso por meio de medicamento [Anvisa 2016]. As informações referentes a ocorrência deste desfecho serão compiladas no formulário padronizado individual de cada participante, que será preenchido pela equipe de pesquisa ao longo do projeto a partir de interrogatório ativo direto aos participantes e também a partir de relatos espontâneos dos mesmos.

Secundários

*Eficácia:*

- Epilepsia pós-traumática: avaliada pela proporção de participantes que desenvolveram EPT nos seguintes momentos de avaliação: 1, 3, 6, 9, 12, e 18 meses após a inclusão no estudo.
- Qualidade de vida: avaliada pela ferramenta EuroQoL 5D (EQ-5D) [EuroQoL Group 1990] nos seguintes momentos de avaliação: 3, 6, 12, e 24 meses após a inclusão no estudo. O EQ-5D é um questionário validado, com versão validada em português, abrangendo cinco domínios de qualidade de vida relacionada à saúde (mobilidade, cuidados usuais, atividades habituais, dor/mal-estar, ansiedade/depressão).
- Função neuropsicológica: serão aplicados testes que indiquem a medida geral da inteligência, atenção, memória auditivo-verbal e visual, memória operacional, destreza visual-motora e flexibilidade cognitiva. Os seguintes instrumentos serão aplicados após 6 e 24 meses da inclusão no estudo:
- Itens da Bateria da Escala Wechsler de Inteligência - IV [Wechlsler 2008]:
  - Dígitos: O sujeito deve repetir, oralmente, uma série de sequências numéricas apresentadas em ordem direta e inversa. Avalia a memória imediata e a memória operacional.
  - Vocabulário: Deve ser explicado verbalmente o significado de cada uma das 33 palavras apresentadas oralmente ao sujeito. Avalia o desenvolvimento da linguagem, conhecimento de palavras e memória de longo prazo.
  - Cubos: Reproduzir, com cubos bidimensionais e bicolores, os modelos apresentados como padrões. Avalia a percepção visual de estímulo abstrato, organização espacial, planejamento, coordenação visomotora, análise e síntese.
  - Dígitos e Símbolos: o sujeito deve preencher sob pressão de tempo uma folha contendo símbolos, associando-os a dígitos de acordo com um modelo apresentado. A aplicação tem duração aproximada de 60 minutos, o que para o paciente que acaba de sair da internação hospitalar pode ser demorada, mas ao longo da recuperação vão se tornando viáveis. É necessário um intervalo mínimo de 6 meses entre as aplicações para que não ocorra efeito de aprendizagem.
- Figura complexa de Rey-Osterrieth: o sujeito é solicitado a copiar uma figura complexa e, após 5 minutos e sem o modelo, redesenhar de memória. Permite avaliar as habilidades de organização visuo-espacial, planejamento e desenvolvimento de estratégias, bem como memória [Oliveira 1999].
- Teste do Aprendizado Auditivo-Verbal de Rey. O sujeito é solicitado a repetir uma lista de 15 palavras lida em voz alta pelo examinador. O procedimento é repetido 5 vezes. Após isto é introduzida uma segunda lista (estímulo distrator). Em seguida, o sujeito é solicitado a dizer a lista original de memória. Após 15 minutos, nova recordação é solicitada [Rey 1941].
- Teste dos Cinco Dígitos: investiga a flexibilidade cognitiva e o controle inibitório. O sujeito é solicitado, de modo alternado, a: (a) contar o número de estímulos apresentados e (b) dizer os dígitos impressos [Sedo 2005].
- Padrão eletroencefalográfico (EEG): avaliado pelos traçados realizados logo após o TCE em 1, 3, 6, 9, 12, 18 e 24 meses.
- Pesquisa de Apolipoproteína E (ApoE), considerado fator prognóstico importante para o desenvolvimento de sequelas neurológicas pós-traumáticas. O material biológico para a análise será coletado por sangue periférico, no momento da internação hospitalar.

*Segurança:*

- Mortalidade geral: avaliada pela proporção de participantes que morreram após término do período de seguimento de dois anos de todos os participantes.
- Eventos adversos não-graves: avaliados pela proporção de participantes que apresentaram pelo menos um evento adverso não-grave nos seguintes momentos de avaliação: 1, 3, 6, 9, 12, 18 e 24 meses após a inclusão no estudo. As informações referentes a ocorrência deste desfecho serão compiladas no formulário padronizado individual de cada participante, que será preenchido pela equipe de pesquisa ao longo do projeto a partir de interrogatório ativo direto aos participantes e também a partir de relatos espontâneos dos mesmos.

Um seguro será contratado para a cobertura de todos os participantes recrutados a participar desta pesquisa e está previsto no orçamento do projeto.

**Período de seguimento**

Os participantes serão acompanhados por um período de 24 meses a partir da inclusão do estudo por meio de consultas médicas após 1, 3, 6, 9, 12, 18 e 24 meses da inclusão no estudo. Deste modo, os dados finais dos estudos estarão disponíveis após o prazo deste triênio.

Na alta hospitalar, cada paciente receberá um diário de crises epilépticas padronizado e será orientado sobre como preenchê-lo, considerando a ocorrência das crises e deverá apresentá-lo em cada consulta de acompanhamento.

Os pacientes poderão realizar exames (TC, RM, EEG) em momentos específicos durante o período de hospitalização e por 24 meses após a inclusão no estudo. Deste modo, o período de seguimento avançará para o triênio seguinte ao início do projeto. Os momentos de avaliação serão detalhados nas seções ‘Desfechos’ e ‘Cronograma e Atividades’ (item VI).

**Planejamento estatístico e análises**

Serão realizadas as seguintes análises:

- Análise intra-grupo: para avaliar o comportamento da variável ao longo do tempo dentro de um único braço de intervenção.
- Análise inter-grupos: para avaliar os desfechos de interesse, considerando a ocorrência dos eventos ou médias dos escores entre os dois braços de intervenção em todos os timepoints planejados). Estas serão as análises de maior interesse, pois por meio delas é que é possível comparar os efeitos de duas ou mais intervenções em um ECR.

Para avaliar as características da distribuição dos dados (gaussiana ou não), serão aplicados os testes de Kolmogorov-Smirnov e Shapiro-Wilk. Para os testes serão utilizadas suas respectivas tabelas de valores críticos de acordo com a amostra e o nível de significância (valor assumido: 0,05) . Para o teste de Kolmogorv-Smirnov, se o valor calculado pelo teste for maior que o valor crítico, a hipótese de normalidade dos dados será rejeitada. Para o teste de Shapiro-Wilk, a hipótese de normalidade dos dados será rejeitada se o valor calculado for menor que o valor crítico.

Para comparar os resultados médios obtidos entre os grupos (denominado efeito de grupo) e também, ao mesmo tempo, entre dois momentos (1, 3, 6, 9, 12, 18 e 24 meses), será aplicada a análise de variância (ANOVA) com medidas repetidas, considerando que as medidas ao longo do tempo são relacionadas ao mesmo paciente. Caso a distribuição dos dados não seja gaussiana, será utilizado o teste de Friedman, com o teste de Wilcoxon para análise post-hoc.

Para as variáveis dicotômicas, será utilizado o teste qui-quadrado ou teste exato de Fisher (menos de cinco eventos em uma célula da tabela de contingência) para comparar a frequência de eventos entre os dois grupos de intervenção.

Para todos os testes, será considerado um nível de significância de 5% e os dados serão analisados por intenção de tratar (ITT) e *per protocol*. Caso haja diferença nos resultados encontrados com as duas análises, os resultados da análise ITT serão considerados como principais. Em caso de perdas de dados ou de participantes, as análises ITT serão realizadas utilizando métodos apropriados para imputação dos dados faltantes (*last observation carried forward - LOCF*, média dos participantes remanescentes ou abordagens mistas).

Para as variáveis dicotômicas, será utilizado o teste qui-quadrado ou teste exato de Fisher para comparar a frequência de eventos entre os dois grupos de intervenção.

**Previsão de duração do estudo**

Estima-se a participação de nove centros e é esperado que sejam incluídos em torno de 3 a 5 participantes por centro/mês, o que implica em 14 a 18 meses para a inclusão dos 312 participantes. O tempo total estimado para a duração do estudo (fase de intervenção de 10 dias seguida de fase de acompanhamento de 24 meses) é de 38 a 42 meses, ou seja, avançando para o triênio seguinte ao triênio de início do projeto.

**Análise interina**

Uma análise interina será realizada após os primeiros 156 participantes haverem concluído 12 meses de estudo. Isso provavelmente ocorrerá 26 a 31 meses após a inclusão do primeiro participante. Assim, a análise interina será conduzida após o período que compreende o triênio no qual o projeto foi iniciado.

**Critérios para interrupção do estudo**

Na análise interina, o estudo pode ser encerrado por razão de futilidade caso os resultados encontrados indiquem que mesmo com a continuidade do estudo (considerando o cenário mais favorável ao biperideno) os resultados finais mostrariam ausência de benefício ou, na melhor das hipóteses, um benefício clinicamente não relevante.

**Critérios para exclusão de participante após início do estudo**

O participante será excluído do estudo em casos de gestação, manifestação de desejo de descontinuar sua participação, ocorrência de eventos adversos graves e que coloquem em risco a vida do participante (a critério do investigador em discussão com o comitê de monitoramento); caso tenha havido erro de screening ao incluir pacientes com histório de epilepsia/glaucoma ou qualquer outra condição de saúde prévia ao TCE e prevista nos critérios de exclusão do estudo (quando este achado não puder ocorrer no momento do recrutamento); qualquer outra condição que, na opinião do investigador, seja vantajoso para o paciente o não cumprimento dos procedimentos especificados neste protocolo.

Os participantes que não completaram as doses mínimas necessárias (30 doses), por motivo de alta hospitalar, óbito, ou qualquer outra intercorrência no qual impossibilite a finalização das administrações do medicamento serão considerados desvios de protocolo.

**Monitoramento**

Antes do início do estudo, será constituído um comitê (*ad hoc*), composto por dois pesquisadores externos e um pesquisador interno, com o objetivo central de:

- Supervisionar o progresso do ensaio clínico e garantir que ele seja conduzido, registrado e relatado de acordo com o protocolo, Procedimentos Operacionais Padrão (POPs), e Boas Práticas Clínicas [Imperial College 2007] e os requisitos regulatórios aplicáveis.
- Desenvolver e atualizar um documento que descreva estratégias, métodos, responsabilidades e requisitos para monitorar o ensaio clínico.

As atividades do comitê de monitoramento incluem:

- Monitorar a conformidade entre a execução do estudo e o protocolo, por meio de atividades periódicas e aleatórias para:
  - observar a adequação aos critérios de elegibilidade
  - observar a adequação ao cronograma
  - observar a aderência do paciente/equipe ao uso da intervenção
  - observar o cumprimento dos métodos de randomização e sigilo de alocação
  - assegurar que os dados do estudo sejam relatados de modo preciso, completo e verificável a partir dos documentos de origem.
- Monitorar aspectos de segurança, por meio de atividades periódicas e aleatórias para:
  - assegurar os direitos e o bem-estar dos participantes
  - ocorrência de eventos adversos graves
  - avaliar critérios para interrupção do estudo
  - assegurar aderência às recomendações e diretrizes de ética em pesquisa e boas práticas clínicas [ICH-6].

Sobre os membros do comitê:

- devem ser treinados adequadamente e devem ter o conhecimento científico e / ou clínico necessário para monitorar o julgamento adequadamente.
- suas qualificações devem ser documentadas.
- devem estar familiarizados com a intervenção investigada, o protocolo, o consentimento informado e com quaisquer outras informações por escrito a serem fornecidas aos participantes, os procedimentos padronizados e as diretrizes de boa práticas clínicas.

Cabe ainda ao comitê a definição do método de monitoramento: *in loco*, centralizado ou uma combinação de ambos, se justificado. A lógica da estratégia de monitoramento escolhida deve ser documentada.

| **Objetivo 3.** **Avaliar a custo-efetividade do biperideno** |
| --- |

**Contexto**

A análise de custo-efetividade é um tipo de avaliação econômica que compara duas ou mais alternativas de tratamento, em relação aos custos e consequências associados. A análise de custo-efetividade é realizada somente em situações nas quais as alternativas já tenham sido avaliadas, tendo havido constatação dos aspectos de efetividade e segurança.

Deste modo, considerando a premissa acima, o estudo de custo-efetividade será conduzido apenas se os resultados do ensaio clínico tenham comprovado a efetividade e segurança do biperideno, em termos de significância estatística e relevância clínica. Assim, se realizado, o estudo de custo-efetividade será desenhado e conduzido após o período que compreende o triênio no qual o projeto foi iniciado.

**Dados de efetividade**

Para a avaliação econômica serão utilizados os dados de efetividade obtidos a partir do ensaio clínico randomizado (abordagem *bottom up*) [Drummond 2006; Glick 2015].

**Dados de custos**

A aferição dos custos ocorrerá em três etapas, especificamente

(i) identificação do tipo de recurso utilizado por cada participante (ex: diárias de internação em UTI, exames complementares, medicamentos).

(ii) quantificação dos recursos utilizados (consumo de recursos)

(iii) atribuição de valores financeiros de cada recurso utilizado.

Considerando que a execução deste estudo depende da confirmação de efetividade e segurança do biperideno determinada pelo ensaio clínico, os dados de custos referentes aos itens (ii) e (iii) não serão coletados durante o ensaio clínico, e poderão ser coletados posteriormente dos próprios centros participantes ou poderão originar de outras fontes, como os valores médios pagos pelo SUS. Todas as fontes utilizadas serão explicitamente apresentadas.

**Modelagem**

A modelagem será realizada com modelo de árvore decisória, utilizando-se software TreeAge, R ou Excel.

**Desfecho principal planejado**

A análise de custo-efetividade se baseará na razão incremental de custo-efetividade entre as duas intervenções comparadas no ensaio clínico.

**Horizonte temporal**

O horizonte temporal a ser avaliado será de dois anos, considerando o tempo de seguimento dos participantes do ensaio clínico.

**Taxa de desconto**

Será utilizada a taxa de desconto de 5%, tanto para os custos incorridos como para os efeitos clínicos observados no segundo ano de seguimento.

**Perspectiva**

A perspectiva da análise de custo-efetividade será a do SUS. Esta perspectiva norteará os recursos a serem considerados na análise. Despesas incorridas por outras partes, como, por exemplo, despesas custeadas pelo participante de pesquisa, serão, portanto, excluídas da análise.

A inclusão dos custos indiretos na análise, tais como custos administrativos, dependerá da disponibilidade destes dados.

**Análises de sensibilidade**

Serão realizadas análises de sensibilidade determinísticas e probabilísticas para redução da incerteza associada aos parâmetros do modelo.

As análises de sensibilidade determinísticas serão realizadas com variação das taxas de desconto e dos valores dos recursos obtidos de diferentes fontes:

- Em relação à taxa de desconto: serão aplicados os valores de 3% e 7%, para custos e efeitos clínicos, no segundo ano de tratamento;
- Em relação aos custos assumidos: serão aplicadas variações de 10% para mais e para menos a partir dos valores iniciais determinados.

As análises de sensibilidade probabilísticas serão realizadas por simulações de Monte Carlo, com 1000 iterações, utilizando-se a distribuição gama para os custos e a distribuição normal para os efeitos clínicos.

1. **CRONOGRAMA DE ENTREGAS, ATIVIDADES E MARCOS**

Diante do ora exposto, abaixo, o cronograma completo atualizado do projeto para execução no triênio vigente (2021-2023).

Todos os esforços serão concentrados para finalização do estudo em 2023, entretanto se não obtivermos sucesso na etapa de inclusão de pacientes, com número de pacientes incluidos abaixo do esperado para o período, poderá ser solicitado período adicional no triênio subsequente, para finalização do estudo.

**CRONOGRAMA DE ENTREGAS**

| **Cronograma** | **2021** | | | | **2022** | | | | **2023** | | | |
| --- | --- | --- | --- | --- | --- | --- | --- | --- | --- | --- | --- | --- |
| **Entregas/Atividades** | **1º Sem.** | | **2º Sem.** | | **1º Sem.** | | **2º Sem.** | | **1º Sem.** | | **2º Sem.** | |
|  | **P** | **E** | **P** | **E** | **P** | **E** | **P** | **E** | **P** | **E** | **P** | **E** |
| **Entrega 1 - Planejamento** |  |  |  |  |  |  |  |  |  |  |  |  |
| Atividade 1.1 - Seleção de centros, feasibility, convite e aceite de participação | X |  | X |  |  |  |  |  |  |  |  |  |
| **Entrega 2 - Desenho dos Fluxos do Projeto e etapa regulatória** |  |  |  |  |  |  |  |  |  |  |  |  |
| Atividade 2.1 - Submissão aos CEPS dos centros participantes | X |  | X |  | X |  |  |  |  |  |  |  |
| Atividade 2.2 - Desenvolvimento da CRF / Banco de dados | X |  | X |  |  |  |  |  |  |  |  |  |
| Atividade 2.3 - Termo de acordo firmado com os centros participantes | X |  | X |  | X |  |  |  |  |  |  |  |
| **Entrega 3** - **Recursos para o projeto** |  |  |  |  |  |  |  |  |  |  |  |  |
| Atividade 3.1 - Contratação do seguro | X |  | X |  |  |  |  |  |  |  |  |  |
| Atividade 3.2 - Aquisição medicamentos | X |  | X |  | X |  |  |  |  |  |  |  |
| Atividade 3.3 - Aquisição dos testes neuropsicológicos |  |  | X |  |  |  |  |  | X |  |  |  |
| Atividade 3.4 – Coleta e Análise de exames laboratoriais |  |  |  |  | X |  | X |  |  |  |  |  |
| Atividade 3.5 – Armazenamento e Transporte de medicamentos |  |  |  |  | X |  | X |  |  |  |  |  |
| Atividade 3.6 - Contratação de serviços de neuropsicológicos |  |  | X |  |  |  |  |  |  |  | X |  |
| **Entrega 4** **- Intervenção e acompanhamento** |  |  |  |  |  |  |  |  |  |  |  |  |
| Atividade 4.1 - Inclusão de participantes |  |  |  |  | X |  | X |  |  |  |  |  |
| Atividade 4.2 -Seguimento clínico |  |  |  |  | X |  | X |  | X |  | X |  |
| **Entrega 5** - **Monitoramento** |  |  |  |  |  |  |  |  |  |  |  |  |
| Atividade 5.1 - Desenvolvimento plano de monitoria e materiais | X |  | X |  |  |  |  |  |  |  |  |  |
| Atividade 5.2 - Visitas de monitoramento (dados e documentos) |  |  |  |  | X |  | X |  | X |  | X |  |
| Atividade 5.3 - Meeting Investigadores |  |  | X |  |  |  | X |  |  |  |  |  |
| Atividade 5.4 - Monitoramento dos indicadores do projeto |  |  |  |  | X |  | X |  | X |  | X |  |
| **Entrega 6 - Publicação dos resultados** |  |  |  |  |  |  |  |  |  |  |  |  |
| Atividade 6.1 - Envio do banco de dados ao MS e aprovação para publicação |  |  |  |  |  |  |  |  |  |  | X |  |
| Atividade 6.2 -Meeting final investigadores |  |  |  |  |  |  |  |  |  |  | X |  |
| Atividade 6.3 - Elaboração de artigos e publicações |  |  |  |  | X |  |  |  |  |  | X |  |
| Atividade 6.4 - Apresentação em congresso |  |  |  |  |  |  |  |  |  |  | X |  |

**/**

**CRONOGRAMA DE AVALIAÇÕES DO ESTUDO-BIPERIDENO**

| **Tratamento** | 5 mg (1 mL de volume total) de lactato de biperideno. |
| --- | --- |
| **Placebo** | 1 mL de lactato diluído em água para injeção estéril. |
| **Via** | Via intravenosa 6/6 horas |
| **Período de Tratamento** | **(rand.) D1, D2, D3, D4, D5, D6, D7, D8, D9, D10. Entede-se como D1 o dia da randomização no qual o paciente DEVE receber o medicamento/placebo.** |
| **Período de Seguimento 1** | **Seguimentos essenciais a partir do D1: 3, 12 e 24 meses**  **As visitas de seguimento devem ser agendadas na alta do paciente. Para estes pontos do seguimento pode-se tolerar uma variação de ± 5 dias (por conta de feriados ou indisponibilidade), contudo, os centros serão orientados a evitar que se fuja da data correta.** |
| **Período de seguimento 2** | **Seguimento de paciente para avaliação da evolução clínica:1, 6, 9 e 18 meses a partir do D1.**  **As visitas de seguimento devem ser agendadas na alta do paciente. Para estes pontos do seguimento pode-se tolerar uma variação de ± 15 dias (por conta de feriados ou indisponibilidade), contudo, os centros serão orientados a evitar que se fuja da data correta.** |

| **Entregas** | | | |
| --- | --- | --- | --- |
|  | **Detalhamento** | | |
| **Entrega 1** | **Planejamento e Coodenação do Projeto** |  |  |
| **Entrega 2** | **Desenho dos fluxos do projeto e etapa regulatória** |  |  |

| **Entrega 3** | **Recursos necessários ao projeto** |  |  |
| --- | --- | --- | --- |
| **Entrega 4** | **Inclusão de Pacientes** |  |  |
| **Entrega 5** | **Monitoria do estudo** |  |  |
| **Entrega 6** | **Estudo de custo-efetividade** |  |  |
| **Entrega 7** | **Análise dos dados** |  |  |
| **Entrega 8** | **Publicação dos resultados** |  |  |

| **Identificação dos Marcos** |
| --- |

| **Marco** |  |
| --- | --- |
| M.1 | Pactuação com todos os hospitais – Relacionado à entrega 2. Minutas de contrato validadas e assinadas por todos os centros participantes e pelo HSL |
| M.2 | Aprovação comitê de ética – Relacionado à entrega 2. Recebimento do parecer de aprovação do comitê de ética local de todos os centros participantes. A finalização desta entrega é essencial para o cumprimento da legislação de Pesquisa Clínica e inicio do recrutamento. |
| M.3 | Pacientes recrutados – Relacionado à Entrega 5. Este marco refere-se aos pacientes recrutados no estudo. Foi feito um cálculo estatístico que demonstra o tamanho amostral necessário para que os resultados deste estudo tenham poder estatístico e possam ser extrapolados para a população. Este marco será atingido quando este tamanho amostral for atingido. O tamanho amostral e seu cálculo está descrito detalhadamente na metodologia. |
| M.4 | Dados finais analisados e publicados – Relacionado à entrega 6. A publicação científica do estudo e seu último marco, fechando a última atividade e entrega pactuada no presente plano. A publicação científica é a maneira mais efetiva de comunicar os resultados de um projeto de pesquisa. |

1. **EVENTOS ANUAIS DE AVALIAÇÃO DE RESULTADOS**

Reunião de trabalho com o Ministério da Saúde, em dezembro de 2020, para monitoramento do projeto e avaliação do realizado dentro do triênio, considerando que os resultados da pesquisa serão possíveis de serem apresentados após dois anos de seguimento dos pacientes.

1. **PLANO DE GESTÃO DE RISCOS**

O **Quadro 1** apresenta o mapeamento e a descrição dos riscos do projeto, as propostas de intervenção para minimizá-los e a resposta esperada para estas intervenções. Considerar ainda os seguintes pontos de mitigação de possíveis riscos:

- - - 1. Se o paciente estiver internado ainda estiver internado 1 mês após D1 – fazer a visita no hospital.
      2. Se o paciente for de alta até ± 10 antes de completar 1 mês do D1 – realizar a avaliação de “1 mês” antes que o paciente vá de alta (para evitar de perder este primeiro mês de seguimento).
      3. Contato do paciente: para evitar perda no seguimento o centro deverá coletar ao menos 3 telefones do paciente ou alguém relacionado a ele e preencher na ficha de coleta de dados eletrônico
      4. Para cada paciente deve-se colocar em seu leito (durante a internação) uma placa indicando a participação do mesmo no estudo. Isto também deverá constar na evolução médica assim que o paciente for randomizado e intervenção prescrita – no D1. Este documento de evolução será monitorado
      5. Troca de equipe: O centro tem 48h para informar o patrocinador (HSL) sobre toda e qualquer troca de equipe, para que o recrutamento não seja afetado e para que os novos profissionais estejam devidamente treinados e delegados.

1. **DIVULGAÇÃO DOS RESULTADOS DO PROJETO**

É importante ressaltar que as divulgações aqui previstas só serão viáveis quando finalizada a análise dos dados no triênio subsequente. Para a divulgação dos resultados do projeto, estão previstas as seguintes atividades:

- Elaboração de dois manuscritos:
  - Um manuscrito com o relato dos resultados finais do ensaio clínico randomizado, elaborado seguindo o CONSORT [Schulz 2010], para ser submetido em revista científica indexada preferencialmente no ISI-Clarivate Analytics e
  - Um manuscrito com o relato do estudo de custo-efetividade, elaborado seguindo o CHEERS [Husereau 2013], para ser submetido em revista científica (indexada preferencialmente no ISI-Clarivate Analytics).
- Dois pôsteres ou apresentações orais para serem submetidos a congressos internacionais da área clínica de interesse e/ou área de metodologia/farmacoeconomia.

1. **INFORMAÇÕES ADICIONAIS E ANEXOS**

**Sobre a produção científica e publicação dos resultados**

O delineamento do escopo das produções científicas que constituem entrega do projeto pactuada com o Ministério da Saúde, assim como a definição dos colaboradores envolvidos, deve ser definido pela liderança do projeto. A figura de liderança é geralmente exercida pelo gerente de projetos, pelo coordenador do projeto de pesquisa ou ainda pelo product owner (P.O).

As produções científicas que compõem entregas pactuadas no plano de trabalho devem ser priorizadas, em relação aos outros tipos de produção no âmbito do projeto PROADI-SUS. Esta compreensão é importante para que as produções suplementares eventualmente desenvolvidas não incorram na situação de duplicidade científica, isto é, na dupla publicação dos mesmos resultados de um estudo. Portanto, é recomendado que o escopo das produções científicas previstas seja definido precocemente, ao longo da condução do projeto, para que se identifique as janelas de oportunidade para a produção científica suplementar.

A definição dos papeis dos autores deve ser realizada na fase de planejamento do estudo, sempre que possível2. A definição precoce dos papéis dos autores aumenta a transparência em relação às atribuições da equipe de pesquisadores, alinhando as expectativas individuais à ordem de autoria do produto final. Esta definição é especialmente importante para os autores com papel destaque, como primeiro autor, último autor e o autor de correspondência.

A seleção de autores deve ser orientada pelo preenchimento dos quatro critérios de reconhecimento de autoria propostos pelo ICMJE.

**Critérios de autoria ICMJE (2):**

1 – Contribuições substanciais para a concepção ou design da obra; ou a aquisição, análise ou interpretação de dados para o trabalho; E

2 – Elaboração do trabalho ou revisão crítica do mesmo quanto a seu conteúdo intelectual importante; E

3 – Aprovação final da versão a ser publicada; E

4 – Responsabilidade por todos os aspectos do estudo garantindo que as questões relacionadas à precisão ou integridade de qualquer parte do estudo sejam devidamente investigadas e resolvidas.

Deve-se garantir que todos os autores envolvidos na produção científica tenham justa oportunidade de dar as suas contribuições, para que possam legitimar sua autoria. Da mesma forma, deve-se assegurar que todos que tenham contribuído para os quatro critérios do ICMJE estejam incluídos entre os autores.

No que tange à reunião com os investigadores participantes e apresentação de resultados em congresso internacional. É proposto neste plano de trabalho uma reunião virtual com todos os investigadores, SCITE e ANVISA para apresentar o andamento do estudo, discutir possíveis entraves e capacitação científica dos investigadores dos centros de pesquisa. É proposto ainda uma reunião de investigadores a realizar-se ao final do triênio em dezembro de 2023 para apresentação de resultados preliminares e discussão dos estudos relacionados à plataforma. É previsto ainda a ida dos investigadores principais ao Hospital Sírio-Libanês para apresentação dos resultados preliminares dos estudos.

1. **REFERÊNCIAS**

Antoniuk, SA & Schwind, MR. Crisis no epilépticas en la infancia y adolescencia. MEDICINA (Buenos Aires),2013; 73.

Annegers JF, Hauser WA, Coan SP, Rocca WA. ‘A Population-Based Study of Seizures after Traumatic Brain Injuries. N England J of Med. 1998;338(1):20-4.

Aronstam, RS & Patil, P. Muscarinic Receptors: Autonomic Neurons, Encyclopedia of Neuroscience, Academic Press,2009; 1141-1149, ISBN 9780080450469, https://doi.org/10.1016/B978-008045046-9.00692-6.

Asikainen I Kaste, M., Sarna, S. Early and late posttraumatic seizures in traumatic brain injury rehabilitation patients: brain injury factors causing late seizures and influence of seizures on long-term outcome. Epilepsia. 1999;40(5):584-9.

Anvisa. MANUAL PARA NOTIFICAÇÃO DE EVENTOS ADVERSOS E MONITORAMENTO DE SEGURANÇA EM ENSAIOS CLÍNICOS - 1a. edição. 2016. Disponível em: <http://portal.anvisa.gov.br/documents/33836/2492465/Manual+para+Notifica%C3%A7%C3%A3o+de+Eventos+Adversos+e+Monitoramento+de+Seguran%C3%A7a+em+Ensaios+Cl%C3%ADnicos+-+1%C2%AA+Edi%C3%A7%C3%A3o/04a68574-8aac-43c9-b0b2-7b7cd80831c4>. Acessado em 5 de novembro de 2019.

Brady, RD., Casillas-Espinosa, PM., Agoston, DV., Bertram, EH., Kamnaksh, A., Semple, BD., & Shultz, SR. Modelling traumatic brain injury and posttraumatic epilepsy in rodents. Neurobiology of disease, 2019; *123*, 8-19.

Benassi SK, Alves JGSM, Guidoreni CG, Massant CG, Queiroz CM, Garrido-Sanabria E, Loduca RDS, Susemihl MA, Paiva WS, de Andrade AF, Teixeira MJ, Andrade JQ, Garzon E, Foresti ML, Mello LE. Two decades of research towards a potential first anti-epileptic drug. Seizure. 2021 Mar 3:S1059-1311(21)00065-0.

Bittencourt S, Ferrazoli E, Valente MF, et al. Modification of the natural progression of epileptogenesis by means of biperiden in the pilocarpine model of epilepsy. Epilepsy Research. 2017; 138:88-97.

Comissão Nacional de Ética em Pesquisa (CONEP). Resolução no. 466, de 12 de dezembro de 2012. Disponível em: <https://conselho.saude.gov.br/resolucoes/2012/Reso466.pdf>. Acessado em 25 de outubro de 2019.

D’Ambrosio R, Perucca E. Epilepsy after head injury. Current Opinion in Neurology. 2004;17(6):731-5.

da Silva AM, Vaz AR, Ribeiro I, Melo AR, Nune B, Correia M. Controversies in posttraumatic epilepsy'. Acta Neurochir Suppl (Wien).1990; 50:48-51.

de Almeida CE, de Sousa Filho JL, Dourado JC, Gontijo PA, Dellaretti MA, Costa BS. Traumatic Brain Injury Epidemiology in Brazil. World Neurosurg. 2016; 87:540-7.

DeVito NJ, Goldacre B. Catalogue of bias: publication bias. BMJ Evidence-Based Medicine. 2019; 24:53-5.

[Drummond](https://www.amazon.com.br/s/ref=dp_byline_sr_book_1?ie=UTF8&field-author=Michael+F.+Drummond&search-alias=books) MF, [Sculpher](https://www.amazon.com.br/s/ref=dp_byline_sr_book_2?ie=UTF8&field-author=Mark+J.+Sculpher&search-alias=books) MJ, [Torrance](https://www.amazon.com.br/s/ref=dp_byline_sr_book_3?ie=UTF8&field-author=George+W.+Torrance&search-alias=books) GW. Methods for the Economic Evaluation of Health Care Programmes. Oxford University Press, USA; 3a. Edição: 2006.

Englander J, Bushnik T, Duong T. Analyzing risk factors for late posttraumatic seizures: a prospective multicenter investigation. Arch Phys Med Rehabil.2003;84:365-73.

European Medicines Agency (EMA). ICH Harmonised Tripartite Guideline E6: Note for Guidance on Good Clinical Practice (PMP/ICH/135/95) London: European Medicines Agency; 2002. Disponível em: <https://www.ema.europa.eu/en/ich-e6-r2-good-clinical-practice>. Acessado em 24 de outubro de 2019.

EuroQoL Group 1990. EuroQol--a new facility for the measurement of health-related quality of life Health Policy[.](https://www.ncbi.nlm.nih.gov/pubmed/10109801) 1990;16(3):199-208.

Faul M, Coronado V. Epidemiology of traumatic brain injury. Handb Clin Neurol. 2015; 127:313.

French JA, White HS, Klitgaard H, *et al*. Development of new treatment approaches for epilepsy: Unmet needs and opportunities. Epilepsia. 2013;54(Suppl4):3-12.

Gentile JKA, Himuro HS, Rojas SSA, *et al.* Condutas no paciente com trauma cranioencefálico. Rev Bras Clin Med. São Paulo. 2011;9(1):74-82.

Glick HA, Doshi JA, Sonnad SS, Polsky D. Economic Evaluation in Clinical Trials. Oxford University Press, UK; 2a. Edição: 2015.

Gorgati C, Mello LEAM. Caracterização dos efeitos antiepileptogênicos do biperideno em ratos no modelo da pilocarpina PhD thesis, Neurology Graduate Program. Universidade Federal de São Paulo. 2009.

Hauser WA, et al Prevalence of epilepsy in Rochester, Minnesota: 1940-1980'. Epilepsia. 1991; 32:429-45.

Howick J, Chalmers I, Glasziou P, et al for the OCEBM Levels of Evidence Working Group*. “The Oxford Levels of Evidence 2”. 2011.Oxford Centre for Evidence-Based Medicine. Disponível em: <https://www.cebm.net/index.aspx?o=5653>. Acessado em 23 de outubro de 2019.

[Husereau D](https://www.ncbi.nlm.nih.gov/pubmed/?term=Husereau%20D%5BAuthor%5D&cauthor=true&cauthor_uid=23526140), [Drummond M](https://www.ncbi.nlm.nih.gov/pubmed/?term=Drummond%20M%5BAuthor%5D&cauthor=true&cauthor_uid=23526140), [Petrou S](https://www.ncbi.nlm.nih.gov/pubmed/?term=Petrou%20S%5BAuthor%5D&cauthor=true&cauthor_uid=23526140), et al. Consolidated Health Economic Evaluation Reporting Standards (CHEERS) statement. [Eur J Health Econ.](https://www.ncbi.nlm.nih.gov/pubmed/23526140) 201314(3):367-72. doi: 10.1007/s10198-013-0471-6.

Imperial College Clinical Research Governance Office. Good Clinical Practice [Web Page] 2007. Disponível em: <http://www.imperial.ac.uk/clinicalresearchoffice>.Acessado em 24 de outubro de 2019.

Kim, J.A., Boyle, EJ., Wu, AC., Cole, AJ., Staley, KJ., Zafar, S., ... & Westover, MB. Epileptiform activity in traumatic brain injury predicts post‐traumatic epilepsy. Annals of neurology, 2018; *83*(4), 858-862.

Klein P, Tyrlikova I. Prevention of epilepsy: Should we be avoiding clinical trials? Epilepsy Behav.2017;72:188-94.

Maas, AI., Menon, DK., Adelson, PD., Andelic, N., Bell, MJ., Belli, A., … & Citerio, G. Traumatic brain injury: integrated approaches to improve prevention, clinical care, and research. The Lancet Neurology, 2017; *16*(12), 987-1048.

Magalhães ALG, Souza LC, Faleiro RM , Teixeira AL, Miranda AL. Epidemiologia do traumatismo cranioencefálico no Brasil. Revista Brasileira de Neurologia 2017;53(2):15-22.

Masel BE, DeWitt DS. Traumatic brain injury: a disease process, not an event. J Neurotrauma. 2010;27(8):1529-40.

Ministério da Saúde. Secretaria de Atenção à Saúde.Diretrizes de atenção à reabilitação da pessoa com traumatismo cranioencefálico. Brasília: Ministério da Saúde, 2015.

Moran SP, Maksymetz J, Conn PJ. Targeting Muscarinic Acetylcholine Receptors for the Treatment of Psychiatric and Neurological Disorders. Trends in pharmacological sciences. 2019 Nov 8.

NCT01048138.Use of Biperiden for the Prevention of Post-traumatic Epilepsy. Disponível em: <https://clinicaltrials.gov/ct2/show/NCT01048138?term=biperideno&rank=1>. Acessado em 18 de outubro de 2019.

Okamoto OK, Janjoppi L, Bonone FM, *et al*. Whole transcriptome analysis of the hippocampus: toward a molecular portrait of epileptogenesis. BMC genomics. 2010; 11:230.

Oliveira M da S. Figuras Complexas de Rey: teste de cópia e de reprodução de memória de figuras geométricas complexas. Manual André Rey. Revisão técnica Teresinha Rey, Lucia C. F. Franco. Tradução Teresinha Rey, Lucia C. F. Franco. São Paulo: Casa do Psicólogo; 1999

Payan H, Toga M, Bérard-Badier M. The pathology of post-traumatic epilepsies. Epilepsia. 1970;11(1):81-94.

Pereira HAA, Benassi SK, Mello LE. Plastic Changes and Disease-modifying Effects of Scopolamine in the Pilocarpine Model of Epilepsy in Rats. Epilepsia.2005;46(s5):118-124.

Piccenna L, Shears G, O'Brien TJ. Management of post‐traumatic epilepsy: An evidence review over the last 5 years and future directions. Epilepsia. 2017;2(2):123-44.

Pitkänen A. Therapeutic approaches to epileptogenesis - Hope on the horizon’. Epilepsia. 2010; 51(Suppl 3):2-17.

# Pocock SJ. Clinical Trials - a practical approach. John Wiley & Sons, Chichester - New York - Brisbane - Toronto -Singapore 1983, 265 S.

Radu, BM, Osculati, AMM., Suku, E. …Fabene, PF. All muscarinic acetylcholine receptors (M1-M5) are expressed in murine brain microvascular endothelium. Sci Rep 7, 2017; 5083 (7) doi: 10.1038/s41598-017-05384-z

Rawlings DB, Crewe NM. Test-retest practice effects and test score changes of the WAIS-R in recovering traumatically brain-injured survivors. Clinical Neuropsychologist.1992;6:415-30. doi:10.1080/13854049208401868.

Raymont V, Salazar AM, Lipsky R, *et al*. Correlates of posttraumatic epilepsy 35 years following combat brain injury'. Neurology. 2010; 75:224-9.

Rey A. L’examen psychologique dans les cas d’encéphalopathie traumatic. 1941. Arch Psychol 28:286–340.

Rey A. L'examen Clinique En Psychology. Paris: Press Universitaire de France, 1964.

Salazar AM, Grafman J. Epilepsy after penetrating head injury. I. Clinical correlates: a report of the Vietnam Head Injury Study. Neurology.1985;35(10):1406-14.

Salazar AM, Grafman J. Post-traumatic epilepsy: clinical clues to pathogenesis and paths to prevention. Handb Clin Neurol. 2015; 128:525-38.

Schulz KF, Altman DG, Moher D for the CONSORT Group. CONSORT 2010 Statement: updated guidelines for reporting parallel group randomised trials. BMJ. 2010; 340:c332. Disponível em: <https://www.bmj.com/content/340/bmj.c332>. Acessado em 18 de outubro de 2019. doi: <https://doi.org/10.1136/bmj.c332>.

Santos, MF, dos Santos Silva, TDC, de Carvalho, FR, Barbosa, RL, dos Santos, LH, & de Matos Junior, EM. TCE em UTI: Epidemiologia, tratamento e mortalidade no maranhão, brasil. Revista Brasileira de Neurologia e Psiquiatria. 2019: *23*(1).

Sedo MA. Test de los cinco dígitos [Five digit test]. Madrid: TEA Ediciones; 2005

Stroop, J.R. (1935). Studies of interference in serial verbal reaction. Journal of Experimental Psychology, 18, 643-662.

Temkin NR, Dikmen SS, Wilensky AJ, Keihm J, Chabal S, Winn HR. A Randomized, Double-Blind Study of Phenytoin for the Prevention of Post-Traumatic Seizures. N Engl J Med. 1990;323(8):497-502.

Temkin NR. Antiepileptogenesis and Seizure Prevention Trials with Antiepileptic Drugs: Meta-Analysis of Controlled Trials. Epilepsia. 2001;42(4):515-24.

Temkin NR. Risk Factors for Posttraumatic Seizures in Adults Epilepsia. 2003;44(Suppl10):18-20.

Verellen RM, Cavazos JE. Post-traumatic epilepsy: an overview. Therapy. 2010;7(5):527–31. doi:10.2217/THY.10.57

# [von Steinbüchel N](https://www.ncbi.nlm.nih.gov/pubmed/?term=von%20Steinb%C3%BCchel%20N%5BAuthor%5D&cauthor=true&cauthor_uid=20210602), [Wilson L](https://www.ncbi.nlm.nih.gov/pubmed/?term=Wilson%20L%5BAuthor%5D&cauthor=true&cauthor_uid=20210602), [Gibbons H](https://www.ncbi.nlm.nih.gov/pubmed/?term=Gibbons%20H%5BAuthor%5D&cauthor=true&cauthor_uid=20210602), *et al* for [QOLIBRI Task Force](https://www.ncbi.nlm.nih.gov/pubmed/?term=QOLIBRI%20Task%20Force%5BCorporate%20Author%5D).Quality of Life after Brain Injury (QOLIBRI): scale validity and correlates of quality of life.[J Neurotrauma.](https://www.ncbi.nlm.nih.gov/pubmed/20210602) 2010;27(7):1157-65. doi: 10.1089/neu.2009.1077.

Wechsler D. A standardized memory scale for clinical use. Journal of Psychology. 1945; 19:87-95. doi:10.1080/ 00223980.1945.9917223.

Wechsler D. (2008). Wechsler adult intelligence scale–Fourth Edition (WAIS–IV). San Antonio, TX: Pearson

World Medical Association. Declaration of Helsinki. Ethical principles for medical research involving human subjects. [Internet]. 59th WMA General Assembly, Seoul, oct. 2008. Disponível em: <http://www.wma.net/en/30publications/10policies/b3/17c.pdf>. Acessado em 23 de outubro de 2019.

Yablon SA. Posttraumatic seizures. Archives of physical medicine and rehabilitation.1993; 74(9):983-1001.
