# Supplementary material for: Biperiden for prevention of post-traumatic epilepsy: A protocol of a double-blinded placebo-controlled randomized clinical trial (BIPERIDEN trial)
Source: PLoS One. 2022 Sep 9;17(9):e0273584. doi: 10.1371/journal.pone.0273584 (PMC9462738; doi:10.1371/journal.pone.0273584)
Supplement: S3 File — (DOCX) [file pone.0273584.s004.docx]

**CONSTITUTED OPINION FROM THE RESEARCH ETHICS COMMITTEE**

**AMENDMENT DATA**

**Research Title:** Biperiden for the prevention of epilepsy in patients with traumatic brain injury.

**Researcher:** Eliana Garzon

**Thematic Area:**

**Version:** 3

**CAAE:** 39005920.8.1001.5461

**Proposing Institution:** Sociedade Beneficente de Senhoras Hospital Sírio Libanês

**Main Sponsor:** Sociedade Beneficente de Senhoras Hospital Sírio Libanês

**OPINION DATA**

**Opinion Number:** 5.084.793

**Project presentation:**

Design:

Multicenter, double-blind, placebo-controlled randomized clinical trial classified as a phase 3 clinical trial. Study design will follow the recommendations of the Consolidated Standards of Reporting Trials (CONSORT Statement) [Schulz 2010], which despite being developed for guide reporting or publication, has also been widely used to guide the planning of a randomized clinical trial. The study will be planned and developed with due rigor in order to minimize the occurrence of the biases cataloged in the literature to date [de Vito 2019]. The report of the study results will follow the Consolidated Standards of Reporting Trials (CONSORT Statement) [Schulz 2010 ].

Summary:

Traumatic brain injury (TBI) is a public health problem, with considerable socio-economic costs. TBI is one of the most important causes of secondary epilepsy (in this case, called post-traumatic epilepsy, EPT). The pathophysiology of TBI stems from lesions in the brain parenchyma that trigger neurodegenerative and inflammatory responses and lead to molecular, structural and electrophysiological changes that ultimately result in EPT. The therapeutic approach indicated for TBI may involve drugs and/or surgical procedures, and there is still no effective therapeutic intervention to reduce the occurrence of EPT.

Several studies with animal models have shown that drugs that modify neuronal plasticity processes, if administered under certain conditions, have the potential to modify the natural course of EPT. Among these drugs, biperiden (anti-cholinergic for clinical use for Parkinson's) decreased the incidence and intensity of spontaneous epileptic seizures and delayed their onset in an epilepsy model, proving to be an excellent candidate for an anti-epileptogenic agent. The aim here is to test the efficacy and safety of the use of biperiden in adult patients, victims of moderate and severe TBI. Patients will be treated for 10 days after the trauma, in order to avoid the formation of an epileptogenic focus. Prospectively, patients will be followed up at periodic returns over two years to assess the development of epileptic seizures. Other factors that may have benefits or influence treatment will also be evaluated, such as genetic markers and neuropsychological aspects. If positive, this treatment: 1) characterizes a mechanism of action; 2) opens the way for testing new molecules and; 3) is a low-cost option (characterized as a second drug use, available in the SUS). In addition, the present proposal, in addition to typifying a clinical trial, seeks to understand the basic mechanisms of EPT and its prevention.

Introduction:

Traumatic Brain Injury

Defined as altered brain function or evidence of brain pathology caused by an external force, traumatic brain injury (TBI) is responsible for 1% of all adult deaths worldwide [Gentile 2011], which corresponds to 50 million of people around the world every year, being the main cause of mortality and comorbidity in all countries, including Brazil [Maas 2017; Ministry of Health 2015; Pereira 2006]. Data from DATASUS show that about 125,000 people/year receive hospital care due to TBI, which corresponds to a frequency of 65.7 cases / 100,000 inhabitants [Magalhães 2017]. As an additional concern, the hospital mortality rate for these cases reaches 7.7%, i.e. 5.1 deaths/100,000 cases/year [Magalhães 2017]. Traffic accidents represent the main cause of TBI, totaling 50% of cases, followed by falls and urban violence [de Almeida 2016]. Studies show that despite the size of the impact of TBI, strong scientific evidence to support treatment guidelines and recommendations is still scarce, with clinical trials failing to show true treatment efficacy despite promising results [Maas 2017]. with TBI, neurological complications include primary (direct result of trauma) and secondary (initiated after the moment of trauma, derived from primary damage) brain injuries [Faul 2015; Masel 2010].

TBI survivors often experience physical, psychological, emotional and cognitive trauma, which can cause a significant reduction in the individual's quality of life and functionality and bring with it a high socioeconomic impact [Maas 2017]. Post-traumatic epilepsy (EPT) is a neurological complication that occurs in up to 20% of patients, with the risk increasing according to the severity of the TBI, surgical intervention, time interval since the TBI, with this percentage rising to 53% in the most severe cases, with penetrating injuries [Anegers 1998; Asikainen 1999; Englander 2003; Hauser 1991; Kim 2018; Raymont 2010; Salazar 2015; Temkin 1990]. PTSE can be early (when it occurs within one to two weeks after the trauma) or late (after this period). Early seizures may be caused by acute effects of trauma, such as hemorrhage and cerebral edema (and do not necessarily characterize epilepsy), while late seizures seem to depend on synaptic reorganization mechanisms [Payan 1970; Yablon 1993]. Most people who develop epilepsy secondary to head trauma experience epileptic seizures within the first two years after the trauma or injury [da Silva 1990]. As with other types of epilepsy, the causes that link TBI to EPT have not yet been fully elucidated and it is still not possible to avoid the process of epileptogenesis. We were unable to find any strong evidence for a specific treatment to prevent or reverse epileptic seizures after TBI [Brady 2019].

Treatment of TBI and prevention of EPTA

The therapeutic approach indicated for TBI may involve several drugs and/or surgical procedures, for primary and secondary damage care and essentially depends on the extent of the lesion and which areas were affected. Recent therapeutic advances involve drugs targeting secondary damage mechanisms, including calcium channel blockers, corticosteroids, excitatory amino acid inhibitors, N-methyl D-aspartate (NMDA) receptor antagonists, free radical scavengers, magnesium, growth factors [Salazar 1985]. Clinical trials under development using several approaches have great therapeutic potential for TBI such as erythropoietin, statins, bone marrow cells, progesterone. Recent clinical trials have focused on neuroprotective strategies in order to prevent and/or reduce brain damage secondary to TBI [Temkin 1990]. However, none of these interventions appear to influence the occurrence of EPT [French 2013; Klein 2017; Piccena 2017; Pitkänen 2010; Temkin 2001; Temkin 2003].

Anticonvulsant substances are indicated for the control of epileptic seizures that eventually occur in an acute form, but their administration does not prevent the evolution to epilepsy [D'Ambrosio 2004]. In this context, there are no neuroprotective drugs indicated to prevent epileptogenic processes from being established after brain damage, whether traumatic, ischemic, or otherwise [Brady 2019; Temkin 2001]. Seeking to address this gap, studies with animal models have identified that drugs that alter neuronal plasticity processes, if administered under certain conditions, have the potential to modify the natural course of EPT [Bittencourt 2017]. Specifically, regarding the conditions mentioned in the study by Bittencourt, et al., [2017], there is: a) the therapeutic window, that is, the interval and time between the injurious event (TBI) and the administration of the first dose of biperiden (indicated in

our project as having to be equal to or less than 12 h); b) the duration of the modulation of the neuronal plasticity process, ie the duration of treatment with biperiden (indicated in our protocol as 10 days); c) the dose of biperiden, 5 mg every 6 hours. Evidence from preclinical phase studies has indicated that these three conditions above must be met for biperiden to demonstrate effectiveness as an agent capable of demonstrating an action to modify the natural course of the disease [Bittencourt 2017].In experimental studies using epilepsy models, biperiden, an anticholinergic of clinical use for Parkinson's, has been shown to act on neuronal plasticity, reducing the incidence and intensity of spontaneous epileptic seizures and delaying their onset [Bittencourt 2017; Gorgati 2009]. Another study reported the use of biperiden as an antidote for the treatment of dystonia in non-epileptic seizures due to drug reaction [Schwind & Antoniuk 2013]. These findings, added to the scarce literature on the effect of biperiden in the prevention of EPT after TBI, emphasize that biperiden could be a potential therapeutic option in the care of patients with TBI, considering its probable anti-epileptogenic action.

Hypothesis:

Biperiden could be a potential therapeutic option in the care of patients with TBI, considering its likely anti-epileptogenic action.

Proposed Methodology:

Multicenter, double-blind, placebo-controlled, randomized clinical trial classified as a phase 3 clinical trial.

P (population) = subjects with moderate to severe acute TBI, with confirmed intraparenchymal hemorrhage,

I (intervention) = biperiden

C (comparator) = placebo

O (outcomes, outcomes) = effectiveness and safety outcomes (detailed below)

Intervention

Biperiden group Within the 12-hour period after TBI, participants in the biperiden group will receive a dose of 5 mg (1 ml total volume) of biperiden lactate (Cinetol , Cristália, Brazil), diluted in 10 mL of 0.9% saline and applied slowly intravenously. The treatment will be repeated every 6 hours for 10 consecutive days. Co-interventions to conventional supportive treatment for TBI management received by participants will follow the protocol of the participating hospital, however, it will be recommended by the coordinating center that hospitals follow the Ministry of Health guidelines for the treatment of TBI [MS 2015]. It will still be asked in the feasibility questionnaire if the hospital makes use of these guidelines and the material published by the MS will be forwarded to the research teams at the participating centers. As the generation of the allocation sequence (described in detail below) will be central and stratified by participating center, according to the precepts of this type of randomization, it is expected that the differences in the co-interventions will be similarly distributed between the two groups, allowing any effect observed whether due to the use or not of biperiden.

Inclusion Criteria:

Consent to participate provided by the person in charge, who must sign and date the informed consent after being guided about the study by the principal investigator or person in charge, reading and agreeing to the information letter; Age between 18 and 75 years of age; Both sexes; Diagnosis of moderate to severe acute TBI; Glasgow Coma Scale (GCS) greater than 6 to 12 at hospital admission; Presence of acute intraparenchymal hemorrhage and/or contusion confirmed by magnetic resonance imaging (MRI) and/or computed tomography (CT).

Exclusion Criteria:

Vulnerable participants, homeless, without documentation and without a fixed address and/or contact with family members; Undocumented patients of questionable age (18-75 years) at hospital admission; Being using biperiden during the period of TBI occurrence; History of epilepsy (confirmed by medical records and/or use of specific medication and/or referred by the legal guardian); History of seizures or use of antiepileptic medication; History of perinatal injuries, meningitis and/or encephalitis (or other proven or probable risk factor for epilepsy); History of neoplasia, neurodegenerative diseases; hx of cerebrovascular accident (CVA), cognitive dysfunction, benign prostatic hyperplasia, atrioventricular block or any other cardiac arrhythmia, or glaucoma; Gestation; Current participation in another clinical trial; Patients with cardiac arrhythmias or glaucoma, due to the increased risk of developing adverse reactions.

Data Analysis Methodology:

The following analyzes will be performed:

• intra-group analysis: to evaluate the behavior of the variable over time within a single intervention arm.

• inter-group analysis: to evaluate the outcomes of interest, considering the occurrence of events or means of scores between the two intervention arms at all planned timepoints). These will be the analyzes of greatest interest, because through them it is possible to compare the effects of two or more interventions in an RCT.

To evaluate the characteristics of the data distribution (Gaussian or not), the Kolmogorov-Smirnov tests will be applied. and Shapiro-Wilk. For the tests, their respective tables of critical values ​​will be used according to the sample and the level of significance (assumed value: 0.05). For the Kolmogorv-Smirnov test, if the value calculated by the test is greater than the critical value, the hypothesis of normality of the data will be rejected. For the Shapiro-Wilk test, the hypothesis of normality of the data will be rejected if the calculated value is less than the critical value. two moments (1, 3, 6, 9, 12, 18 and 24 months), analysis of variance (ANOVA) with repeated measures will be applied, considering that the measures over time are related to the same patient.

If the data distribution is not Gaussian, the Friedman test will be used, with the Wilcoxon test for post-hoc analysis. For dichotomous variables, the chi-square test or Fisher's exact test (less than five events in a cell of the contingency table) will be used to compare the frequency of events between the two intervention groups. a 5% significance level and data will be analyzed by intention to treat (ITT) and per protocol.

If there is a difference in the results found with the two analyses, the results of the ITT analysis will be considered as the main ones. In case of loss of data or participants, ITT analyzes will be carried out using appropriate methods for imputing missing data (last observation carried forward - LOCF, average of remaining participants or mixed approaches). For dichotomous variables, the chi-square test or Fisher's exact test will be used to compare the frequency of events between the two intervention groups. For all tests, a significance level of 5% will be considered and the data will be analyzed by intention to treat (ITT) and per protocol. If there is a difference in the results found with the two analyses, the results of the ITT analysis will be considered as the main ones. In case of loss of data or participants, ITT analyzes will be carried out using appropriate methods for imputing missing data (last observation carried forward - LOCF, average of remaining participants or mixed approaches).

Primary Outcome:

Primary Efficacy: Post-traumatic epilepsy: assessed by the proportion of participants who developed TPE in the period between seven days and 24 months after TBI. The presence of TPE must be confirmed clinically. EPT will be defined as the occurrence of at least two unprovoked seizures, occurring more than seven days after TBI [Verellen 2010].Safety: Serious adverse events: proportion of participants who experienced at least one serious adverse event after 24 months of inclusion in the study. Events considered serious are defined as those that result in death, threaten life, require hospitalization or prolongation of existing hospitalization, result in disability (persistent or significant disability), or congenital anomalies (birth defects), suspected transmission of the agent. infectious through medication [Anvisa 2016]. The information regarding the occurrence of this outcome will be compiled in the individual standardized form of each participant, which will be filled in by the research team throughout the project, based on direct active interrogation of the participants and also from their spontaneous reports.

Secondary Outcome:

Efficacy: Post-traumatic epilepsy: assessed by the proportion of participants who developed PTSD at the following time points: 1, 3, 6, 9, 12, and 18 months after study enrollment. Quality of life: assessed using the EuroQoL 5D (EQ-5D) tool [EuroQoL Group 1990] at the following time points: 3, 6, 12, and 24 months after enrollment in the study. The EQ-5D is a validated questionnaire, with a validated version in Portuguese, covering five health-related

quality of life domains (mobility, usual care, usual activities, pain/discomfort, anxiety/depression).

Neuropsychological function: tests will be applied that indicate the general measure of intelligence, attention, auditory-verbal and visual memory, working memory, visual-motor dexterity and cognitive flexibility. The following instruments will be applied 6 and 24 months after inclusion in the study: Items from the Wechsler Intelligence Scale Battery - IV [Wechlsler 2008]: Digits: The subject must orally repeat a series of numerical sequences presented in direct and reverse order. Evaluates immediate memory and working memory. Vocabulary: The meaning of each of the 33 words presented orally to the subject must be verbally explained. Assesses language development, word knowledge and long-term memory. Cubes: Reproduce, with two-dimensional and two-color cubes, the models presented as patterns. Assess the visual perception of abstract stimulus, spatial organization, planning, visual-motor coordination, analysis and synthesis. Digits and Symbols: the subject must fill in, under time pressure, a sheet containing symbols, associating them with digits according to a model presented. The application lasts approximately 60 minutes, which for the patient who has just left the hospital can be time consuming, but during the recovery period they become viable. A minimum interval of 6 months is required between applications so that there is no learning effect. Rey-Osterrieth complex figure: the subject is asked to copy a complex figure and, after 5 minutes and without the model, redraw it from memory. It allows assessing visuospatial organization skills, planning and strategy development, as well as memory [Oliveira 1999]. Rey's Auditory-Verbal Learning Test. The subject is asked to repeat a list of 15 words read aloud by the examiner. The procedure is repeated 5 times. After that, a second list (distractor stimulus) is introduced. Then the subject is asked to say the original list from memory. After 15 minutes, a new recall is requested [Rey 1941]. Five-Digit Test: investigates cognitive flexibility and inhibitory control. The subject is asked, alternately, to: (a) count the number of stimuli presented and (b) say the printed digits [Sedo 2005].

Electroencephalographic (EEG) pattern: assessed by tracings taken shortly after TBI at 1, 3, 6, 9, 12, 18 and 24 months.

Apolipoprotein E (ApoE) investigation, considered an important prognostic factor for the development of post-traumatic neurological sequelae. Biological material for analysis will be collected by oral swab or peripheral blood at the time of hospital admission.

Safety: Overall mortality: assessed by the proportion of participants who died after the end of the two-year follow-up period for all participants. Non-serious adverse events: assessed by the proportion of participants who experienced at least one non-serious adverse event at the following time points: 1, 3, 6, 9, 12, 18, and 24 months after study enrollment. The information regarding the occurrence of this outcome will be compiled in the individual standardized form of each participant, which will be filled in by the research team throughout the project, based on direct active interrogation of the participants and also from their spontaneous reports.

**Research Purpose:**

Primary Purpose:

To evaluate the effects (benefits and risks) and cost-effectiveness of using biperiden for the prevention of post-traumatic epilepsy (PTS) in patients with traumatic brain injury (TBI) and confirmed intraparenchymal hemorrhage.

Secondary Objective:

1. Assess the effectiveness of biperiden for preventing EPT.

2. Assess the safety of biperiden for preventing EPT.3. To assess the cost-effectiveness of biperiden for preventing EPT.

**Assessment of Risks and Benefits:**

Risks:

In this research the risks are minimal, however, we emphasize some points that can generate some discomfort. Biperiden is already an approved drug used in the treatment of patients with Parkinson's disease. Every drug has a risk in its use. To reduce this risk, the research participant will be constantly monitored and at any sign that the drug may be causing harm to health, the treatment will be immediately interrupted. Risks associated with blood collection include: pain, bruising, or other discomfort at the collection site. Rarely, fainting or puncture site infections may occur. To minimize discomfort, we will use the same intravenous access as the patient. The research participant may also feel uncomfortable during the quality of life questionnaire. The team of this project will be trained for the application of the same and will do everything to avoid any discomfort. During the CT or MRI scan, you may feel claustrophobic. We advise that if these discomforts occur, the research participant informs the doctor and nurse who will be present during the examination.

Benefits:

There are no direct benefits for the participant in this study. However, the results obtained may help in the treatment of epilepsy after TBI.

**Comments and Considerations on the Research:**

This amendment incorporates the following amendment:

• Research Protocol:

Main reason for change: Amendment generated to update the project team and protocol of research according to the points highlighted in protocol version 4 and also listed in table I of the letter of amendment. These changes

were made in the protocol aiming at a greater robustness in the final results. We also took the opportunity to insert a statement justifying the use of placebo and informing the exclusion of the participating center: Hospital de Base do Distrito Federal under the responsibility of the researcher Dr. Pedro Oliveira.

**Considerations on the Mandatory Submission Terms:**

Not applicable.

**Recommendations:**

There is not.

**Conclusions or Pending and List of Inadequacies:**

Amendment to the project registered in CEPesq as HSL 2020-175, APPROVED on this date according to the project presented.

- Research Protocol version 4.0 of October 2021

We remind you that, according to item XI.2.d of Res. 466/2012, the researcher must keep CEPesq informed about the progress of their research by sending partial (semestral) and final reports.

Final Considerations at the discretion of the CEP:

-

**This opinion was prepared based on the documents listed below**:

| Document type | File | Date | Post author | Status |
| --- | --- | --- | --- | --- |
| Basic information | PB_INFORMAÇÕES_BÁSICAS_184870 | 25/10/2021 |  | Accepted |
| from the project | 7_E2.pdf | 17:52:05 |  |  |
| Detailed project / | 27Biperideno_protocolov4_limpo.docx | 25/10/2021 | Carla Cristina Gomes | Accepted |
| Brochure |  | 17:44:13 | Pinheiro |  |
| Investigator |  |  |  |  |
| Detailed project / | 27Biperideno_protocolov4_limpo.pdf | 25/10/2021 | Carla Cristina Gomes | Accepted |
| Brochure |  | 17:43:50 | Pinheiro |  |
| Investigator |  |  |  |  |
| Detailed project / | 27Biperideno_protocolov4_altdestacada | 25/10/2021 | Carla Cristina Gomes | Accepted |
| Brochure Investigator | s.docx | 17:43:36 | Pinheiro |  |
| Detailed project /  Brochure Investigator | 27Biperideno_protocolov4_altdestacada  s.docx | 25/10/2021  17:43:36 | Carla Cristina Gomes  Pinheiro | Accepted |
| Detailed project /  Brochure Investigator | 27Biperideno_protocolov4_altdestacada s.pdf | 25/10/2021  17:43:14 | Carla Cristina Gomes Pinheiro | Accepted |
| Other | 27Biperideno_JustificativaPlacebo_v1.p  df | 25/10/2021  17:42:57 | Carla Cristina Gomes  Pinheiro | Accepted |
| Other | 27Biperideno_CartaEmenda_02.pdf | 25/10/2021  17:41:17 | Carla Cristina Gomes  Pinheiro | Accepted |
| Detailed project /  Brochure Investigator | 27Biperideno_protocolov3_altdestacada s_v04.docx | 21/06/2021  15:23:54 | Carla Cristina Gomes Pinheiro | Accepted |
| ICF / Terms of  Assent/Justification of Absence | 27Biperideno_TCLEv02_vf.docx | 21/06/2021  15:16:04 | Carla Cristina Gomes  Pinheiro | Accepted |
| Outros | 27Biperideno_CartaEmenda1.pdf | 21/06/2021  15:15:51 | Carla Cristina Gomes  Pinheiro | Accepted |
| Detailed project /  Brochure Investigator | 27Biperideno_protocolov3_vf.docx | 21/06/2021  15:14:19 | Carla Cristina Gomes Pinheiro | Accepted |
| Cronogram | 27Biperideno_Cronograma.docx | 01/06/2021  14:59:16 | Eliana Garzon | Accepted |
| Declaration of  researchers | 27Biperideno_compromissopesquisador  _v02.pdf | 08/10/2020  09:47:48 | Eliana Garzon | Accepted |
| Other | 27Biperideno_autorizacaoarea_v02.pdf | 07/10/2020  18:13:51 | Eliana Garzon | Accepted |
| Declaration of  Sponsor | 27Biperideno_parecerIEP_v01.pdf | 07/10/2020  18:13:32 | Eliana Garzon | Accepted |
| Cover sheet | 27Biperideno_FR_v02.pdf | 05/10/2020  12:37:19 | Eliana Garzon | Accepted |

**Opinion Status:**

Approved

**Requires CONEP Appraisal:**

No

SAO PAULO, November 08, 2021

# Signed by:

# Benedito Mauro Rossi (Coordinator)
