# Supplementary material for: Biperiden for prevention of post-traumatic epilepsy: A protocol of a double-blinded placebo-controlled randomized clinical trial (BIPERIDEN trial)
Source: PLoS One. 2022 Sep 9;17(9):e0273584. doi: 10.1371/journal.pone.0273584 (PMC9462738; doi:10.1371/journal.pone.0273584)
Supplement: S4 File — (PDF) [file pone.0273584.s005.pdf]

PARECER CONSUBSTANCIADO DO CEP

DADOS DA EMENDA

Título da Pesquisa: Biperideno para prevenção de epilepsia em pacientes com traumatismo crânio-encefálico.

Pesquisador: Eliana Garzon

Área Temática:

Versão: 3

CAAE: 39005920.8.1001.5461

Instituição Proponente: Sociedade Beneficente de Senhoras Hospital Sírio-Libanês

Patrocinador Principal: Sociedade Beneficente de Senhoras Hospital Sírio-Libanês

DADOS DO PARECER

Número do Parecer: 5.084.793

Apresentação do Projeto:

Desenho:

Ensaio clínico randomizado multicêntrico, duplo-cego, controlado por placebo, classificado como estudo clínico de fase 3. O planejamento do estudo seguirá as recomendações do Consolidated Standards of Reporting Trials (CONSORT Statement) [Schulz 2010], que apesar de ter sido desenvolvido para orientar o relato ou a publicação, tem sido amplamente utilizado também para orientar o planejamento de um ensaio clínico randomizado. O estudo será planejado e desenvolvido com o devido rigor a fim de minimizar a ocorrência dos viéses catalogados na literatura até o momento [de Vito 2019].O relato dos resultados do estudo seguirá o Consolidated Standards of Reporting Trials (CONSORT Statement) [Schulz 2010].

Resumo:

O traumatismo cranioencefálico (TCE) é um problema de saúde pública, com consideráveis custos socio-econômicos. O TCE é uma das causas mais importantes de epilepsia secundária (neste caso, chamada de epilepsia pós-traumática, EPT). A fisiopatologia da EPT decorre de lesões do parênquima cerebral que desencadeiam respostas neurodegenerativas e inflamatórias e levam a alterações moleculares, estruturais e eletrofisiológicas que acabam resultando na EPT. A conduta terapêutica indicada para o TCE pode envolver medicamentos e/ou procedimentos cirúrgicos, não havendo ainda qualquer intervenção terapêutica efetiva para diminuir a ocorrência de EPT.

Diversos estudos com modelos animais mostraram que drogas que modificam os processos de plasticidade neuronal, se administradas sob certas condições, tem o potencial de modificar o curso natural da EPT. Entre essas drogas, o biperideno (anti-colinérgico de uso clínico para Parkinson) diminuiu a incidência e intensidade de crises epilépticas espontâneas e retardou o aparecimento das mesmas em um modelo de epilepsia, mostrando ser um excelente candidato a agente antiepileptogênico. Pretende-se aqui testar a eficácia e segurança do uso do biperideno em pacientes adultos, vítimas de TCE moderados e graves. Os pacientes serão tratados ao longo de 10 dias após o trauma, objetivando evitar a formação do foco epileptogênico. Prospectivamente, os pacientes serão acompanhados em retornos periódicos ao longo de dois anos para avaliação de desenvolvimento de crises epilépticas. Também serão avaliados demais fatores que podem ter benefícios ou influenciar o tratamento, tais como marcadores genéticos e aspectos neuropsicológicos. Se positivo, esse tratamento: 1) caracteriza um mecanismo de ação; 2) abre caminho para o teste de novas moléculas e; 3) constitui uma opção de baixo custo (caracteriza-se como segundo uso de droga, disponível no SUS). Ademais, a presente proposta além de tipificar um clinical trial, busca entender os mecanismos básicos da EPT e de sua prevenção.

Introdução:

Traumatismo crânio-encefálicoDefinido como alteração da função cerebral ou evidência de patologia cerebral causada por uma força externa, o traumatismo cranioencefálico (TCE) é responsável por 1% de todas as mortes em adultos no mundo [Gentile 2011], o que corresponde a 50 milhões de pessoas ao redor do mundo todo ano, sendo a principal causa de mortalidade e comorbidade em todos os países, inclúsive no Brasil [Maas 2017; Ministério da Saúde 2015; Pereira 2006].Dados do DATASUS mostram que cerca de 125.000 pessoas/ano recebem atendimento hospitalar devido ao TCE, o que corresponde a uma frequência de 65,7 casos / 100.000 habitantes [Magalhães 2017]. Como preocupação adicional, a taxa de mortalidade hospitalar para estes casos chega a 7,7%, ou seja, 5,1 mortes /100.000 casos / ano [Magalhães 2017]. Os acidentes de trânsito representam a principal causa de TCE, totalizando 50% dos casos, seguidos por quedas e violência urbana [de Almeida 2016]. Estudos mostram que apesar do tamanho do impacto do TCE, evidências científicas fortes para suportar diretrizes e recomendações de tratamento ainda são escassas, com ensaios clínicos falhando em mostrar real eficácia de tratamento, apesar de resultados promissores [Maas 2017].Com relação à morbidade associada ao TCE, as complicações neurológicas incluem lesões cerebrais primárias (resultado direto do trauma) e secundárias (iniciadas após o momento do trauma, derivada dos danos primários) [Faul 2015; Masel 2010].

Sobreviventes ao TCE, frequentemente experenciam traumas físicos, pscicológicos, emocionais e cognitivos, o que pode causar uma redução importante na qualidade de vida e funcionalidade do indíviduo e traz consigo um alto impacto socioeconômico [Maas 2017].Epilepsia Pós-traumáticaA epilepsia pós-traumática (EPT) é uma complicação neurológica que ocorre em até 20% dos pacientes, com o risco aumentando de acordo com a severidade do TCE, intervenção cirurgica, intervalo de tempo desde o TCE, sendo que esse percentual sobe para 53% nos casos mais graves com lesões penetrantes [Annegers 1998; Asikainen 1999; Englander 2003; Hauser 1991; Kim 2018; Raymont 2010; Salazar 2015; Temkin 1990]. A EPT pode ser precoce (quando ocorre dentro de um a duas semanas após o trauma) ou tardia (após este período). As crises precoces podem ser causadas por efeitos agudos do trauma, como hemorragias e edema cerebral (e não necessariamente caracterizam epilepsia), enquanto as tardias parecem depender de mecanismos de reorganização sináptica [Payan 1970; Yablon 1993]A maioria das pessoas que desenvolve epilepsia secundária ao trauma craniano manifesta as crises epilépticas nos dois primeiros anos após o trauma ou lesão [da Silva 1990]. Assim como ocorre em outros tipos de epilepsia, as causas que ligam o TCE à EPT ainda não foram totalmente elucidadas e ainda não é possível evitar o processo da epileptogênese. Fomos incapazes de encontrar qualquer evidência forte de um tratamento específico para previnir ou reverter as crises epilépticas após TCE [Brady 2019]. Tratamento do TCE e prevenção da EPTA conduta terapêutica indicada para o TCE pode envolver vários medicamentos e/ou procedimentos cirúrgicos, para cuidados aos danos primários e secundários e depende em essência da extensão da lesão e de quais áreas foram acometidas. Avanços terapêuticos recentes envolvem medicamentos com alvos nos mecanismos dos danos secundários, incluindo bloqueadores de canais de cálcio, corticoesteroides, inibidores de aminoácidos excitatórios, antagonistas de receptor de N-metil D-aspartato (NMDA), “scavengers” de radicais livres, sulfato de magnésio, fatores de crescimento [Salazar 1985]. Ensaios clínicos em desenvolvimento utilizando diversas abordagens apresentam grande potencial terapêutico para TCE como eritropoetina, estatinas, células de medula óssea, progesterona. Ensaios clínicos recentes têm tido como foco estratégias neuroprotetoras, a fim de prevenir e/ou reduzir o dano cerebral secundário ao TCE [Temkin 1990]. No entanto, nenhuma dessas intervenções parece influenciar a ocorrência de EPT [French 2013; Klein 2017; Piccena 2017; Pitkänen 2010; Temkin 2001; Temkin 2003]. Substâncias anticonvulsivantes são indicadas para o controle das crises epilépticas que eventualmente ocorram de forma aguda, mas sua administração não evita a evolução para epilepsia [D’Ambrosio 2004]. Nesse contexto, não existem medicamentos neuroprotetores com indicação de evitar que os processos epileptogênicos se estabeleçam após

um dano cerebral, seja ele traumático, isquêmico, ou de outra natureza [Brady 2019; Temkin 2001]. Buscando resolver esta lacuna, estudos com modelos animais identificaram que medicamentos que alteram os processos de plasticidade neuronal, se administrados sob certas condições, tem o potencial de modificar o curso natural da EPT [Bittencourt 2017]. Especificamente com relação as condições referidas no estudo de Bittencourt, et al., [2017], encontra-se: a) a janela terapêutica, isto é o intervalo e tempo entre o evento lesivo (TCE) e a administração da primeira dose de biperideno (indicada em nosso projeto como devendo ser igual ou menor as 12 h); b) a duração da modulação do processo de plasticidade neuronal, isto é a duração do tratamento com biperideno (indicada em nosso protocolo como devendo ser de 10 dias); c) a dose de biperideno, de 5 mg a cada 6 horas. Evidências em estudos de fase pré-clínica indicaram que estas três condições acima devem ser atendidas para que o biperideno demonstre uma efetividade como agente capaz de demonstrar uma ação de modificação do curso natural da doença [Bittencourt 2017].Em estudos experimentais utilizando modelos de epilepsia, o biperideno, um anticolinérgico de uso clínico para Parkinson, mostrou ter ação na plasticidade neuronal, reduzindo a incidência e a intensidade de crises epilépticas espontâneas e retardando o aparecimento das mesmas [Bittencourt 2017; Gorgati 2009]. Outro estudo reportou o uso do biperideno como antídoto para o tratamento de distonia em crise não epiléptica decorrente de reação medicamentosa [Schwind & Antoniuk 2013]. Estes achados, somados à escassa literatura sobre o efeito do biperideno na prevenção de EPT após TCE ressaltam que o biperideno poderia ser uma potencial opção terapêutica no cuidado de pacientes com TCE, considerando sua ação anti-epileptogênica provável.

Hipótese:
Biperideno poderia ser uma potencial opção terapêutica no cuidado de pacientes com TCE, considerando sua ação anti-epileptogênica provável.

Metodologia Proposta:
Ensaio clínico randomizado multicêntrico, duplo-cego, controlado por placebo, classificado como estudo clínico de fase 3. P (população) = indivíduos com TCE agudo moderado a grave, com hemorragia intraparenquimatosa confirmada.I (intervenção) = biperidenoC (comparador) = placeboO (outcomes, desfechos) = desfechos de efetividade e segurança (detalhados adiante)IntervençãoGrupo biperidenoDentro do período de 12 horas após o TCE, os participantes do grupo biperideno receberão a dose de 5 mg (1 ml de volume total) de lactato de biperideno (Cinetol,

Cristália, Brasil), diluído em 10 mL de soro fisiológico 0,9% e aplicado por via intravenosa lentamente. O tratamento será repetido a cada 6 horas durante 10 dias consecutivos. Co-intervenções ao tratamento convencional de suporte para manejo do TCE recebido pelos participantes seguirá o protocolo do hospital participante, contudo, será recomendado pelo centro coordenador que os hospitais sigam as diretrizes do ministério da saúde para o tratamento de TCE [MS 2015] Será ainda perguntado no questionário de feasibility se o hospital faz uso destas diretrizes e ainda o material publicado pelo MS será encaminhado as equipes de pesquisa do centros participantes. Como a geração da sequência de alocação (descrita em detalhes adiante) será central e estratificada por centro participante, pelos preceitos deste tipo de randomização, espera-se que as diferenças das co -intervenções sejam distribuídas de modo semelhante entre os dois grupos, permitindo que qualquer efeito observado seja devido ao uso ou não do biperideno.

Critério de Inclusão:
Consentimento em participar fornecido pelo responsável, que deverá assinar e datar o TCLE após ser orientado sobre o estudo pelo investigador principal ou responsável, ler e concordar com a carta de informação; Idade entre 18 e 75 anos de idade; Ambos os sexos; Diagnóstico de TCE agudo moderado a grave; Escala de Coma de Glasgow (GCS) maior que 6 a 12 na admissão hospitalar; Presença de hemorragia intraparenquimatosa aguda e/ou contusão confirmada por ressonância magnética (RM) e/ou tomografia computadorizada (TC).

Critério de Exclusão:
Participantes vulneráveis, em situação de rua, sem documentação e sem endereço fixo e/ou contato de familiares; Pacientes sem documento e com idade duvidosa (18-75 anos) na admissão hospitalar; Estar em uso de biperideno no período de ocorrência do TCE; Histórico de epilepsia (confirmado por prontuário e/ou por uso de medicamento específico e/ou referido pelo responsável legal); Histórico de convulsões ou uso de medicação antiepiléptica; História de lesões perinatais, meningite e/ou encefalite (ou outro fator de risco comprovado ou provável para epilepsia); História de neoplasia, doenças neurodegenerativas; história de acidente vascular encefálico (AVE), disfunção cognitiva, hiperplasia prostática benigna, bloqueio atrioventricular ou qualquer outra arritmia cardíaca, ou glaucoma; Gestação; Participação atual em outro ensaio clínico; Portadores de arritmias cardíacas ou glaucoma, pelo risco aumentado de desenvolver reações adversas.
Metodologia de Análise de Dados:

Serão realizadas as seguintes análises:• análise intra-grupo: para avaliar o comportamento da variável ao longo do tempo dentro de um único braço de intervenção.• análise inter-grupos: para avaliar os desfechos de interesse, considerando a ocorrência dos eventos ou médias dos escores entre os dois braços de intervenção em todos os timepoints planejados). Estas serão as análises de maior interesse, pois por meio delas é que é possível comparar os efeitos de duas ou mais intervenções em um ECR.Para avaliar as características da distribuição dos dados (gaussiana ou não), serão aplicados os testes de Kolmogorov-Smirnov e Shapiro-Wilk. Para os testes serão utilizadas suas respectivas tabelas de valores críticos de acordo com a amostra e o nível de significância (valor assumido: 0,05) . Para o teste de Kolmogorv-Smirnov, se o valor calculado pelo teste for maior que o valor crítico, a hipótese de normalidade dos dados será rejeitada. Para o teste de Shapiro-Wilk, a hipótese de normalidade dos dados será rejeitada se o valor calculado for menor que o valor crítico.Para comparar os resultados médios obtidos entre os grupos (denominado efeito de grupo) e também, ao mesmo tempo, entre dois momentos (1, 3, 6, 9, 12, 18 e 24 meses), será aplicada a análise de variância (ANOVA) com medidas repetidas, considerando que as medidas ao longo do tempo são relacionadas ao mesmo paciente. Caso a distribuição dos dados não seja gaussiana, será utilizado o teste de Friedman, com o teste de Wilcoxon para análise post-hoc. Para as variáveis dicotômicas, será utilizado o teste qui-quadrado ou teste exato de Fisher (menos de cinco eventos em uma célula da tabela de contingência) para comparar a frequência de eventos entre os dois grupos de intervenção.Para todos os testes, será considerado um nível de significância de 5% e os dados serão analisados por intenção de tratar (ITT) e per protocol. Caso haja diferença nos resultados encontrados com as duas análises, os resultados da análise ITT serão considerados como principais. Em caso de perdas de dados ou de participantes, as análises ITT serão realizadas utilizando métodos apropriados para imputação dos dados faltantes (last observation carried forward - LOCF, média dos participantes remanescentes ou abordagens mistas). Para as variáveis dicotômicas, será utilizado o teste qui-quadrado ou teste exato de Fisher para comparar a frequência de eventos entre os dois grupos de intervenção.Para todos os testes, será considerado um nível de significância de 5% e os dados serão analisados por intenção de tratar (ITT) e per protocol. Caso haja diferença nos resultados encontrados com as duas análises, os resultados da análise ITT serão considerados como principais. Em caso de perdas de dados ou de participantes, as análises ITT serão realizadas utilizando métodos apropriados para imputação dos dados faltantes (last observation carried forward - LOCF, média dos participantes remanescentes ou abordagens mistas).

Desfecho Primário:
Primários Eficácia: Epilepsia pós-traumática: avaliada pela proporção de participantes que desenvolveram EPT no período compreendido entre sete dias e 24 meses após o TCE. A presença de EPT deverá ser confirmada clinicamente. EPT será definida como a ocorrência de pelo menos duas crises não provocadas, ocorrendo em um período superior a sete dias pós TCE [Verellen 2010].Segurança: Eventos adversos graves: proporção de participantes que apresentaram pelo menos um evento adverso grave após 24 meses da inclusão no estudo. Os eventos considerados graves são definidos como aqueles que resultam em morte, ameaçam a vida, requerem internação hospitalar ou prolongamento da hospitalização existente, resultam em incapacidade (incapacidade persistente ou significativa), ou anomalias congênitas (defeitos de nascimento), suspeita de transmissão de agente infeccioso por meio de medicamento [Anvisa 2016]. As informações referentes a ocorrência deste desfecho serão compiladas no formulário padronizado individual de cada participante, que será preenchido pela equipe de pesquisa ao longo do projeto a partir de interrogatório ativo direto aos participantes e também a partir de relatos espontâneos dos mesmos.

Desfecho Secundário:
Eficácia: Epilepsia pós-traumática: avaliada pela proporção de participantes que desenvolveram EPT nos seguintes momentos de avaliação: 1, 3, 6, 9, 12, e 18 meses após a inclusão no estudo. Qualidade de vida: avaliada pela ferramenta EuroQoL 5D (EQ-5D) [EuroQoL Group 1990] nos seguintes momentos de avaliação: 3, 6, 12, e 24 meses após a inclusão no estudo. O EQ-5D é um questionário validado, com versão validada em português, abrangendo cinco domínios de qualidade de vida relacionada à saúde (mobilidade, cuidados usuais, atividades habituais, dor/mal-estar, ansiedade/depressão). Função neuropsicológica: serão aplicados testes que indiquem a medida geral da inteligência, atenção, memória auditivoverbal e visual, memória operacional, destreza visual-motora e flexibilidade cognitiva. Os seguintes instrumentos serão aplicados após 6 e 24 meses da inclusão no estudo: Itens da Bateria da Escala Wechsler de Inteligência - IV [Wechlsler 2008]: Dígitos: O sujeito deve repetir, oralmente, uma série de sequências numéricas apresentadas em ordem direta e inversa. Avalia a memória imediata e a memória operacional. Vocabulário: Deve ser explicado verbalmente o significado de cada uma das 33 palavras apresentadas oralmente ao sujeito. Avalia o desenvolvimento da linguagem, conhecimento de palavras e memória de longo prazo. Cubos: Reproduzir, com cubos bidimensionais e bicolores, os modelos apresentados como padrões. Avalia a percepção visual de

estímulo abstrato, organização espacial, planejamento, coordenação visomotora, análise e síntese. Dígitos e Símbolos: o sujeito deve preencher sob pressão de tempo uma folha contendo símbolos, associando-os a dígitos de acordo com um modelo apresentado. A aplicação tem duração aproximada de 60 minutos, o que para o paciente que acaba de sair da internação hospitalar pode ser demorada, mas ao longo da recuperação vão se tornando viáveis. É necessário um intervalo mínimo de 6 meses entre as aplicações para que não ocorra efeito de aprendizagem. Figura complexa de Rey-Osterrieth: o sujeito é solicitado a copiar uma figura complexa e, após 5 minutos e sem o modelo, redesenhar de memória. Permite avaliar as habilidades de organização visuo-espacial, planejamento e desenvolvimento de estratégias, bem como memória [Oliveira 1999]. Teste do Aprendizado Auditivo-Verbal de Rey. O sujeito é solicitado a repetir uma lista de 15 palavras lida em voz alta pelo examinador. O procedimento é repetido 5 vezes. Após isto é introduzida uma segunda lista (estímulo distrator). Em seguida, o sujeito é solicitado a dizer a lista original de memória. Após 15 minutos, nova recordação é solicitada [Rey 1941]. Teste dos Cinco Dígitos: investiga a flexibilidade cognitiva e o controle inibitório. O sujeito é solicitado, de modo alternado, a: (a) contar o número de estímulos apresentados e (b) dizer os dígitos impressos [Sedo 2005]. Padrão eletroencefalográfico (EEG): avaliado pelos traçados realizados logo após o TCE em 1, 3, 6, 9, 12, 18 e 24 meses. Pesquisa de Apolipoproteína E (ApoE), considerado fator prognóstico importante para o desenvolvimento de sequelas neurológicas póstraumáticas. O material biológico para a análise será coletado por swab oral ou sangue periférico, no momento da internação hospitalar.Segurança: Mortalidade geral: avaliada pela proporção de participantes que morreram após término do período de seguimento de dois anos de todos os participantes. Eventos adversos não-graves: avaliados pela proporção de participantes que apresentaram pelo menos um evento adverso não-grave nos seguintes momentos de avaliação: 1, 3, 6, 9, 12, 18 e 24 meses após a inclusão no estudo. As informações referentes a ocorrência deste desfecho serão compiladas no formulário padronizado individual de cada participante, que será preenchido pela equipe de pesquisa ao longo do projeto a partir de interrogatório ativo direto aos participantes e também a partir de relatos espontâneos dos mesmos.

Objetivo da Pesquisa:

Objetivo Primário:

Avaliar os efeitos (benefícios e riscos) e a custo-efetividade do uso de biperideno para prevenção de epilepsia pós-traumática (EPT) em pacientes com traumatismo crânio-encefálico (TCE) e hemorragia intraparenquimatosa confirmada.

Objetivo Secundário:
1. Avaliar a efetividade do biperideno para prevenção de EPT.
2. Avaliar a segurança do biperideno para prevenção de EPT.3. Avaliar a custo-efetividade do biperideno para prevenção de EPT.

Avaliação dos Riscos e Benefícios:
Riscos:
Nesta pesquisa os riscos são mínimos, contudo, ressaltamos alguns pontos que podem gerar algum desconforto. O Biperideno já é um medicamento aprovado e utilizado no tratamento de pacientes com Doenças de Parkinson. Todo medicamento possui risco em sua utilização. Para diminuir este risco o participante de pesquisa será constantemente monitorado e a qualquer sinal de que o medicamento possa estar causando mal à saúde o tratamento será imediatamente interrompido. Riscos associados com a coleta de sangue incluem: dor, hematoma, ou outro desconforto no local da coleta. Raramente desmaio ou infecções no local de punção podem ocorrer. Para minimizar os desconfortos utilizaremos o mesmo acesso intravenoso do paciente. O participante de pesquisa também pode se sentir desconfortável durante a realização do questionário de qualidade de vida. A equipe deste projeto será treinada para a aplicação do mesmo e fará de tudo para que qualquer desconforto seja evitado. Durante a realização de tomografia computadorizada ou ressonância magnética pode se ter a sensação de claustrofobia. Orientamos que se estes desconfortos ocorram que o participante de pesquisa comunique o médico e enfermeiro que estarão presente durante a realização deste exame.

Benefícios:
Não há benefícios diretos para o participante deste estudo. Contudo, os resultados obtidos poderão auxiliar no tratamento de epilepsia após TCE.

Comentários e Considerações sobre a Pesquisa:
Esta emenda incorpora a seguinte alteração:

• Protocolo de Pesquisa:

Principal razão para alteração: Emenda gerada para atualizarmos a equipe do projeto e o protocolo

de pesquisa conforme os pontos destacados no protocolo versão 4 e também listados na tabela I da carta de emenda. Estas mudanças foram feitas no protocolo visando uma maior robustez nos resultados finais. Aproveitamos ainda a oportunidade para inserir uma declaração de justificativa do uso de placebo e informar a exclusão do centro participante: Hospital de Base do Distrito Federal sob responsabilidade do pesquisador Dr. Pedro Oliveira.

Considerações sobre os Termos de apresentação obrigatória:

Não se aplica.

Recomendações:

Não há.

Conclusões ou Pendências e Lista de Inadequações:

Emenda do projeto cadastrado no CEPesq como HSL 2020-175, APROVADA nesta data conforme o projeto apresentado.

- Protocolo de Pesquisa versão 4.0 de outubro de 2021

Lembramos que, conforme o item XI.2.d da Res. 466/2012 o pesquisador deverá manter o CEPesq informado sobre o andamento de sua pesquisa através do envio de relatórios parciais (semestrais) e final.

Considerações Finais a critério do CEP:

-

Este parecer foi elaborado baseado nos documentos abaixo relacionados:

Tipo Documento	Arquivo	Postagem	Autor	Situação
Informações Básicas do Projeto	PB_INFORMAÇÕES_BÁSICAS_1848707_E2.pdf	25/10/2021 17:52:05		Aceito
Projeto Detalhado / Brochura Investigador	27Biperideno_protocolov4_limpo.docx	25/10/2021 17:44:13	Carla Cristina Gomes Pinheiro	Aceito
Projeto Detalhado / Brochura Investigador	27Biperideno_protocolov4_limpo.pdf	25/10/2021 17:43:50	Carla Cristina Gomes Pinheiro	Aceito
Projeto Detalhado / Brochura	27Biperideno_protocolov4_altdestacadas.docx	25/10/2021 17:43:36	Carla Cristina Gomes Pinheiro	Aceito

Endereço: Rua Barata Ribeiro nº 269
Bairro: Bela Vista **CEP:** 01.308-000
UF: SP **Município:** SAO PAULO
Telefone: (11)3394-5701 **E-mail:** cepesq@hsl.org.br

Investigador	27Biperideno_protocolov4_altdestacadas.docx	25/10/2021 17:43:36	Carla Cristina Gomes Pinheiro	Aceito
Projeto Detalhado / Brochura Investigador	27Biperideno_protocolov4_altdestacadas.pdf	25/10/2021 17:43:14	Carla Cristina Gomes Pinheiro	Aceito
Outros	27Biperideno_JustificativaPlacebo_v1.pdf	25/10/2021 17:42:57	Carla Cristina Gomes Pinheiro	Aceito
Outros	27Biperideno_CartaEmenda_02.pdf	25/10/2021 17:41:17	Carla Cristina Gomes Pinheiro	Aceito
Projeto Detalhado / Brochura Investigador	27Biperideno_protocolov3_altdestacadas_v04.docx	21/06/2021 15:23:54	Carla Cristina Gomes Pinheiro	Aceito
TCLE / Termos de Assentimento / Justificativa de Ausência	27Biperideno_TCLEv02_vf.docx	21/06/2021 15:16:04	Carla Cristina Gomes Pinheiro	Aceito
Outros	27Biperideno_CartaEmenda1.pdf	21/06/2021 15:15:51	Carla Cristina Gomes Pinheiro	Aceito
Projeto Detalhado / Brochura Investigador	27Biperideno_protocolov3_vf.docx	21/06/2021 15:14:19	Carla Cristina Gomes Pinheiro	Aceito
Cronograma	27Biperideno_Cronograma.docx	01/06/2021 14:59:16	Eliana Garzon	Aceito
Declaração de Pesquisadores	27Biperideno_compromissopesquisador_v02.pdf	08/10/2020 09:47:48	Eliana Garzon	Aceito
Outros	27Biperideno_autorizacaoarea_v02.pdf	07/10/2020 18:13:51	Eliana Garzon	Aceito
Declaração do Patrocinador	27Biperideno_parecerIEP_v01.pdf	07/10/2020 18:13:32	Eliana Garzon	Aceito
Folha de Rosto	27Biperideno_FR_v02.pdf	05/10/2020 12:37:19	Eliana Garzon	Aceito

Situação do Parecer:
Aprovado

Necessita Apreciação da CONEP:
Não

SAO PAULO, 08 de Novembro de 2021

Assinado por:
Benedito Mauro Rossi
(Coordenador(a))
