# Supplementary material for: Biperiden for prevention of post-traumatic epilepsy: A protocol of a double-blinded placebo-controlled randomized clinical trial (BIPERIDEN trial)
Source: PLoS One. 2022 Sep 9;17(9):e0273584. doi: 10.1371/journal.pone.0273584 (PMC9462738; doi:10.1371/journal.pone.0273584)
Supplement: Attachment — Submitted filename: PONE-D-22-16132_reviewer.pdf [file pone.0273584.s006.pdf]

PLOS ONE

# Biperiden for prevention of post-traumatic epilepsy: a protocol of a double-blinded placebo-controlled randomized clinical trial (BIPERIDEN trial)

--Manuscript Draft--

| | |
|---|---|
| Manuscript Number: | PONE-D-22-16132 |
| Article Type: | Study Protocol |
| Full Title: | Biperiden for prevention of post-traumatic epilepsy: a protocol of a double-blinded placebo-controlled randomized clinical trial (BIPERIDEN trial) |
| Short Title: | Biperiden Trial for Epilepsy Prevention |
| Corresponding Author: | Luiz Eugenio Mello, M.D., Ph.D<br>Universidade Federal de Sao Paulo<br>Sao Paulo, SP BRAZIL |
| Keywords: | Traumatic Brain Injury, Epilepsy, Biperiden, Antiepileptogenic, Anticholinergic drugs, Acetylcholine, Randomized double-bind controlled clinical trial. |
| Abstract: | Background:  Traumatic brain injury (TBI) is one of the most important causes of acquired structural epilepsy, post-traumatic epilepsy (PTE), however, efficient preventative measures and treatment are still not available to patients. Preclinical studies indicated biperiden, an anticholinergic drug, as a potential drug to modify the epileptogenic process. The main objective of this clinical trial is to evaluate the efficacy of biperiden as an antiepileptogenic agent in patients that suffered TBI.<br>Methods:  This prospective multicenter (n = 10) interventional study will include 312 adult patients admitted to emergency care units with a diagnosis of moderate or severe TBI. Following inclusion and exclusion criteria, patients will be randomized, using block randomization, to receive double-blind treatment with placebo or biperiden for 10 days. Follow-up will occur at specific time windows up to 2 years. Main outcomes are incidence of PTE after TBI and occurrence of severe adverse events. Other outcomes include exploratory investigation of factors that might have benefits for the treatment or might influence its results, such as genetic background, clinical progression, electroencephalographic abnormalities, health-related quality of life and neuropsychological status. Analyses will be conducted following the safety, intention-to-treat and efficacy concepts.<br>Discussion:  We hypothesize that biperiden treatment will be effective to prevent or mitigate the development of post-traumatic epilepsy in TBI patients. Other health measures from this population also may benefit from treatment with biperiden.<br>Trial registration ClinicalTrials.gov, NCT04945213. Registered on June 30, 2021 |
| Order of Authors: | Maira Licia Foresti |
| | Eliana Garzon |
| | Carla Cristina Gomes Pinheiro |
| | Rafael Leite Pacheco |
| | Rachel Riera |
| | Luiz E Mello |
| Additional Information: | |
| Question | Response |
| Financial Disclosure<br><br>Enter a financial disclosure statement that describes the sources of funding for the work included in this submission. Review the submission guidelines for detailed requirements. View published research | The study is funded by Programa de Apoio ao Desenvolvimento Institucional do Sistema Único de Saúde (PROADI-SUS, Brazil) (NUP 25000.014325/2021-33). LEM has a grant from FAPESP (18/24561-5) and from CNPq (311619/2019-3). The funders had and will not have a role in study design, data collection and analysis, decision to publish, or preparation of the manuscript. |

Powered by Editorial Manager® and ProduXion Manager® from Aries Systems Corporation

| | |
|---|---|
| articles from *PLOS ONE* for specific examples.<br><br>This statement is required for submission and **will appear in the published article** if the submission is accepted. Please make sure it is accurate.<br><br>**Unfunded studies**<br>Enter: *The author(s) received no specific funding for this work.*<br><br>**Funded studies**<br>Enter a statement with the following details:<br>• Initials of the authors who received each award<br>• Grant numbers awarded to each author<br>• The full name of each funder<br>• URL of each funder website<br>• Have or will the sponsors or funders play any role in the study design, data collection (when applicable) and analysis, decision to publish, or preparation of the manuscript?<br>• **NO** - Include this sentence at the end of your statement: *The funders had and will not have a role in study design, data collection and analysis, decision to publish, or preparation of the manuscript.*<br>• **YES** - Specify the role(s) played.<br><br>* typeset | |
| **Competing Interests**<br><br>Use the instructions below to enter a competing interest statement for this submission. On behalf of all authors, disclose any competing interests that could be perceived to bias this work—acknowledging all financial support and any other relevant financial or non-financial competing interests.<br><br>This statement is required for submission and **will appear in the published article** if the submission is accepted. Please make sure it is accurate and that any funding sources listed in your Funding Information later in the submission form are also declared in your Financial Disclosure statement. | The authors have declared that no competing interests exist. |

| | |
|---|---|
| View published research articles from *PLOS ONE* for specific examples.<br><br>**NO authors have competing interests**<br><br>Enter: *The authors have declared that no competing interests exist.*<br><br>**Authors with competing interests**<br><br>Enter competing interest details beginning with this statement:<br><br>*I have read the journal's policy and the authors of this manuscript have the following competing interests: [insert competing interests here]*<br><br>* typeset | |
| **Ethics Statement**<br><br>Enter an ethics statement for this submission. This statement is required if the study involved:<br><br>• Human participants<br>• Human specimens or tissue<br>• Vertebrate animals or cephalopods<br>• Vertebrate embryos or tissues<br>• Field research<br><br>Write "N/A" if the submission does not require an ethics statement.<br><br>General guidance is provided below. Consult the submission guidelines for detailed instructions. **Make sure that all information entered here is included in the Methods section of the manuscript.** | The BIPERIDEN study has been approved by the Comitê de Ética em Pesquisa of Sociedade Beneficente de Senhoras, Hospital Sírio-Libanês (CAAE No. 39005920.8.1001.5461). Insurance has been taken out for participants of this study to cover damage due to participation. Written informed consent will be obtained from all study participants. |

| | |
|---|---|
| **Format for specific study types**<br><br>**Human Subject Research (involving human participants and/or tissue)**<br>• Give the name of the institutional review board or ethics committee that approved the study<br>• Include the approval number and/or a statement indicating approval of this research<br>• Indicate the form of consent obtained (written/oral) or the reason that consent was not obtained (e.g. the data were analyzed anonymously)<br><br>**Animal Research (involving vertebrate animals, embryos or tissues)**<br>• Provide the name of the Institutional Animal Care and Use Committee (IACUC) or other relevant ethics board that reviewed the study protocol, and indicate whether they approved this research or granted a formal waiver of ethical approval<br>• Include an approval number if one was obtained<br>• If the study involved *non-human primates*, add *additional details* about animal welfare and steps taken to ameliorate suffering<br>• If anesthesia, euthanasia, or any kind of animal sacrifice is part of the study, include briefly which substances and/or methods were applied<br><br>**Field Research**<br><br>Include the following details if this study involves the collection of plant, animal, or other materials from a natural setting:<br>• Field permit number<br>• Name of the institution or relevant body that granted permission | |
| **Data Availability**<br><br>Authors are required to make the data underlying their research findings fully available, without restriction.<br><br>If the manuscript reports pilot or preliminary results, the data underlying those results must be made available at the time of publication. A Data Availability | N/A - the protocol does not report results |

Powered by Editorial Manager® and ProduXion Manager® from Aries Systems Corporation

| | |
|---|---|
| Statement describing where the data can be found is required at submission. Your answers to this question constitute the Data Availability Statement and will be published in the article, if accepted. When legal or ethical requirements prohibit public data sharing, authors must indicate how researchers may obtain access to the data. See the PLOS Data Policy and FAQ for detailed information.<br><br>**Important:** Stating 'data available on request from the author' is not sufficient. If your data are only available upon request, select 'No' for the first question and explain your situation in the text box.<br><br>For protocols without pilot or preliminary data, authors are strongly encouraged to state how they plan to share research data from their study when it is completed or published.<br><br>Do the authors confirm that EITHER the protocol does not report results OR all data underlying the preliminary results will be made fully available without restriction? | |
| **Describe where the data may be found in full sentences. If you are copying our sample text, replace any instances of XXX with the appropriate details.**<br><br>• If **no pilot data are reported** in the manuscript, please enter the following: *No datasets were generated or analysed during the current study. All relevant data from this study will be made available upon study completion.*<br>• If the manuscript reports pilot data **held in a public repository**, include URLs, accession numbers or DOIs. If this information will only be available after acceptance, indicate this by ticking the box below. For example: *All XXX files are available from the XXX database* | Deidentified research data will be made publicly available when the study is completed and published from the<br>corresponding author upon reasonable request. |

Powered by Editorial Manager® and ProduXion Manager® from Aries Systems Corporation

| | |
|---|---|
| *(accession number(s) XXX, XXX.).*<br>• If the manuscript reports pilot data contained **within the manuscript and/or Supporting Information files**, enter the following:<br>*All relevant data are within the manuscript and its Supporting Information files.*<br>• If neither of these applies but you are able to provide **details of access elsewhere**, with or without limitations, please do so. For example:<br><br>*Data cannot be shared publicly because of [XXX]. Data are available from the XXX Institutional Data Access / Ethics Committee (contact via XXX) for researchers who meet the criteria for access to confidential data.*<br><br>*The data underlying the results presented in the study are available from (include the name of the third party and contact information or URL).*<br>• This text is appropriate if the data are owned by a third party and authors do not have permission to share the data.<br><br>For study protocols with data management or sharing plans, authors are encouraged to briefly describe in their Data Availability Statement how data they generate will be made accessible when the study is completed. For example:<br>• Deidentified research data will be made publicly available when the study is completed and published.<br><br>* typeset | |
| Additional data availability information: | |

Powered by Editorial Manager® and ProduXion Manager® from Aries Systems Corporation

Manuscript
Click here to access/download;Manuscript;Biperiden_Protcol_submiss_Paper

# Biperiden for prevention of post-traumatic epilepsy: a protocol of a double-blinded placebo-controlled randomized clinical trial (BIPERIDEN trial).

Maira Licia Foresti[1,2,3,¶], Eliana Garzon[2,4, ¶], Carla Cristina Gomes Pinheiro[2], Rafael Leite Pacheco[5,6], Rachel Riera[6,7] and Luiz Eugênio Mello[1,2,3*]

[1]Neurology Neuroscience Postgraduation Program, Physiology Department, Universidade Federal de São Paulo, São Paulo;

[2]Sociedade Beneficente de Senhoras Hospital Sírio-Libanês, São Paulo, SP, Brazil.

[3]Instituto D'Or de Pesquisa e Ensino, São Paulo, SP, Brasil.

[4]Department of Neurology, Division of Neurologic Clinic, Hospital das Clínicas, Faculdade de Medicina, Universidade de São Paulo, São Paulo-SP Brazil.

[5]Departamento de Medicina, Centro Universitário São Camilo, São Paulo, SP, Brazil;

[6]Center of Health Technology Assessment, Sociedade Beneficente de Senhoras Hospital Sírio-Libanês, São Paulo, SP, Brazil.

[7]Discipline of Evidence-based Medicine, Escola Paulista de Medicina, Universidade Federal de São Paulo, São Paulo, SP, Brazil;

* *Corresponding author:*

E-mail: lemello@unifesp.br (LEM)

[¶] These authors contributed equally to the paper

# Abstract

*Background:* Traumatic brain injury (TBI) is one of the most important causes of acquired structural epilepsy, post-traumatic epilepsy (PTE), however, efficient preventative measures and treatment are still not available to patients. Preclinical studies indicated biperiden, an anticholinergic drug, as a potential drug to modify the epileptogenic process. The main objective of this clinical trial is to evaluate the efficacy of biperiden as an antiepileptogenic agent in patients that suffered TBI.

*Methods:* This prospective multicenter (n = 10) interventional study will include 312 adult patients admitted to emergency care units with a diagnosis of moderate or severe TBI. Following inclusion and exclusion criteria, patients will be randomized, using block randomization, to receive double-blind treatment with placebo or biperiden for 10 days. Follow-up will occur at specific time windows up to 2 years. Main outcomes are incidence of PTE after TBI and occurrence of severe adverse events. Other outcomes include exploratory investigation of factors that might have benefits for the treatment or might influence its results, such as genetic background, clinical progression, electroencephalographic abnormalities, health-related quality of life and neuropsychological status. Analyses will be conducted following the safety, intention-to-treat and efficacy concepts.

*Discussion:* We hypothesize that biperiden treatment will be effective to prevent or mitigate the development of post-traumatic epilepsy in TBI patients. Other health measures from this population also may benefit from treatment with biperiden.

Trial registration ClinicalTrials.gov, NCT04945213. Registered on June 30, 2021

**Keywords:** Traumatic Brain Injury, Epilepsy, Biperiden, Antiepileptogenic, Anticholinergic drugs, Acetylcholine, Randomized double-bind controlled clinical trial.

# Introduction

Traumatic brain injury (TBI) is one of the main leading causes of death and lifelong disability in the world. One of the most important neurological consequences following TBI is the development of post traumatic epilepsy (PTE), which is responsible for approximately 5% of all focal epilepsy etiologies in the general population [1]. TBI patients present a higher risk to develop epilepsy than the general population, with 10-20% cumulative probability in 5 years [2, 3, 4]. The type and severity of injury correlates with the likelihood for a person to develop epilepsy, varying from 2.9% for moderate TBI to 17-27% for those who had severe TBI [2, 5, 6, 7]. Importantly, risk of epilepsy reaches 50% for patients with penetrating head injury [8]. Besides the high incidence, the causative links between TBI and epilepsy are still not completely understood, and there is currently no clinically available treatment or strategy that influences the likelihood of PTE development after brain injury [9].

Preclinical studies showed that anticholinergic drugs have the potential to modify neural plastic processes hampering epileptogenesis in animal models of epilepsy [10]. Among these drugs, biperiden, a cholinergic antagonist acting in the muscarinic receptor, administered soon after brain injury, delayed the latency and decreased the incidence and intensity of spontaneous seizures after brain injury [11]. These results were recently corroborated by other groups [12].

As a subsequent step, the use of biperiden in the acute phase after brain injury showed to be safe in a small phase II clinical study, which also served to test the feasibility of an interventional and prospective trial [10]. Here, the present Phase III study was designed to evaluate the safety and efficacy of biperiden in preventing the development of PTE in adult patients, who have suffered moderate or severe TBI, in a larger multicenter prospective study. Other clinically relevant neurological outcomes due biperiden treatment will also be investigated. The aim of this study is to evaluate the efficacy and safety of biperiden in preventing the development of epilepsy in patients that suffered TBI.

# Materials and Methods

It is a multicentric phase 3 randomized, double-blind clinical trial coordinated by Sociedade Beneficente de Senhoras, Hospital Sirio-Libanês (Brazil), throught a partnership with the Brazilian Ministry of Health and funded by the Programa de Apoio ao Desenvolvimento Institucional do Sistema Único de Saúde (PROADI-SUS, Brazil). The study was prospectively registered in ClinicalTrials.gov (NCT04945213). This reporting followed the recommendations of the Standard Protocol Items: Recommendations for Interventional Trials (SPIRIT Statement) [13] and the Template for Intervention Description and Replication (TIDieR-placebo) checklist [14].

Table 1 presents the study plan including the schedule of enrolment, intervention, and assessments. A complete SPIRIT checklist for clinical trial can be found on the Supporting Information file S1.

- *Table 1 presented in the end of the text should appear here after production* -.

# Ethical considerations

This study follows the principles of the Good Clinical Practice (GCP – ICH6 R2) in Randomized Controlled Trial (RCT). The whole protocol has been reviewed and approved by the Research Ethics Committee of Sociedade Beneficente de Senhoras, Hospital Sírio-Libanês (CAAE No. 39005920.8.1001.5461). Because the emergency nature of TBI, the Research Ethics Committee allowed to initiate the study protocol without informed consent of patients (or relatives), which must be provided until 48h.

An external Data Monitoring Committee (DMC) formed by three experienced clinic-researchers will provide independent assessment of the continuity of the study considering the safety, validity, ethical and scientific merit of the trial.

# Participants

The study will enroll adults (18 to 75 years) of both sex who fulfill the following inclusion criteria: meet the moderate or severe TBI criteria according with the Glasgow Coma Scale (GCS) [15], in such a way that GCS is between 6 and 12 at the accident scene and/or between 3 (if sedated) and 12 at hospital admission; have

computerized tomography (CT) scan with evidence of acute intraparenchymal hemorrhage and/or contusion; able to receive the first dose of treatment or placebo within 12 hours of brain injury, regardless of the accident cause. Participants, or their relatives in the event of patient´s unconsciousness, will have to provide written informed consent within 48 hours from the beginning of the protocol. Exclusion criteria are: current use of biperiden or antiseizure drugs; history of epilepsy, presence of other conditions that could increase risk factors for epilepsy development, such as perinatal injuries, meningitis, encephalitis, neoplasia, acute stroke, neurodegenerative diseases; any condition precluding the use of biperiden such as pregnancy, glaucoma, benign prostatic hyperplasia, atrioventricular block or cardiac arrhythmias; megacolon or mechanical obstruction of the gastrointestinal tract and inability to perform follow-up visits (e.g. homeless patients); refusal to participate in the study and current enrollment in other clinical trial. Alcohol intoxication or other psychotropic drug use at hospital admission will not lead to protocol exclusion.

We intend to recruit participants with acute TBI from at least ten hospitals located at different regions from Brazil (North, South, Southeast, Midwest and Northeast). After a first screening at the emergency care unit (ECU) for confirming eligibility criteria (which requires standard CT scan for acute TBI cases, electrocardiogram and pregnancy test for safety), those who fulfill all criteria will be included in the study. Because the emergency scenario and the injury severity, when the participant is not able to inform his medical history and is not accompanied by an appropriate informant, consent to participate and exclusion criteria might be confirmed within two days of protocol beginning. Participants will be prospectively monitored at their respective neurology/neurosurgery centers.

## Randomization and allocation concealment

Patients will be randomly assigned to receive placebo or biperiden using a random table generated by STATA® v17 software, in a 1:1 rate, in random permuted blocks and considering the recruiting centers as a

criterion for stratification. The allocation concealment will be assured using the REDCap (Research Electronic Data Capture), a web-based software platform [16, 17].

## Blinding procedures

The intervention and the placebo will be identical in appearance (color, volume and packaging). Participants, personnel and outcome assessor will be blinded to participant assignments.

## Interventions

Within 12 h after TBI, participants will receive intravenous (i.v.) infusions of 5 mg of biperiden (1 mL; Cinetol, Cristália, Brazil) diluted in 100 mL of 0.9% saline (treatment group) or 1 mL of sterile vehicle (sodium lactate, lactic acid, sodium hydroxide and water for injections) diluted in 100 mL of 0,9% saline (placebo group), every 6 hours for 10 days after TBI, until completing 40 total doses.

Patients will receive all medical treatment indicated for their clinical condition and will not be deprived of any other required treatment because of their participation in this study. Patients can be discontinued from the protocol if they remove the consent to participate; in any health conditions that health care providers, with sponsor agreement, believe that it is to the advantage of the patient not to comply with the procedures specified in this protocol; in case of screening error when including patients with history of epilepsy predicted in the study exclusion criteria (when this finding cannot occur at the time of recruitment).

During the hospitalization period, blood samples will be collected to analyze the genetic profile of patients regarding the presence of the polymorphic ε4 allele of the apolipoprotein E gene (APOEε4). Laboratory analyses will be performed by a central laboratory (DASA, São Paulo, Brazil), which is accredited by the College of American Pathologists (CAP, Northfield, IL, USA). Additional patients' information, such as occurrence of adverse events, sociodemographic data, occurrence of symptomatic acute seizures, unprovoked epileptic seizures starting 7 days after TBI and clinical evolution will be collected during the hospitalization period. Suspected and

diagnosed severe adverse events will be reviewed and adjudicated by the Ethics Committee and the external Data Monitoring Committee.

## Follow-up

At hospital discharge patients and their relatives will be asked to keep a diary of epileptic seizures and record all ictal events with detailed descriptions. Prospectively, patients will be followed up for two years, on periodic visits at 1, 3, 6, 9, 12, 18 and 24 months after TBI with a neurologist or neurosurgeon, in neurology centers, to assess the development of epileptic seizures. Specifically, seizure diary, clinical and neurological evaluation, which include the Portuguese version of the modified Rankin Scale (mRS) for neurologic disability [18], will be assessed. In case of prolonged hospitalization, follow up visits will occur at the hospital, as possible. Due to the COVID-19 pandemic, we anticipate performing some of these evaluations through telephone calls. Despite the limited clinical assessment promoted by this resource (e.g. loss of physical evaluation), it allows investigation over seizure occurrence.

Patients will be further monitored with electroencephalogram (EEG) at the same time points defined for clinical evaluation. EEG recordings will be analyzed by experienced neurophysiologists. Other personal aspects that might benefit from the treatment or might influence its results will also be evaluated on specific timepoints, as shown on Table 1, which include: social data and quality of life assessed using the Portuguese version of EuroQol three-level version (EQ-5D-3L) and EuroQol visual analogue scale (EQ VAS) at 3, 6, 12 and 24 months after TBI; standard neuropsychologic tests, including test batteries (Vocabulary, Block design, Digit Symbol-Coding, Symbol Search and Digit Span) of the Wechsler Intelligence Scale III (WAIS III); Rey–Osterrieth complex figure; Rey Auditory Verbal Learning Test (RAVLT) and Five Digit Test (FDT) which will be applied by psychologists at 6 months and then 24 months after TBI.

Data will be collected in an electronic case report form (eCRF) using free web-based software platform REDCap. Data entry will be monitored by an independent researcher according to a predefined monitoring plan.

Patient confidentiality will be ensured by using identification numbers. All records will be kept on file at the trial sites or the coordinating data center for 5 years. The frozen trial database file will be kept on file for 5 years.

## Outcomes

The primary and secondary outcomes as well as planned analysis are listed in Table 2.

- *Table 2 presented in the end of the text should appear here after production* -

As primary outcome, we will investigate the number of participants with unprovoked seizures, counted starting 7 days after TBI until the two years follow-up period. The presence of seizures will be clinically confirmed by a neurologist to indicate PTE. Also, as a primary outcome, the number of patients experiencing at least one serious adverse event at 24 months will be compared between placebo and biperiden groups. It is considered serious adverse event any of the following: death, life threatening event, hospitalization (initial or prolonged), disability or permanent damage, congenital anomaly/birth defect, requirement of an intervention to prevent permanent impairment or damage (devices).

For secondary outcomes, it will be considered the frequency and type of clinical seizures at each specific timepoint during the two years follow up. Also, the number of participants presenting epileptiform discharges and electrographic seizures on the electroencephalogram recording will also be analyzed. For the neuropsychological status participants will be categorized according to the specific percentile rank defined for each cited standard test. Five health-related quality of life domains (mobility, usual care, usual activities, pain/discomfort, anxiety/depression) will be covered and compared between groups according to specific and total scores.

Other outcomes include the mortality incidence and occurrence of non-serious adverse events during the two year protocol.

## Statistical analysis

The statistic plan followed the Guidelines for the Content of Statistical Analysis Plan in Clinical Trials [19].

Sample size calculation was performed based on previous studies (1, 2, 5, 6, 7, 8, 10, 20) using the Pocock formula [21]. Assuming a difference in reduction of PTE of 15% between the placebo and biperiden groups at 24 months, type 1 error (alpha) of 0,05 and type 2 error (beta) of 0.10 and 20% dropout rate, 156 patients per group would be required to provide 90% power with 5% level of significance. According to epidemiological data provided by a pilot study [10], we estimated that the inclusion pace could be 3-5 patients/center/month leading to study completion within 42 months.

The hypothesis used in the analysis is that biperiden is superior to placebo. All patients who receive at least one dose of randomized study drug will be included into the safety analysis and all patients who receive at least one dose of a randomized study drug and who has an evaluable follow-up data will be included in intention-to-treat analysis. Missing data will be handled by chained-equation multiple imputation methods considering relevant baseline and outcome variables. A complete case analysis will be presented as a sensitivity analysis.

We plan to analyze the primary outcome using logistic regression, adjusting for age and sex at baseline. The result of this regression will be an odds ratio representing the relative effect of the intervention compared to the placebo. Secondary outcomes will be analyzed using appropriate generalized linear models, including logistic and linear models. Planned subgroups include type of epileptic seizures and the presence of the polymorphic ε4 allele of the apolipoprotein E gene (Table 2).

An interim analysis will be performed at 50% recruitment considering the primary outcomes. At the interim enrollment point, the DMC will consider as criteria for early interruption of the study $p<0.001$ for safety and $p<0.0001$ for efficacy analysis. The Pocock [21] stopping boundaries, will be used as a reference rather than as a rigid rule. In addition, the DMC will also consider the effect on other secondary outcomes, in the occurrence of unpredicted adverse events and external new evidence available during trial conduction.

A confidence level of 95% will be assumed for all analyses that will be conducted using Stata v17.

# Discussion

This is a multicentric interventional prospective study aiming to evaluate the efficacy and safety of biperiden in preventing the development of epilepsy in patients that suffered TBI, in a double blind, randomized, placebo-controlled trial. If successful, it will be the first antiepileptic drug, with the advantage of having a great degree of safety, low cost and good availability given it is a medication already in use in the clinical practice and which has been extensively used for another medical condition.

The process of epileptogenesis has been the subject of numerous studies and despite some promising results in laboratory settings no compound has yet advanced to the clinical practice [22]. Both clinical and laboratory evidence suggest crucial roles for the glutamatergic and the GABAergic systems in the expression and suppression of seizures and also in the development of the epileptic condition. The cholinergic system on the other hand, raises a limited interest from the clinical perspective (with some notable exceptions such as soman and sarin) in the epilepsy arena [23]. However, recently different strategies to modulate neuronal cholinergic activity have been shown to be effective at modifying the epileptogenic process in preclinical studies [10, 12]. Among various approaches, acute treatment with biperiden, showed to hamper the development of PTE in animal models of brain injury [11].

Here we move forward by developing a clinical trial, in which biperiden will be administered in brain injured patients as soon as possible after the traumatic brain injury and only for a period of 10 days. The emergency room settings as well as the critical patient conditions (often life threatening) in addition to the long-term period (2 years) to follow up patients, imposes practical and operational hurdles for the successful development of the study. A pilot study carried out under similar conditions [10] helped to outline the main difficulties and strategies to overcome these limitations, as discussed below.

Because treatment has to be administered soon after injury, and because severe trauma patients may be unconscious and not accompanied by a legal responsible person at the hospital admission, research ethics committees from all study centers allowed an extended deadline of 48h to obtain the informed consent to the

study after beginning treatment, in order to guarantee effective enrollment rate. Also, because some aspects of the clinic history may not be readily available during enrollment, some inclusion and exclusion criteria will be checked over the initial course of treatment, which may result in high exclusion rate of patients. In that case, data from those patients will be considered only for safety analysis.

The dose of intravenous injection of biperiden is similar to the maximum recommended dose to initiate treatment of severe cases of parkinsonism [24] and also showed to be safe in critical TBI patients [10]. However, severely injured patients receive a variety of treatments and drug interventions and, in addition to the evolution of the clinical condition, all those are confounding factors that must be taken in account when registering adverse effects that might be caused by the biperiden treatment. We will focus the analysis of adverse effects on the symptoms already described for biperiden without underestimating additional unreported occurrences, mainly during treatment, and its correlation with other possible causes.

The long-term assessments in different areas (EEG, neuropsychology, etc) will be conduct by specialized personnel. To decrease loss of data associated to absence of the patient in presential visits on specified time points, e.g because social restrictions due COVID-19 pandemic or other reasons, essential data related to clinical evaluation of seizures development will be collected by phone calls, as necessary. Different contact options, such as relatives phone numbers and e-mail addresses will be collected and only after many frustrated attempts of contact for different clinical assessments, patients will be considered lost in the follow up.

The present study will focus on patients with moderate to severe TBI, comprising the group with the highest risk to develop PTE. While the pathogenesis of epilepsy development after brain injury remains unclear, the search for earlier biomarkers and for new resources that might enable to identify patients who could benefit from prophylactic treatments are additional highlights of this work. We also will investigate whether the anticholinergic actions of biperiden may also have some impact on other outcomes or sequelae after TBI. Accordingly, the present study will provide major information related to: (i) the proportion of patients who

develop PTE in addition to those who die in the two-year follow up period after TBI; (ii) other aspects of the patients` neurological outcomes that may benefit from early treatment, such as degree of disability or dependence in the daily activities, general life quality and neuropsychological dimensions; and (iii) the incidence of different ApoE polymorphisms, more importantly of APOEε4 and its putative association to epileptogenesis and treatment effectiveness.

An additional positive aspect of this project is to use an already known drug, affordable and widely used to treat another neurological pathology, being part of the list of Essential Medicines developed by the World Health Organization which indicates medicines to be always available within general health systems in adequate amounts. In the event this drug is shown to be effective in reducing epilepsy after TBI in humans it would be readily available for use as an off-label indication. Moreover, it might also have the potential to be used to mitigate the development of epilepsy secondary to other pathologies, such as after stroke.

## Trial status

Patient recruitment has not yet started**.** Estimated study start date: July 1, 2022.

# Abbreviations

APOEε4: ε4 allele of the apolipoprotein E gene

βHCG: Human chorionic gonadotropin β

CT: Computerized tomography

DMC: Data Monitoring Committee

eCRF: Electronic case report form

ECU: Emergency care unit

ECU: Emergency care unit

EEG: Electroencephalogram

EQ VAS: EuroQol visual analogue scale

EQ-5D-3L: EuroQol three-level version

FDT: Five Digit Test

GCP: Good Clinical Practice

GCS: Glasgow Coma Scale

ICU: Intensive care unit

mRS: modified Rankin Scale

PROADI-SUS: Programa de Apoio ao Desenvolvimento Institucional do Sistema Único de Saúde

PTE: Post-traumatic epilepsy

RAVLT: Rey Auditory Verbal Learning Test

REDCap: Research Electronic Data Capture

SAS: symptomatic acute seizures

TBI: Traumatic brain injury

WAIS III: Wechsler Intelligence Scale III

# Declarations

## Ethics approval and consent to participate

The BIPERIDEN study has been approved by the Comitê de Ética em Pesquisa of Sociedade Beneficente de Senhoras, Hospital Sírio-Libanês (CAAE No. 39005920.8.1001.5461). Insurance has been taken out for participants of this study to cover damage due to participation. Written informed consent will be obtained from all study participants.

## Acknowledgements

Debora Patricio Silva and Camila Santana Justo Cintra Sampaio for their support in the construction and elaboration of electronic Case Report Forms (CRFs) in the REDCap databases.

## References

1. Hauser WA, Annegers JF, Kurland LT. Prevalence of epilepsy in Rochester, Minnesota: 1940–1980. Epilepsia. 1991;32: 429–445. Doi:10.1111/j.1528-1157.1991.tb04675.x
2. Annegers JF, Hauser WA, Coan SP, Rocca WA. A population-based study of seizures after traumatic brain injuries, NEJM. 1998;338(1):20–24. Doi:10.1056/NEJM199801013380104.
3. Frey LC. Epidemiology of posttraumatic epilepsy: a critical review. Epilepsia. 2003;44(s10):11-7. doi: 10.1046/j.1528-1157.44.s10.4.x.
4. Pitkänen A, Immonen R. Epilepsy related to traumatic brain injury. Neurotherapeutics. 2014; 11(2):286-96. doi: 10.1007/s13311-014-0260-7. Doi:10.1007/s13311-014-0260-7.
5. Salazar AM, Grafman J. Post-traumatic epilepsy: clinical clues to pathogenesis and paths to prevention. Handb Clin Neurol. 2015;128:525-38. Doi:10.1016/B978-0-444-63521-1.00033-9.
6. Englander J, Bushnik T, Duong T. Analyzing risk factors for late posttraumatic seizures: a prospective multicenter investigation. Arch Phys Med Rehabil. 2003;84:365–373. Doi:10.1053/apmr.2003.50022.
7. Temkin NR, Dikmen SS, Wilensky AJ, Keihm J, Chabal S, Winn HR. A randomized, double-blind study of phenytoin for the prevention of post-traumatic seizures. NEJM. 1990;323(8):497–502. Doi: 10.1056/NEJM199008233230801.
8. Raymont V, Salazar AM, Lipsky R, Goldman D, Tasick G, Grafman J. Correlates of posttraumatic epilepsy 35 years following combat brain injury. Neurology. 2010;75: 224–229. Doi:10.1212/WNL.0b013e3181e8e6d0.

9. French JA, Bebin M, Dichter MA, Engel JJr, Hartman AL, Jóźwiak S, et al . Antiepileptogenesis and disease modification: Clinical and regulatory issues. Epilepsia. 2021;6: 483– 492. Doi:10.1002/epi4.12526.

10. Benassi SK, Alves JGSM, Guidoreni CG, Massant CG, Queiroz CM, Garrido-Sanabria E, et al. Two decades of research towards a potential first anti-epileptic drug. Seizure. 2021 Aug;90:99-109. Doi: 10.1016/j.seizure.2021.02.031.

11. Bittencourt S, Ferrazoli E, Valente MF, Romariz S, Janisset NRLL, Macedo CE, et al. Modification of the natural progression of epileptogenesis by means of biperiden in the pilocarpine model of epilepsy. Epilepsy Res. 2017;138():88-97. Doi:10.1016/j.eplepsyres.2017.10.019.

12. Meller S, Käufera C, Gailus B, Brandt C, Löscher Wolfgang. Scopolamine prevents aberrant mossy fiber sprouting and facilitates remission of epilepsy after brain injury. Neurobiol Dis. 2021, 158: 105446. Doi: 10.1016/j.nbd.2021.105446.

13. Chan AW, Tetzlaff JM, Altman DG, Laupacis A, Gøtzsche PC, Krleža-Jerić K, et al. SPIRIT 2013 statement: defining standard protocol items for clinical trials. Ann Intern Med. 2013, 158(3):200-7. Doi: 10.7326/0003-4819-158-3-201302050-00583.

14. Howick J, Webster RK, Rees JL, Turner R, Macdonald H, Price A, et al. TIDieR-Placebo: A guide and checklist for reporting placebo and sham controls. PLoS Med. 2020; 21;17(9):e1003294. doi: 10.1371/journal.pmed.1003294.

15. Teasdale G, Murray G, Parker L, Jennett B. Adding up the Glasgow Coma Score. Acta Neurochir Suppl (Wien). 1979; 28(1):13-6. Doi: 10.1007/978-3-7091-4088-8_2.

16. Harris PA, Taylor R, Thielke R, Payne J, Gonzalez N, Conde, JG. Research electronic data capture (REDCap) – A metadata-driven methodology and workflow process for providing translational research informatics support, J Biomed Inform. 2009;42(2):377-81. Doi:10.1016/j.jbi.2008.08.010.

17. Harris PA, Taylor R, Minor BL, Elliott V, Fernandez M, O'Neal L, et al, REDCap Consortium, The REDCap consortium: Building an international community of software partners, J Biomed Inform. 2019;9. Doi: 10.1016/j.jbi.2019.103208.
18. Cincura C, Pontes-Neto Octavio M, Neville IS, Mendes HF, Menezes DF, Mariano DC, et al. Validation of the National Institutes of Health Stroke Scale, Modified Rankin Scale and Barthel Index in Brazil: The Role of Cultural Adaptation and Structured Interviewing. Cerebrovasc Dis. 2009;27(2):119–122. Doi: 10.1159/000177918.
19. Gamble C, Krishan A, Stocken D, Lewis S, Juszczak E, Doré C, et al. Guidelines for the Content of Statistical Analysis Plans in Clinical Trials. JAMA. 2017 Dec 19;318(23):2337-2343. doi: 10.1001/jama.2017.18556.
20. Asikainen I, Kaste M, Sarna S. Early and late posttraumatic seizures in traumatic brain injury rehabilitation patients: brain injury factors causing late seizures and influence of seizures on long-term outcome. Epilepsia. 1999;40(5):584–589. Doi:10.1111/j.1528-1157.1999.tb05560.x.
21. Pocock SJ. Clinical Trials - a practical approach. John Wiley & Sons, Chichester - New York - Brisbane - Toronto -Singapore 1983, 265 S.
22. Saletti PG, Ali I, Casillas-Espinosa PM, Semple BD, Lisgaras CP, Moshé SL, Galanopoulou AS. In search of antiepileptogenic treatments for post-traumatic epilepsy. Neurobiol Dis. 2019;123:86-99. doi: 10.1016/j.nbd.2018.06.017.
23. Furtado MA, Rossetti F, Chanda S, Yourick D. Exposure to nerve agents: From status epilepticus to neuroinflammation, brain damage, neurogenesis and epilepsy. Neurotoxicology 2012;33(6):1476-1490. doi.org/10.1016/j.neuro.2012.09.001
24. Lerner PF. Use of biperiden hydrochloride in Parkinson's disease. South Med J. 1960;53(4):465–7. Doi: 10.1097/00007611-196004000-00015.

**Table 1.** Biperiden study plan including the schedule of enrolment, intervention, and assessments*

| | | Study Plan | | | | | | | | | | | | | | | | |
|---|---|---|---|---|---|---|---|---|---|---|---|---|---|---|---|---|---|---|
| | | **Enrolment and Intervention (Days)** | | | | | | | | | | **Follow up (Months)** | | | | | | |
| | **Timepoint after TBI** | ***1*** | ***2*** | ***3*** | ***4*** | **5** | **6** | **7** | **8** | **9** | ***10*** | ***1[a]*** | ***3[b]*** | ***6[b]*** | ***9[b]*** | ***12[b]*** | ***18[b]*** | ***24[b]*** |
| **ENROLMENT** | | | | | | | | | | | | | | | | | | |
| **Eligibility screening** | CT, ECG, βHCG | x | | | | | | | | | | | | | | | | |
| | Clinical history and demographic data | x | x | x | x | x | | | | | | | | | | | | |
| | Informed consent | x | x | | | | | | | | | | | | | | | |
| **Allocation** | | x | | | | | | | | | | | | | | | | |
| **INTERVENTION** | Study drugs iv infused | x | x | x | x | x | x | x | x | x | x | | | | | | | |
| **ASSESSMENTS** | | | | | | | | | | | | | | | | | | |
| ***Primary Outcomes*** | PTE incidence | | | | | | | | x | x | x | x | x | x | x | x | x | x |
| | Severe adverse events occurrence | x | x | x | x | x | x | x | x | x | x | x | x | x | x | x | x | x |
| ***Secondary Outcomes*** | Clinical evolution, occurrence of SAS, APOEε4 | x | x | x | x | x | x | x | x | x | x | | | | | | | |
| | Clinical and neurological assessment, mRS | | | | | | | | | | | x | x | x | x | x | x | x |
| | EEG | | | | | | | | | | | x | x | x | x | x | x | x |
| *Quality of life* | EQ-5D-3L, EQ VAS, sociodemographic data | | | | | | | | | | | | x | x | | x | | x |
| *Neuropsychologic* | WAIS (Vocabulary, Block design, Digit Symbol-Coding, Symbol Search and Digit Span) Rey–Osterrieth | | | | | | | | | | | | | x | | | | x |

| | | | | | | | | | | | | | | | | | | |
|---|---|---|---|---|---|---|---|---|---|---|---|---|---|---|---|---|---|---|
| | complex figure, RAVLT, FDT | | | | | | | | | | | | | | | | | |
| ***Other Outcomes*** | Mortality incidence | x | x | x | x | x | x | x | x | x | x | x | x | x | x | x | x | x |
| | Non-severe adverse events occurrence | x | x | x | x | x | x | x | x | x | x | x | x | x | x | x | x | x |

*Standard protocol items recommendations for Interventional Trials (SPIRIT) diagram [13].
CT computerized tomography; ECG electrocardiogram, βHCG human chorionic gonadotropin β; PTE post-traumatic epilepsy; SAS symptomatic acute seizures; EEG electroencephalography; mRS modified Rankin scale; EQ-5D-3L EuroQol three-level; EQ VAS EuroQol visual analogue scale; WAIS Wechsler Intelligence Scale; RAVLT Rey Auditory Verbal Learning Test; FDT Five Digit Test; polymorphic ε4 allele of the apolipoprotein E gene.
Time deviations in days (d) allowed for follow up visits in months(m): a 1m ± 5d; b 3, 6, 9, 12, 18, 24m ± 15d.

**Table 2. Planned analysis for primary and secondary outcomes**

| Description | Measurement | Timepoint (months) |
|---|---|---|
| **Primary Outcomes** | | |
| PTE incidence | Number of participants with unprovoked seizures | 24 |
| Serious adverse events occurrence | Number of participants experiencing at least one serious adverse event | 24 |
| **Secondary Outcomes** | | |
| PTE incidence | Number of participants with unprovoked seizures | 1, 3, 6, 9, 12, 18, 24 |
| mRS score | Number of participants categorized according to total score of the mRS scale | 1, 3, 6, 9, 12, 18, 24 |
| EEG pattern | Number of participants presenting epileptiform discharges and electrographic seizures | 1, 3, 6, 9, 12, 18, 24 |
| Neuropsychological Status | Number of participants categorized according to percentile ranks of neuropsychological tools | 6, 24 |
| Quality of life score | Number of participants categorized according to specific and total scores of quality of life scales | 3, 6, 12, 24 |
| **Other Outcomes** | | |
| Mortality incidence | Number of participants that evolved to death (all cause mortality) | 24 |
| Non-serious adverse events occurrence | Number of participants experiencing at least one non-serious adverse event | 1, 3, 6, 9, 12, 18, 24 |
| **Subgroup analysis** | Age, gender, region of lesion, occurrence of SAS, frequency and type of epileptic seizures, presence of APOEε4, educational level. | |

The data analysis is based on comparisons between biperiden x placebo groups considering the end of follow up or run-in periods. PTE post-traumatic epilepsy; EEG electroencephalography; mRS modified Rankin scale. SAS symptomatic acute seizures; APOEε4 polymorphic ε4 allele of the apolipoprotein E gene.

S1 Spirit checklist

Click here to access/download
**Supporting Information**
SPIRIT-Checklist-sumiss_PlosOne.docx
